# Linear, Electron-Rich Erbium Single-Molecule Magnet with Dibenzocyclooctatetraene Ligands

**DOI:** 10.1021/acs.inorgchem.4c00731

**Published:** 2024-05-13

**Authors:** Ernesto Castellanos, Florian Benner, Selvan Demir

**Affiliations:** Department of Chemistry, Michigan State University, 578 South Shaw Lane, East Lansing, Michigan 48824, United States

## Abstract

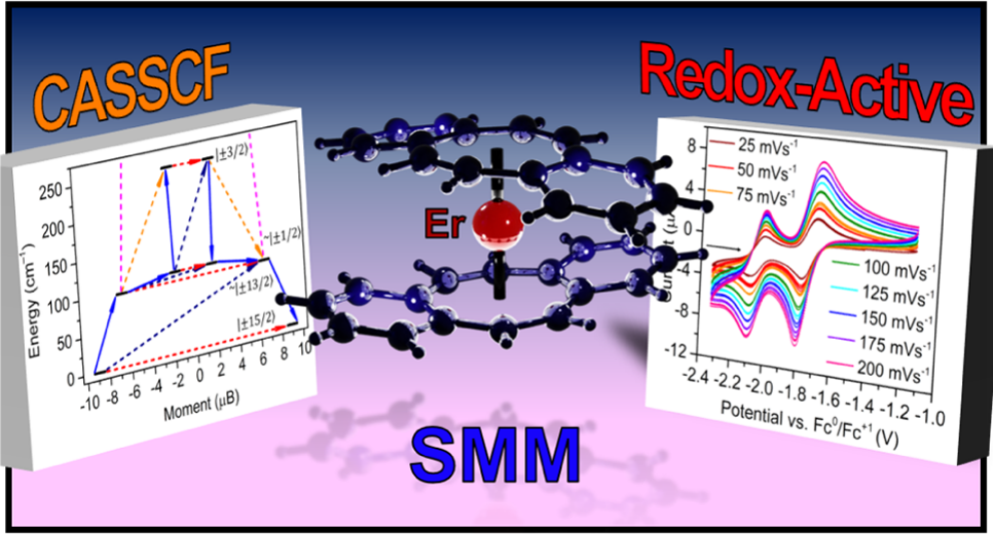

Judicious design of ligand scaffolds to highly anisotropic lanthanide ions led to substantial advances in molecular spintronics and single-molecule magnetism. Erbium-based single-molecule magnets (SMMs) are rare, which is attributed to the prolate-shaped Er^III^ ion requiring an equatorial ligand field for enhancing its single-ion magnetic anisotropy. Here, we present an electron-rich mononuclear Er SMM, [K(crypt-222)][Er(dbCOT)_2_], **1** (where dbCOT = dibenzocyclooctatetraene), that was obtained from a salt metathesis reaction of ErCl_3_ and K_2_dbCOT. The dipotassium salt, K_2_dbCOT, was generated through a two-electron reduction of the bare dbCOT^0^ ligand employing potassium graphite and was crystallized from DME to give the new solvated complex, [K(DME)]_2_[dbCOT]*_n_*, **2**. **1** was analyzed through crystallography, electrochemistry, spectroscopy, magnetometry, and CASSCF calculations. The structure of **1** consists of an anionic metallocene complex featuring a linear (180.0°) geometry with an Er^III^ ion sandwiched between dianionic dbCOT ligands and an outer-sphere K^+^ ion encapsulated in 2.2.2-cryptand. Two pronounced redox events at negative potentials allude to the formation of a trianionic erbocene complex, [Er(dbCOT)_2_]^3–^, on the electrochemical time scale. **1** shows slow magnetic relaxation with an effective spin-reversal barrier of *U*_eff_ = 114(2) cm^–1^, which is close in magnitude to the calculated energies of the first and second excited states of 96.9 and 109.13 cm^–1^, respectively. **1** exhibits waist-constricted hysteresis loops below 4 K and constitutes the first example of an erbocene-SMM bearing fused aromatic rings to the central COT ligand. Notably, **1** comprises the largest COT scaffold implemented in erbocene SMMs, yielding the most electron-rich homoleptic erbium metallocene SMM.

## Abstract 2

Merging two dibenzocyclooctatetraene (dbCOT) dianions with the prolate-shaped trivalent erbium ion led to the discovery of the first erbium single-molecule magnet (SMM) incorporating dbCOT ligands, which simultaneously constitutes the most electron-rich erbium sandwich SMM. The molecule was explored through single-crystal X-ray diffraction analysis, spectroscopy, SQUID magnetometry, and elaborate CASSCF computations.

## Introduction

Organometallic lanthanide complexes featuring planar, aromatic anionic ligands such as cyclobutadienyl (Cb), cyclopentadienyl (Cp), or cyclooctatetraenyl (COT)^1−9^ can possess bistable magnetic ground states, called single-molecule magnets (SMMs), which are relevant for potential applications in high-density information storage,^10^ quantum computing,^11^ and molecular spintronics.^12−14^ These molecules exhibit slow magnetic relaxation, reflected by an energy barrier to spin-reversal, and, if sufficiently slow, open magnetic hysteresis loops, indicative of magnetic memory similar to bulk magnets used in information storage devices. Lanthanide (Ln) ions are particularly well-suited for the development of SMMs, owing to their large magnetic anisotropy, which originates from unquenched orbital angular momentum and strong spin–orbit coupling.^15^ The shape of the overall electron density intrinsic to a given Ln^III^ ion dictates the type of ligand field required to enhance its single-ion magnetic anisotropy. The oblate Tb^III^ and Dy^III^ ions benefit from axial ligand fields and were successfully employed in mono-^16−21^ and multinuclear^22−27^ SMMs. In comparison, complexes with the prolate Er^III^ ion, which require equatorial ligand fields to enhance the magnetic anisotropy, are much less explored.^28−31^

Cp and COT scaffolds can effectively stabilize large magnetic ±*M*_J_ states of oblate and prolate Ln ions, respectively, which is a prerequisite for SMM design. For Ln ions with prolate-shaped electron densities, such as the 4f^11^ Er^III^ Kramers ion (odd electron configuration), inherent to a doubly degenerate ground state, an equatorial ligand field is beneficial as Coulomb interactions between the ligands and paramagnetic ion are minimized, augmenting the large magnetic anisotropy of Er^III^.^15^ A COT^2–^ ligand stabilizes the magnetic easy axis of the Er^III^ ion, even in the presence of peripheral ligands.^32^ Despite the advances with [Er(COT)_2_]^−^ systems, the impact of COT derivatives on crystal field (CF) splitting, and arising SMM behavior, is underexplored and prompts further elucidation.^4,5,28,33−36^ Aside from the bare COT ligand, silylated and cycloalkylated derivatives have been employed in the design of homoleptic Er^III^ SMMs. Complexes with ligands exhibiting fused aromatic substituents to the [8]annulene remain elusive. To this end, the recently isolated dibenzocyclooctatetraene (dbCOT) dianion^37−39^ serves as an ideal ligand platform to construct Er sandwich complexes to potentially advance both the area of SMMs and COT metallocene chemistry. Here, we present the synthesis of the first homoleptic erbium metallocene complex bearing two dbCOT ligands, [K(crypt-222)][Er(dbCOT)_2_], **1**, which is a single-molecule magnet under zero dc fields. **1** was in-depth-characterized by a myriad of techniques to devise a full picture of the origins of magnetic relaxation. To this end, dc and ac magnetic susceptibility data were collected, and *ab initio* calculations were performed.

## Experimental Section

### General Information

All manipulations were performed in either an argon- or nitrogen-filled MBraun glovebox with an atmosphere of <0.1 ppm of O_2_ and <0.1 ppm of H_2_O. House nitrogen was purified through an MBraun HP-500-MO-OX gas purifier. Tetrahydrofuran (THF) was predried by refluxing over potassium for several days, distilled, and stirred over Na/K alloy for final purification. Dimethoxyethane (DME) and toluene were dried over potassium, and ^*n*^hexane was dried over calcium hydride. All solvents were distilled under N_2_ from their drying agents, and water/oxygen absence was confirmed via benzophenone solution as an indicator within a glovebox prior to use. Deuterated solvents were purchased from Cambridge Isotope Laboratories, dried over Na/K alloy for several days, and filtered prior to use. Anhydrous ErCl_3_ was purchased from Sigma-Aldrich and used as received. 2.2.2-Cryptand (crypt-222) was purchased from Sigma-Aldrich and crystallized from hot ^*n*^hexane prior to use. Tetrabutylammonium hexafluorophosphate [^*n*^Bu_4_N][PF_6_] was purchased from Sigma-Aldrich and crystallized several times before use. Dibenzocyclooctatetraene (dbCOT)^40^ and potassium graphite (KC_8_)^41^ were prepared according to literature procedures. Elemental analysis was performed at Michigan State University using a PerkinElmer 2400 Series II CHNS/O analyzer.

### Synthesis of [K(crypt-222)][Er(dbCOT)_2_], **1**

In a 20 mL vial charged with a stir bar, ErCl_3_ (40.7 mg, 0.149 mmol, 1 equiv) was dissolved in an 8 mL THF solution at 50 °C after 20 min. K_2_dbCOT (81.9 mg, 0.290 mmol, 2 equiv) was dissolved in 2 mL of THF and added dropwise, resulting in an immediate precipitation of insoluble colorless solids, presumably potassium chloride, and the color of the reaction changed progressively from dark orange to red-brown. The mixture was stirred for 24 h and then centrifuged and filtered over celite. The filtrate was treated with 2.2.2-cryptand (56.0 mg, 0.149 mg, 1 equiv) and allowed to stir for 10 min before evaporating to dryness. The obtained red-brown solids were washed first with toluene and then with ^*n*^hexane. Dark red-brown, block-shaped crystals of **1**, suitable for single-crystal X-ray diffraction analysis, were obtained from a concentrated THF solution at −35 °C over the course of 3 days. The crystals were separated from the mother liquor, dried under vacuum, washed with DME, and dried further under reduced pressure for the elemental, spectroscopic, electrochemical, and magnetic analysis of **1**. Crystalline yield: 67.6 mg, 0.068 mmol, 46%. ^1^H NMR (500 MHz, THF-*d*_8_, 25 °C) δ (ppm): 39.67 (br), 38.00 (br), 37.19 (br), 1.09 (br), −77.83 (br). Due to the paramagnetic nature of **1**, no signals were observed in the ^13^C NMR spectrum. IR (cm^–1^): 3026w, 2949w, 2883w, 2875w, 2807w, 1476w, 1460w, 1443w, 1386s, 1354m, 1294m, 1261m, 1238w, 1213w, 1173w, 1148m, 1130m, 1102s, 1076s, 1017m, 1004m, 949m, 931w, 830w, 818w, 774s, 706w, 688w, 660w. Anal. Calcd for C_50_H_60_N_2_O_6_KEr: C: 60.58; H, 6.10; N: 2.83. Found: C: 60.78; H: 6.21; N: 2.83.

### Synthesis of [K(DME)]_2_[dbCOT]*_n_*, **2**

To a 250 mL round-bottom flask charged with a stir bar, dbCOT (3.1247 g, 15.297 mmol, 1 equiv) was added, dissolved in 7 mL of DME, and subsequently diluted to 35 mL. To the clear and colorless stirring solution, KC_8_ (5.3747 g, 39.760 mmol, 2.6 equiv) was added portionwise over the course of 5 min, which resulted in an immediate color change to a dark violet-red solution with insoluble black solids, presumably graphite. Following the full addition of KC_8_, the reaction mixture was diluted with an additional 10 mL of DME to ensure adequate stirring and was allowed to proceed for 24 h. The resulting dark violet-red solution was filtered using a glass frit. The collected black solids were washed with four 20 mL fractions of fresh DME. The clear, dark violet-red filtrate was evaporated to dryness under reduced pressure to yield purple solids, which gradually became green in color after drying to a constant mass. Dark red, block-shaped crystals, suitable for single-crystal X-ray diffraction analysis, were obtained from a concentrated DME solution at −35 °C over the course of 24 h. The crystals were separated from the mother liquor and dried under high vacuum for 5 h until green in color and at a constant mass. The mother liquor was subsequently evaporated to dryness and crystallized from a concentrated DME solution at −35 °C, yielding purple crystalline solids in 24 h. A combination of multiple crystallization crops afforded crystalline purple solids, which were dried, crushed to a fine powder, and dried further until green in color. Crystalline yield: 3.1031 g, 10.986 mmol, 72%. ^1^H NMR (500 MHz, THF-*d*_8_, 25 °C) δ (ppm): 7.89–7.87 (AA′BB′, 4H, benzo-*H*^C^), 7.81 (s, 4H, COT-*H*^A^), 6.21–6.19, (AA′BB′, 4H, benzo-*H*^B^). ^13^C NMR (126 MHz, THF-*d*_8_, 25 °C) δ (ppm): 135.42 (benzo-*C*^4,7,12,15^), 109.02 (COT-*C*^3,8,11,16^), 108.60 (benzo-*C*^5,6,13,14^), 95.48 (COT-*C*^1,2,9,10^). IR spectra (cm^–1^): 3015w, 2980w, 1416w, 1371s, 1174m, 1144m, 1095m, 1353m, 1001s, 930w, 852w, 759s, 704m. Anal. Calcd for C_16_H_12_K_2_: C, 68.03; H, 4.28; N, 0.00. Found: C, 67.48; H, 4.64; N, 0.04.

### Single-Crystal X-ray Diffraction Analysis

A red-brown, block-shaped crystal and dark red, block-shaped crystal with dimensions 0.323 × 0.266 × 0.098 mm^3^ and 0.307 × 0.105 × 0.069 mm^3^ of **1** and **2**, respectively, were mounted on Nylon loops using Paratone oil. Data for **1** and **2** were collected on an XtaLAB Synergy, Dualflex, HyPix diffractometer equipped with an Oxford Cryosystems low-temperature device, operating at *T* = 100.00(10) K. Data for **1** and **2** were measured using ω scans using Mo Kα and Cu Kα radiations (microfocus sealed X-ray tube, 50 kV, 1 mA), respectively. The total number of runs and images was based on the strategy calculation from the program CrysAlisPro (Rigaku, V1.171.41.90a, 2020), which was used to retrieve and refine the cell parameters as well as for data reduction. A numerical absorption correction based on Gaussian integration over a multifaceted crystal model empirical absorption correction using spherical harmonics was implemented in the SCALE3 ABSPACK scaling algorithm. The structures of **1** and **2** were solved in the space groups *C*2/*c* and *P*1̅, respectively, by using intrinsic phasing with the ShelXT structure solution program.^42^ The structures were refined by least-squares using version 2018/2 of XL^42^ incorporated in Olex2.^43^ All non-hydrogen atoms were refined anisotropically. Hydrogen atom positions were calculated geometrically and refined by using the riding model.

### Infrared Spectroscopy

IR spectra were recorded with an Agilent Cary 630 Fourier-transform infrared spectrometer on crushed crystalline solids under a nitrogen atmosphere.

### NMR Spectroscopy

NMR spectra were recorded at 25 °C on a Varian 500 MHz Inova spectrometer and calibrated to the residual solvent signals (THF-*d*_8_: δ_H_ = 3.58 ppm, δ_C_ = 67.6 ppm). Signal multiplicities are abbreviated as s (singlet) and br (broad), and all NMR samples were prepared in a nitrogen-filled glovebox using J. Young NMR tubes. Atom labels correspond to those given in Figure S8.

### UV–vis Spectroscopy

The UV–vis spectrum was collected with an Agilent Cary 60 spectrophotometer at ambient temperature from 250 to 800 nm. Samples were prepared in an argon-filled glovebox and measured in a 1 cm quartz cuvette, outfitted with a Teflon screw cap. The spectrum was baseline-corrected from a sample of dry THF.

### Electrochemistry

Cyclic voltammograms were measured in THF with [^*n*^Bu_4_N][PF_6_] (250 mM) as a supporting electrolyte and a 3 mM analyte. A Metrohm Autolab PGSTAT204 potentiostat with a glassy-carbon working electrode, a platinum wire pseudo-reference electrode, and a platinum wire counter electrode were used. All voltammograms were measured in an argon-filled glovebox and externally referenced to a ferrocene solution of identical supporting electrolyte concentration.

### Magnetic Susceptibility Measurements

Magnetic susceptibility data were obtained on a Quantum Design MPMS3 Superconducting Quantum Interference Device (SQUID) magnetometer. The magnetic sample of **1** was prepared by saturating and covering dried, crushed crystalline solids (13.4 mg, 1.4 × 10^–5^ mol) with ample molten eicosane (at 60 °C) to prevent crystallite torquing and to provide good thermal contact between the sample and the bath. The sample was sealed in an airtight container and transferred to the magnetometer. All data were corrected for diamagnetic contributions from the eicosane, and core diamagnetism was estimated using Pascal’s constants.^44^

### Computational Methods

The magnetic properties of **1** were calculated via a Complete Active Space Self-Consistent Field (CASSCF) + N-Valence Electron Perturbation Theory (NEVPT2) approach using the ORCA 5.0.4 software.^45,46^ Scalar relativistic effects were taken into account via the Douglas–Kroll approach, where the DKH-def2-SVP basis set was used for H atoms,^47^ DKH-def2-TZVP for C atoms,^48^ and SARC2-DKH-QZVP/SARC2-DKH-QZVP/JK basis sets for the Er atom.^49^ Auxiliary basis sets for C and H were generated via the autoaux feature.^50^ Tight convergence criteria were employed throughout with an energy convergence tolerance of 1e-07. The computation of Fock matrices and gradient/Hessian integrals was accelerated by using the RI-JK approximation.^51^ The frozen core approximation was switched off for all of the calculations. Throughout all calculations, a finer integration grid (defgrid3) was employed. The calculations were carried out on the crystallographic coordinates of [Er(dbCOT)_2_]^−^, using an active space comprising the 11 4f electrons of Er^III^ in seven orbitals. 35 quartet roots and 112 doublet roots were considered for the state-averaged (SA) CASSCF calculation. Dynamic correlation effects were introduced into the spin-free SA-CASSCF wave function via strongly contracted NEVPT2 (SC-NEVPT2).^52−54^ The construction of the fourth-order reduced density matrix was simplified via the efficient implementation (D4step efficient).^55,56^ Spin–orbit coupling (SOC) effects were included within the NEVPT2 step via quasi-degenerate perturbation theory (QDPT) using the mean-field/effective potential Hamiltonian RI-SOMF(1×).^57−59^ The free-particle Foldy–Wouthuysen (fpFW) transformation was carried out in the first step of the DKH protocol by including the vector potential. Picture change corrections were included in the second order, as well as finite nucleus corrections.^60^ Lastly, the magnetic properties such as *g* tensors, crystal field parameters, and the estimated single-ion anisotropy barrier were calculated via the SINGLE_ANISO standalone program.^61^ Calculated magnetization and susceptibility curves were scaled such that they align with the experimental room-temperature χ_M_*T* and maximum field *M* values.

## Results and Discussion

**Figure 1:**
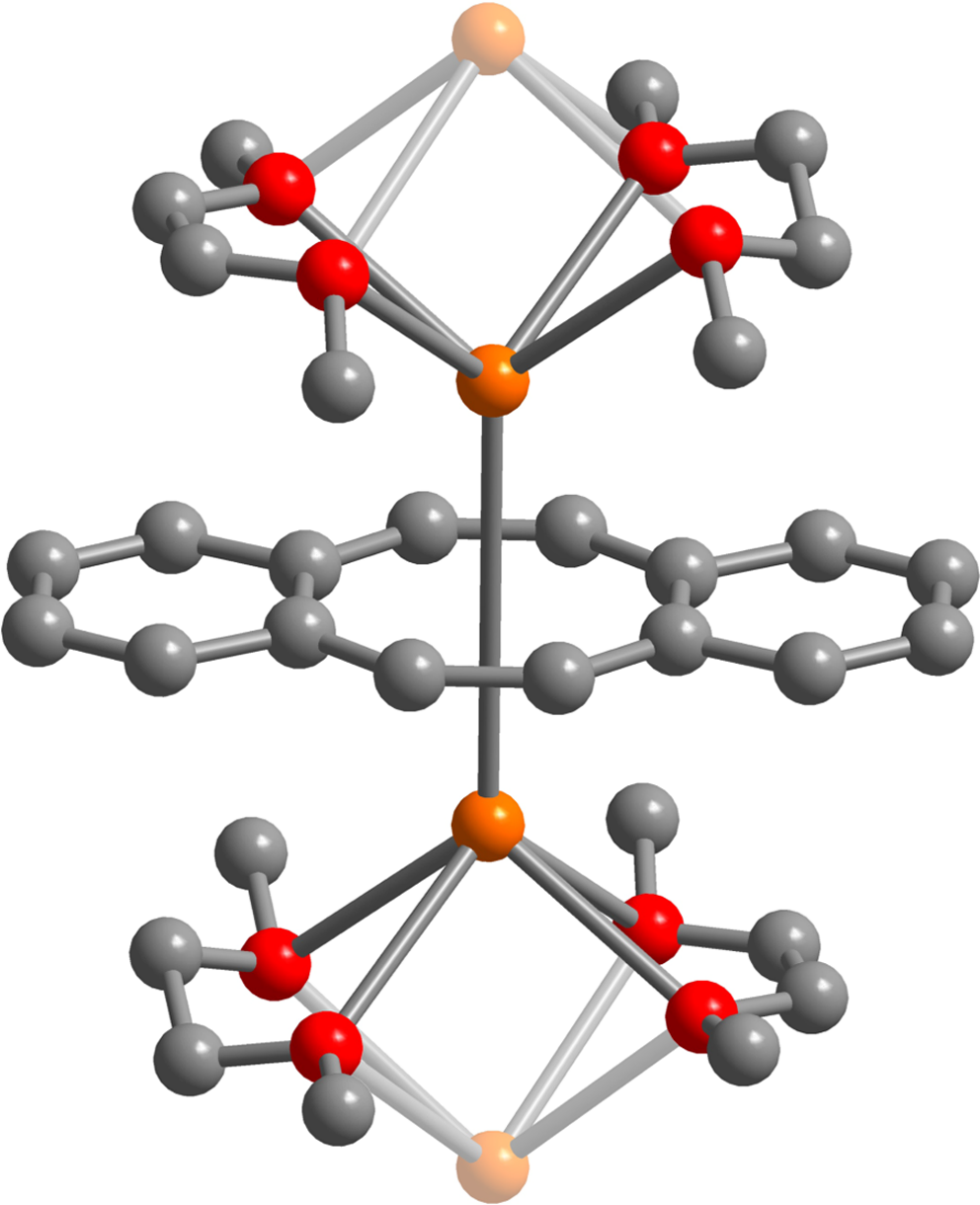
Structure of the monomeric [K(DME)]_2_[dbCOT] unit in a crystal of [K(DME)]_2_[dbCOT]*_n_*, **2**. Orange, red, and gray spheres represent K, O, and C atoms, respectively. Bridging potassium ions were faded for clarity. Hydrogen atoms have been omitted for clarity.

**Figure 2:**
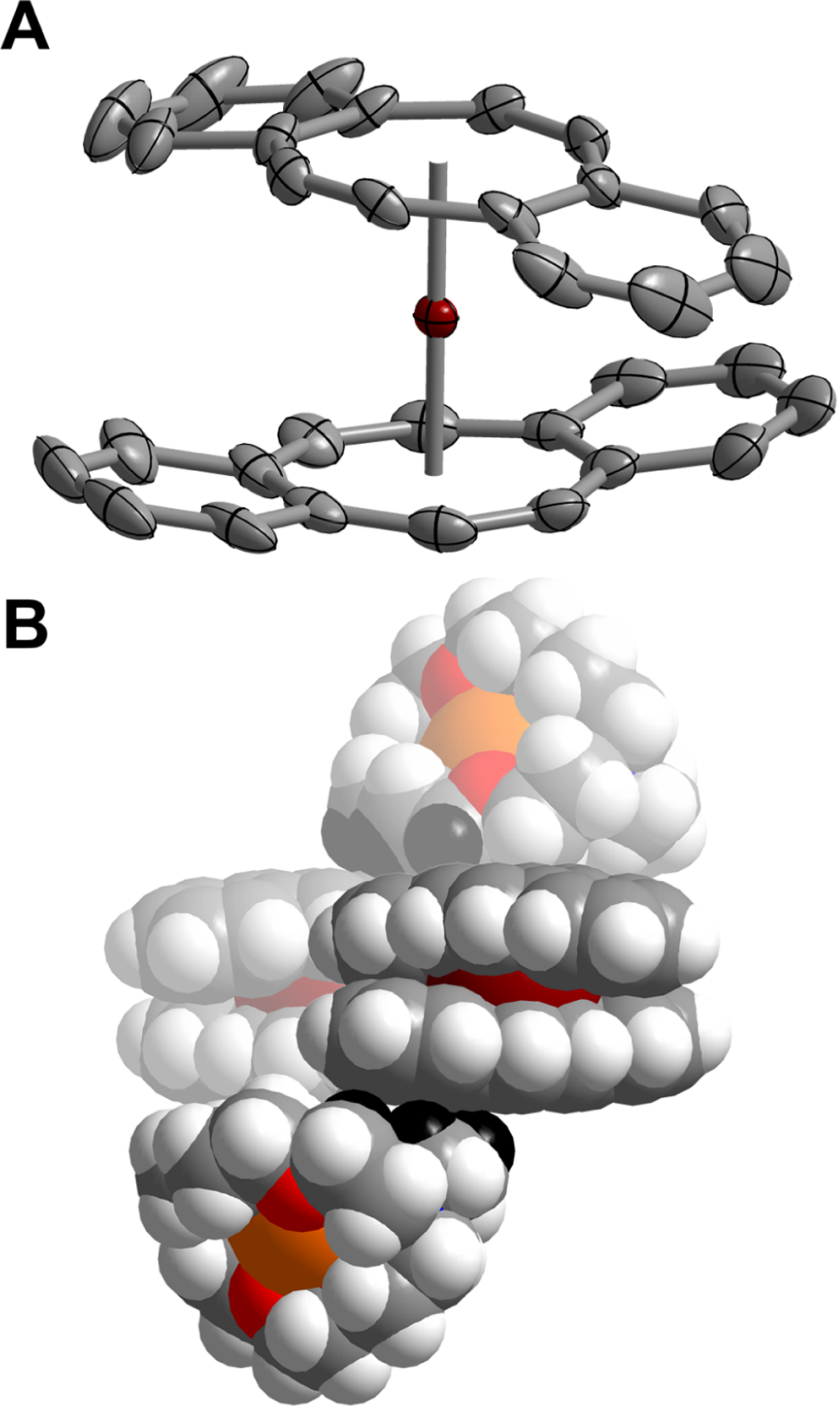
(A) Structure of the [Er(dbCOT)_2_]^−^ anion in a crystal of [K(crypt-222)][Er(dbCOT)_2_], **1**, with thermal ellipsoids drawn at 50% probability level. Maroon and gray spheres represent Er and C atoms, respectively. The [K(crypt-222)]^+^ countercation, hydrogen atoms, and solvent molecule (in the crystal lattice) have been omitted for clarity. (B) Space-filling model depicting the crystal packing of **1**. H atoms of the [K(crypt-222)]^+^ countercation with close contacts to the (dbCOT)^2–^ ligands in **1** are highlighted in black. Maroon, orange, red, blue, gray, and gray-white spheres represent Er, K, O, N, C, and H atoms, respectively. The orientation of the molecules in this space-filling model was chosen in favor of highlighting the crystal packing effect but precludes the visibility of the nitrogen atoms of the [K(crypt-222)]^+^ countercation; however, those are apparent in the ball-and-stick model in Figure S1. Select distances (Å) and angles (deg) in **1**: Er–C_COT_: 2.577(1)–2.647(1); Er–Cnt: 1.837, 1.832; Er–Er: 9.621(1)–10.854(1); and Cnt–Er–Cnt: 180.0.

The dimethoxyethane (DME) adduct [K(DME)]_2_[dbCOT]*_n_*, **2**, was obtained in a 72% crystalline yield from the two-fold reduction of the bare dbCOT^0^ ligand with excess potassium graphite, and after workup, crystallization from DME at −35 °C. Dark red, block-shaped crystals suitable for single-crystal X-ray diffraction analysis were grown from a concentrated DME solution at −35 °C over the course of 24 h. The solid-state structure of **2** is a one-dimensional network of planar (dbCOT)^2–^ ligands sandwiched between two η^8^-coordinating K^+^ ions, which in turn are bridged through all oxygen atoms of two DME molecules to the adjacent K^+^ ion (Figures 1 and S7–S9). The chemical two-electron reduction of the neutral and antiaromatic 16π-electron dbCOT^0^ ligand elicits a significant structural reorganization to form a planar, aromatic 18π-electron dianion. Each [K(DME)]_2_[dbCOT] unit of the chain is rigorously linear with a K–Cnt–K angle (where Cnt = COT ring centroid) of 180.0° and C–C distances consistent with the THF adducts of the dianionic M_2_dbCOT salts of the alkali metals (where M = Li, Na, K, Rb, Cs) (Figure S8). The homoleptic, anionic bis(dibenzocyclooctatetraenyl)erbate metallocene, [K(crypt-222)][Er(dbCOT)_2_], **1**, was isolated by extending the previously reported synthetic route used to access the analogous yttrocene complex, [K(crypt-222)][Y(dbCOT)_2_].^62^ The salt metathesis reaction of ErCl_3_ with K_2_dbCOT, in the presence of 2.2.2-cryptand, in THF at 50 °C (eq 1), affords dark red-brown, block-shaped crystals of **1** suitable for single-crystal X-ray diffraction analysis from a concentrated THF solution at −35 °C over the course of 3 days in a 46% yield (Figure 2A). The erbocene sandwich complex, **1**, crystallizes in the *C*_2_/*c* space group, isostructural with [K(crypt-222)][Y(dbCOT)_2_], and features a trivalent erbium ion ligated by two dianionic (η^8^-dbCOT) ligands. **1** is comprised of charge-separated ion pairs, owing to the presence of an encapsulated potassium cation, [K(crypt-222)]^+^. Resultingly, the potassium ion remains 8.290(1) Å from the nearest Er^III^ ion, and the Er–Er distances range from 9.621(1) to 10.854(1) Å, significantly larger than the Er–K and Er–Er distances observed for [Er(COT)_2_]^−^.^4,5,29,34,36^ The Er–C_COT_ distances in **1** vary between 2.577(1) and 2.647(1) Å, consistent with that of other homoleptic Er-COT complexes.^3−5,33,34,36,63^ The coordinated dbCOT ligands exhibit a remarkably linear geometry, as evidenced by a Cnt–Er–Cnt angle of 180.0°. The Er–Cnt distances of 1.837 and 1.832 Å are in agreement with those of COT-based erbocene complexes (Table S3).^4,5,33−35^ Despite the presence of aromatic ligands in **1**, the benzo rings of the dbCOT ligands deviate from planarity, bending inward toward the Er^III^ ion, resulting from a combination of crystal packing effects, electrostatic repulsion between adjacent atoms of [K(crypt-222)]^+^ and [dbCOT]^2–^ ligands in **1**, and electrostatic attraction of the ligand π-system toward the highly Lewis acid Er^III^ ion (Figure 2B).
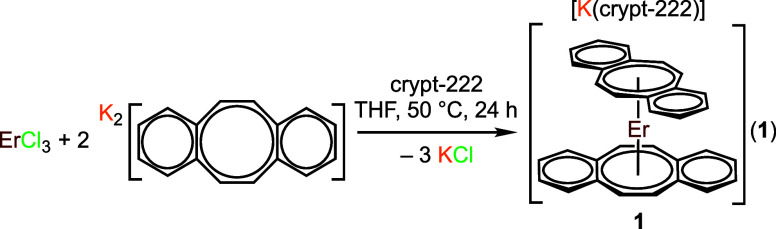
1

### Spectroscopic Studies

**Figure 3:**
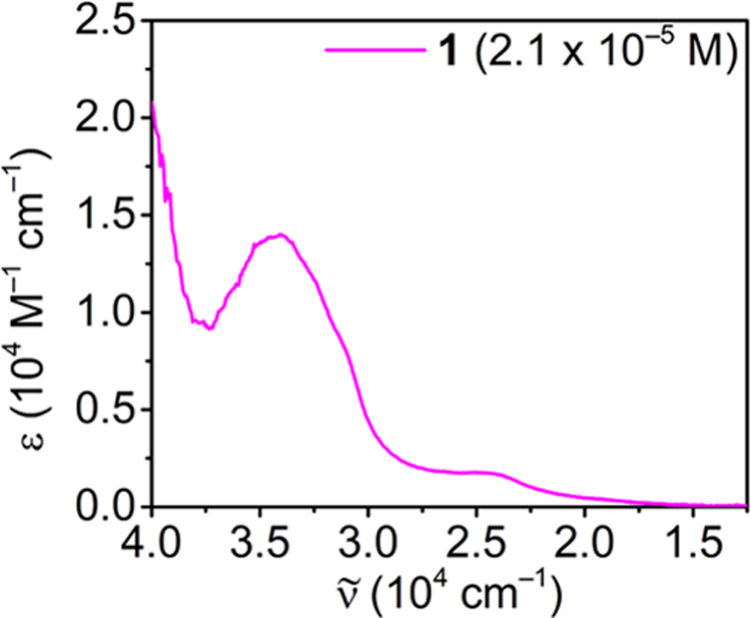
UV–vis spectrum of [K(crypt-222)][Er(dbCOT)_2_], **1**, measured in THF at a concentration of 2.1 × 10^–5^ mol/L.

The solution state structure of **1** was investigated by nuclear magnetic resonance (NMR) and electronic absorption spectroscopy. Owing to the paramagnetic nature of Er^III^, a 200 ppm spectral window between 100 and −100 ppm was used to monitor the ^1^H spectrum of **1**, which yielded five broad proton resonances between 39.67 and −77.83 ppm (Figure S10). The ^13^C NMR spectrum of **1** displayed no carbon resonances between 220 and −20 ppm, possibly attributed to the lower sensitivity of the ^13^C nucleus and rapid relaxation induced by the paramagnetic lanthanide center (Figure S11). By contrast, the 1D and 2D NMR spectra of **2** revealed aromatic signals, consistent with the expected aromatic character of **2** following a 2-fold reduction of dbCOT^0^ (Figures S12–S15). The UV–vis spectrum of **1** is similar to that of [K(crypt-222)][Y(dbCOT)_2_]^62^ and exhibits two broad absorption maxima at 2.40 × 10^4^ and 3.41 × 10^4^ cm^–1^ (Figure 3). The high-energy transition in the UV region arises from π–π* transitions of the dianionic dbCOT ligand, consistent with the ligand–ligand charge-transfer bands for dianionic COT^2–^ and dbCOT^2–^ ligands.^64^ The broad absorption feature in the near-visible region is significantly lower in intensity and can be tentatively interpreted as a ligand-to-metal charge transfer. The solid-state structure of **1** was probed via infrared (IR) spectroscopy, which displays sharp absorptions of varying strength in the fingerprint region arising from C–C and C–H stretching and bending modes (Figure S16). Similarly, the IR spectrum of **2** is dominated by sharp absorptions between 700 and 1500 cm^–1^, which is also attributed to the C–C and C–H stretching and bending modes (Figure S17).

### Electrochemical Studies

**Figure 4:**
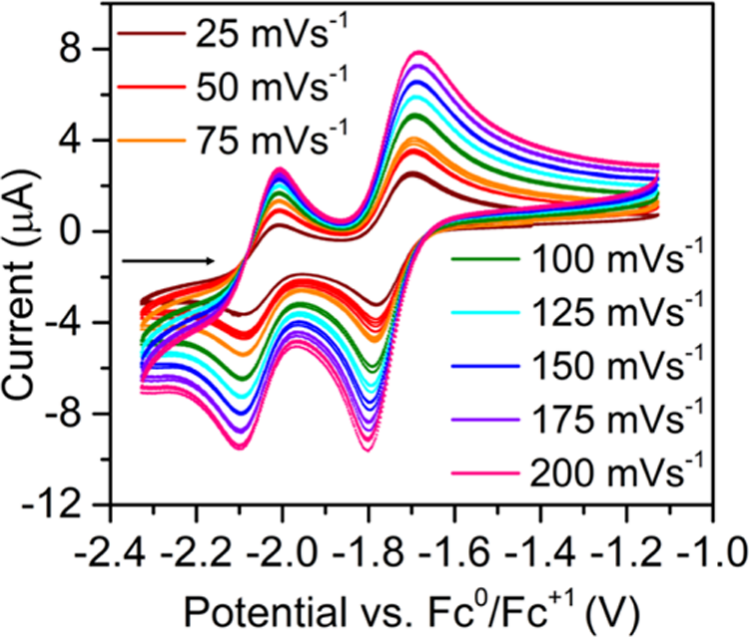
Cyclic voltammograms of [K(crypt-222)][Er(dbCOT)_2_], **1** (3 mM), measured in a 250 mM [^*n*^Bu_4_N][PF_6_] THF solution recorded at scan rates between 25 and 200 mVs^–1^ in 25 mVs^–1^ intervals. Two quasi-reversible features are found at −1.741 ± 0.108 V, *E*_1_, and −2.050 ± 0.108 V, *E*_2_.

The redox activity of **1** was investigated via cyclic voltammetry (CV) experiments, using a THF solution of [^*n*^Bu_4_N][PF_6_] as a supporting electrolyte. Initial electrochemical measurements revealed four redox events between −2.1 and −0.1 V vs Fc^0^/Fc^+^, with two features closely grouped at lower potentials below −1.7 V and two events toward positive potentials above −0.5 V (Figures S18–S20). The two reproducible redox events at −1.741 ± 0.084 V, *E*_1_, and −2.050 ± 0.084 V, *E*_2_, exhibit a quasi-reversible character owing to the persistence of both redox couples after multiple scans. Thus, to elucidate the reversibility of these events, variable scan rate CV was undertaken (Figure 4 and Table 1). The feature at −1.741 ± 0.081 V has a large peak-to-peak separation between *E*_cp_ (*E*_cp_ = cathodic peak potential) and *E*_ap_ (*E*_ap_ = anodic peak potential), which increases from 84 to 115 mV with increasing scan rate, alluding to the presence of a formally irreversible electrochemical process. This electron-transfer process can be assigned to the dbCOT^2–/3–•^ redox couple and is in agreement with the generation of a trianionic dbCOT radical ligand on the electrochemical time scale.^2,62^ In addition to the average peak/current ratio (*i*_pc_/*i*_pa_) of −1.185, determined across all scan rates, the peak/current function exhibits a subtle scan rate dependence, furthering the notion that the observed dbCOT^2–/3–•^ redox couple in **1** is electrochemically irreversible. The reproducible uptake and release of electrons at the electrode surface suggests, however, that access to the trianionic oxidation state of dbCOT is a chemically reversible process. By contrast, the subsequent redox feature at an average *E*_1/2_ of −2.050 ± 0.081 V, *E*_2_, displays a narrower change in *E*_cp_ – *E*_ap_ separation from 82 to 92 mV s^–1^ with an increasing scan rate. However, the peak/current function is evidently dependent on the scan rate and exhibits an asymmetric peak current ratio, confirming that this redox couple is undoubtedly electrochemically irreversible. This is consistent with the large negative anodic current observed, suggesting an unfavorable and slow electron-transfer process, which requires larger applied potentials to attain measurable current flows.^65^ This pronounced difference between electron-transfer processes may allude to the generation of either a divalent Er^II^ ion or the uptake of an additional electron onto a dbCOT ligand, generating a trianionic erbocene complex, [Er(dbCOT)_2_]^3–^, on the electrochemical time scale. Toward more positive potentials, two irreversible features at −0.526 ± 0.084 and −0.113 ± 0.084 V can be ascribed to the oxidation of the dianionic dbCOT ligands in **1** (Figure S19). These ligand-centered oxidations generate dbCOT^2–/1–•^ and dbCOT^1–•/0^ redox couples, respectively. In an attempt to recover the reversibility of these redox features, variable scan rate CV measurements were also performed; however, no reductive features were observed following the electrochemical oxidation of **1** at multiple scan rates (Figure S20). Thus, the observed irreversibility is in accordance with the chemical transformations monitored upon the exposure of homoleptic COT-based metallocenes to mild oxidants.^2,66,67^

**Table 1:** Electrochemical Potentials of [K(crypt-222)][Er(dbCOT)_2_], 1, vs Fc^0^/Fc^+1^^a^

	25 mV s^–1^	50 mV s^–1^	75 mV s^–1^	100 mV s^–1^	125 mV s^–1^	150 mV s^–1^	175 mV s^–1^	200 mV s^–1^	average
*E*_1_									
*E*_c_ (V)	–1.739	–1.739	–1.741	–1.742	–1.744	–1.742	–1.743	–1.740	–1.741
*E*_a_ (V)	–1.823	–1.828	–1.833	–1.840	–1.844	–1.850	–1.855	–1.855	–1.841
*E*_c_ – *E*_a_ (V)	0.084	0.089	0.093	0.0985	0.098	0.100	0.111	0.115	0.100
*E*_1/2_ (V)	–1.739	–1.739	–1.741	–1.742	–1.744	–1.742	–1.743	–1.740	–1.741
*i*_c_/*i*_a_	–1.282	–1.141	–1.203	–1.185	–1.163	–1.163	–1.170	–1.172	–1.185
*i*_p_/*n*^1/2^	1.599	1.572	1.447	1.607	1.667	1.690	1.7321	1.757	1.634
*E*_2_									
*E*_c_ (V)	–2.049	–2.047	–2.048	–2.050	–2.050	–2.051	–2.053	–2.054	–1.841
*E*_a_ (V)	–2.131	–2.127	–2.131	–2.137	–2.134	–2.141	–2.145	–2.146	–2.050
*E*_c_ – *E*_a_ (V)	0.082	0.080	0.082	0.087	0.084	0.090	0.092	0.092	0.086
*E*_1/2_ (V)	–2.049	–2.047	–2.049	–2.050	–2.050	–2.051	–2.053	–2.051	–2.050
*i*_c_/*i*_a_	–13.085	–5.004	–4.059	–3.834	–3.608	–3.510	–3.511	–3.484	–5.012
*i*_p_/*n*^1/2^	0.176	0.408	0.489	0.532	0.569	0.587	0.596	0.606	0.495

a E_c_ = cathodic peak position, *E*_a_ = anodic peak position, *E*_c_ – *E*_a_ = peak-to-peak separation, *E*_1/2_ = half-wave potential, *i*_c_/*i*_a_ = anodic-to-cathodic peak current ratio, *i*_p_ = peak current, *i*_p_/*n*^1/2^ = peak current function.

### Magnetic Measurements

**Figure 5:**
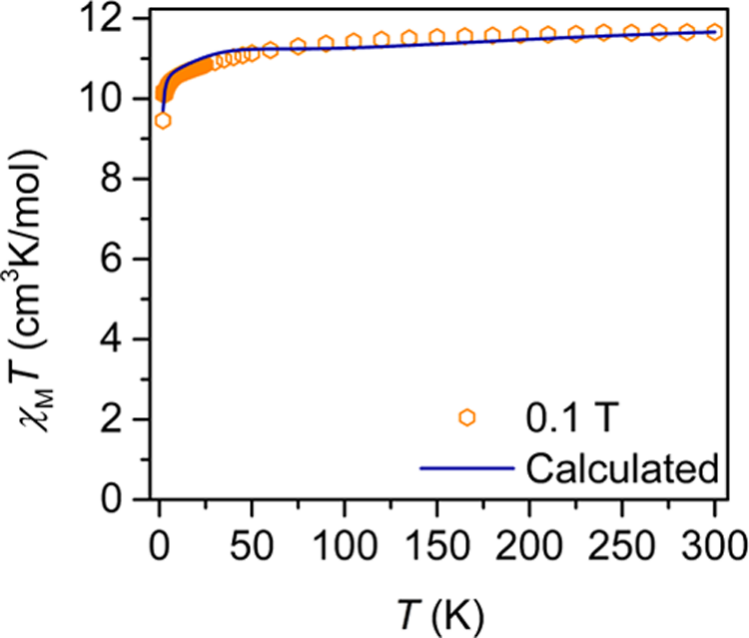
Variable-temperature dc magnetic susceptibility data of [K(crypt-222)][Er(dbCOT)_2_], **1**, collected under a 0.1 T applied dc field (orange hexagons). Solid dark blue line represents the *ab initio*-calculated susceptibility curve for the [Er(dbCOT)_2_]^−^ anion.

To glean further insight into the static magnetic behavior of [K(crypt-222)][Er(dbCOT)_2_], **1**, the temperature-dependent molar magnetic susceptibilities were measured from 2 to 300 K under 0.1, 0.5, and 1.0 T dc fields (Figures 5 and S21–S24). At 0.1 T, the room-temperature χ_M_*T* value of 11.66 cm^3^ K mol^–1^ agrees well with the presence of an uncoupled Er^III^ ion (Er^III^ = ^4^*I*_15/2_, *S* = 3/2, *L* = 6, *J* = 15/2, *g* = 6/5, (χ_M_*T*)_calc_ = 11.48 cm^3^ K mol^–1^).^68^ At higher fields, the room-temperature χ_M_*T* value drops slightly to 11.37 and 11.34 cm^3^ K mol^–1^ for 0.5 and 1.0 T, respectively. Under a 0.1 T field, the χ_M_*T* value decreases gradually to 10.14 cm^3^ K mol^–1^ at 3.2 K, before dropping steadily to 9.46 cm^3^ K mol^–1^ at 2 K, where the decline in the χ_M_*T* value is attributed to the depopulation of excited states. The field-dependent magnetization data of **1** at 2 K increases rapidly to 5.61 Nμ_B_, and the reduced magnetization data exhibits non-superimposable curves between 2 and 10 K, owing to the presence of substantial magnetic anisotropy or low-lying excited states (Figures S25 and S26). Magnetization values of this magnitude have been observed for other Er complexes innate to a ligand sphere favoring prolate lanthanide ions.^28,31,69^

**Figure 6:**
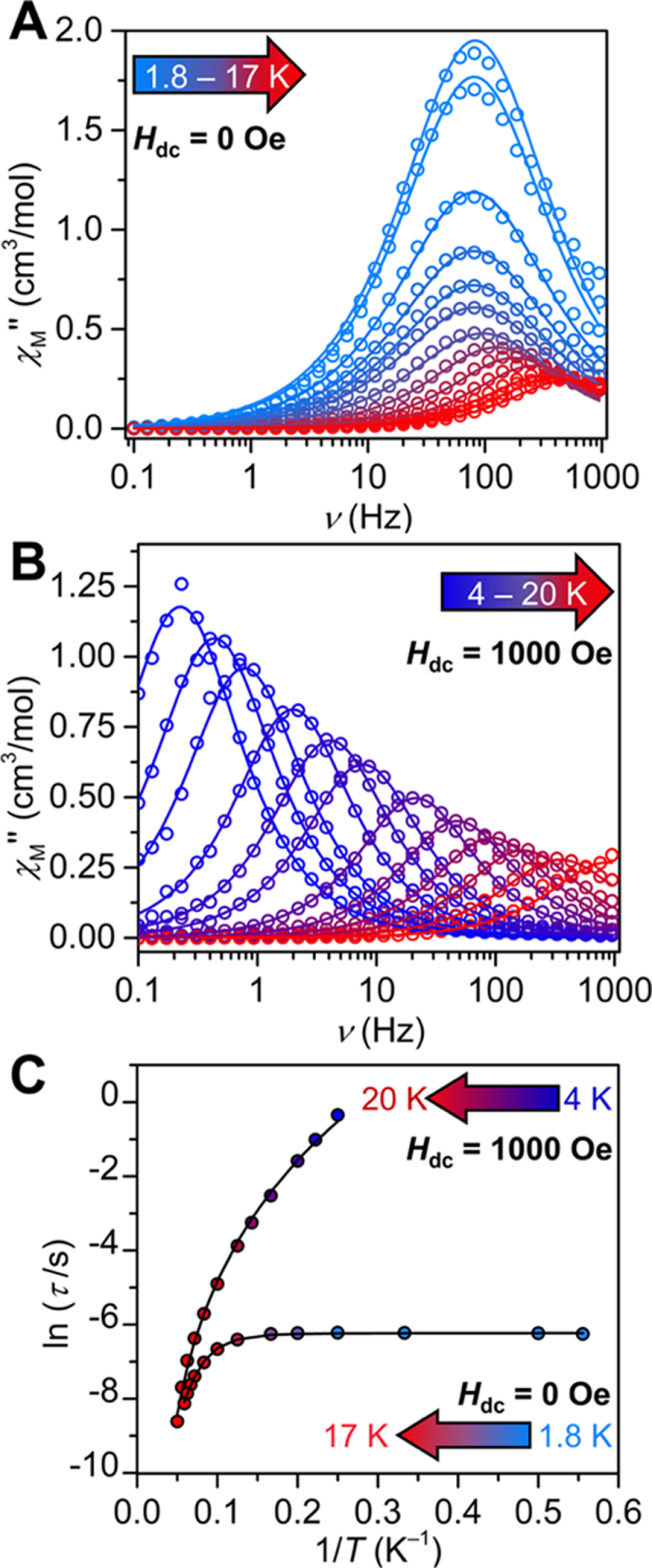
(A) Variable-temperature, variable-frequency out-of-phase (χ_M_″) components of the ac magnetic susceptibility for [K(crypt-222)][Er(dbCOT)_2_], **1**, under a zero applied dc field from 1.8 K (blue circles) to 17 K (red circles) and (B) under a 1000 applied dc field from 4 K (dark blue circles) to 20 K (dark red circles). Solid lines represent fits of the data to a generalized Debye model. (C) Comparison of relaxation time data, τ, vs the inverse temperature for **1** under zero and 1000 Oe applied dc fields. The black solid lines represent fits to Orbach and Raman relaxation processes under a 1000 Oe dc field yielding *U*_eff_ = 114(2) cm^–1^, τ_0_ = 2.1(1) × 10^–7^ s, *C* = 2.5(1) × 10^–3^, and *n* = 4.7(2), and Raman and QTM processes under a zero applied dc field affording *C* = 9.49(1.3) s^–1^K^–*n*^, *n* = 4.44(9), and τ_QTM_^–1^ = 1.98(1.0) × 10^–3^ s.

To probe whether **1** is an SMM, ac magnetic susceptibility measurements were carried out between 0.1 and 1000 Hz. Under a zero Oe applied dc field, **1** exhibits out-of-phase (χ_M_″) ac magnetic susceptibility signals between 1.8 and 17 K, verifying that **1** exhibits slow magnetic relaxation (Figures 6A, S27, and S28). In the absence of an applied dc field, two distinct regimes are observed above and below 10 K. Between 1.8 and 10 K, the peak maximum remains at 81 Hz, where this temperature-independent maximum decreases in intensity with increasing temperature, which is an indication for quantum tunneling of the magnetization (QTM).^68^ This trend has been observed in COT-based Er SMMs.^31−33,69−71^ Importantly, the large distances between the paramagnetic metal centers observed in **1** suggests that the onset of fast relaxation pathways such as QTM may be intrinsic to the crystal field (CF) effects engendered by the (dbCOT)^2–^ scaffold or [K(crypt-222)]^+^ countercation^33^ and not due to weak dipolar interactions of nearby neighboring Er ions.^3,4,72^ Above 10 K, the peak maximum shifts toward higher frequencies until 17 K, where the maximum moves past 1000 Hz, and this temperature-dependent behavior alludes to the presence of a thermally activated process. Fitting the entire temperature range to Raman and QTM processes,^200^ according to eq S1 afforded the parameters *C* = 9.49(1.3) s^–1^ K^–*n*^, *n* = 4.44(9), and τ_QTM_^–1^ = 1.98(1.0) × 10^–3^ s (Figures 6C, S29, and S30). The presence of fast relaxation pathways, such as QTM, can be effectively suppressed through the application of a static dc magnetic field.^68^ Thus, ac magnetic susceptibility measurements were undertaken at 1.8 K with applied dc fields ranging from 0 to 1500 in 500 Oe increments (Figure S31). At 500 Oe, the out-of-phase (χ_M_″) peak maximum remained largely constant at 81 Hz; however, with decreased intensity at the higher field, and additionally, an onset of a χ_M_″ peak at lower frequencies was observed. Increasing the field to 1000 Oe changed the shape of the χ_M_″ frequency scan even further and importantly eliminated the high-frequency peak. By comparison, the shape of the χ_M_″ frequency scan at 1500 Oe is largely invariant. Thus, variable-temperature ac magnetic susceptibility measurements were performed under a 1000 Oe dc field, which showed much stronger temperature dependencies than those that were monitored at zero field (Figures 6B and S32). Starting at 4 K, the maximum at 0.23 Hz shifts toward higher frequencies with increasing temperature until 20 K (Figure 6B), enabling a quantitative analysis of the magnetic relaxation times through the construction of a Cole–Cole plot (Figure S33). Each temperature could be subsequently fit to a generalized Debye model, and the resulting data was used to construct an Arrhenius plot. A satisfactory fit to the experimental data over the entire probed temperature range was achieved considering an Orbach and Raman relaxation process, with an energy barrier to spin-reversal of 114(2) cm^–1^ and a τ_0_ value of 2.1(1) × 10^–7^ s (Figures 6C, S34, and S35). The obtained fits closely resemble the *ab initio* computed energies of the first and second excited states of 96.9 and 109.13 cm^–1^, respectively. A linear fit of the three highest temperature points to an Orbach process yielded only an effective spin-reversal barrier, *U*_eff_, of 90.29 cm^–1^ and an attempt time, τ_0_, of 2.94 × 10^–7^ s (Figure S36). The effective energy barrier observed in **1** is smaller than that found in other COT-based SMMs,^4,5,33^ which may arise from several factors innate to the bis-dbCOT scaffold. The presence of a homoleptic COT-based framework generates long Er–Cnt distances exceeding 1.8 Å, where long (>1.7 Å) Er–Cnt distances have been correlated to lower *U*_eff_ values (Tables S4–S6).^69,73^ In addition, the presence of fused aromatic rings to the central COT ligand in dbCOT engenders a substantial decrease in molecular symmetry and an increased π-donation ability in comparison to COT and COT″ (where COT″ = 1,4-bis(trimethylsilyl)cyclooctatetraenyl), possibly contributing to enhanced equatorial CF effects, which may generate more pronounced QTM.^73^

**Figure 7:**
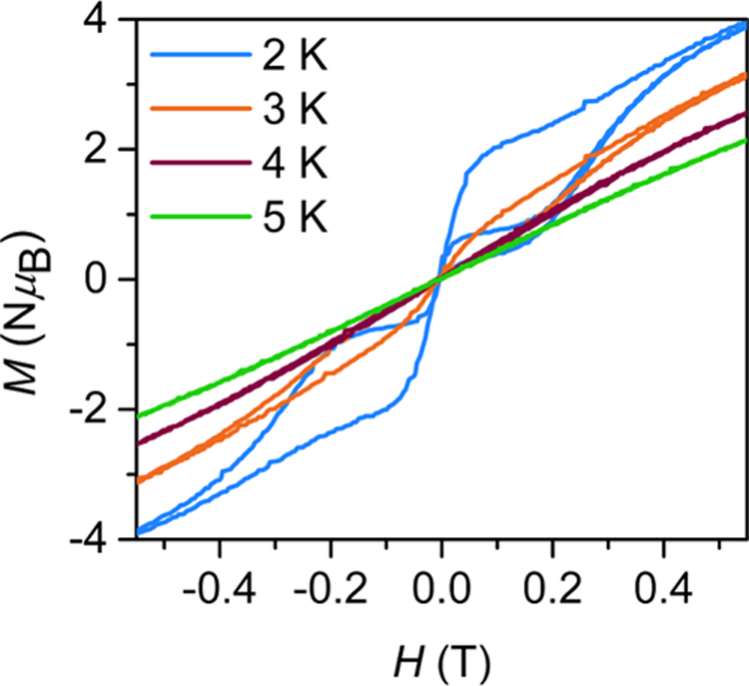
Variable-field magnetization (*M*) data vs dc magnetic field (*H*) for [K(crypt-222)][Er(dbCOT)_2_], **1**, collected at an average sweep rate of 0.01 T/s between −0.6 and +0.6 T. Blue, orange, purple, and green lines represent 2, 3, 4, and 5 K, respectively.

The retention of magnetization in the absence of an applied magnetic field is a key benchmark for SMM performance and their prospective application toward magnetic memory storage devices. Thus, variable-field magnetization measurements were conducted from 1.8 to 5 K, with an average sweep rate of 0.01 T/s (Figures 7 and S37). At temperatures below 4 K, **1** exhibits waist-constricted hysteresis loops, where the magnetization at near-zero fields drops precipitously resulting in a lack of remanent magnetization (*M*_R_ = 0 μ_B_ mol^–1^). The occurrence of ground-state QTM is in line with the ac magnetic susceptibility data collected at a zero dc field. It is also in conformity with field-dependent magnetization behavior of homo- and heteroleptic SMMs bearing COT ligands.^4,32−34,74,75^ The large Er–Er separation in **1** precludes the relaxation originating from weak intermolecular dipolar interactions. Thus, diamagnetic dilutions are unlikely to improve the SMM performance.

### *Ab Initio* Calculations

The magnetic hysteresis loops of **1** are closed at zero field where this behavior is markedly different from other homoleptic Er^III^ sandwich complexes containing COT ligands (see Table S4). In principle, these compounds have an identical first coordination sphere to **1** but are distinct in their second coordination sphere. A factor that might impact the SMM behavior is the relative orientation of the dbCOT^2–^ ligands and the degree of eclipsing among the C_COT_ atoms. Somewhat similar influences were detected for [K(18-*c*-6)][Er(COT)_2_] and [K(18-*c*-6)(THF)_2_][Er(COT)_2_], where the former is innate to the orthorhombic space group *Pnma* with eclipsed COT^2–^ rings, while the latter adopts the triclinic space group *P*1̅ with staggered COT^2–^ rings.^4^ Intriguingly, the experimental data suggested no significant change in the SMM properties for undiluted samples. This was later evidenced via multireference calculations on [K(18-crown-6)][Er(COT)_2_], where the real *D*_8*d*_ symmetry with staggered COT^2–^ C atoms and the idealized *D*_8*h*_ symmetry with eclipsed C atoms revealed essentially unchanged low-lying energy spectra.^5^

To elucidate the dynamic magnetic properties observed for **1**, we carried out *ab initio* calculations on the [Er(dbCOT)_2_]^−^ anion employing a CASSCF/NEVPT2/QDPT approach with the Orca 5.0.4 program suite (see the Experimental Section for a detailed description). The magnetic properties were obtained via the SINGLE_ANISO standalone program.^61^ Importantly, the *ab initio* calculations provide an invaluable reference frame for the crystal field effects induced by the dbCOT^2–^ anion onto trivalent lanthanide ions relative to COT^2–^.

The energy spectrum was calculated for the eight lowest-lying Kramers doublets (KDs). Distinguishable intra-KD transitions of either QTM or thermally assisted QTM processes (TA-QTM), as well as inter-KD transitions, which proceed via Orbach and/or Raman mechanisms, could be identified. The probability for each transition is given by the transition dipole moments (Table S9).

**Figure 8:**
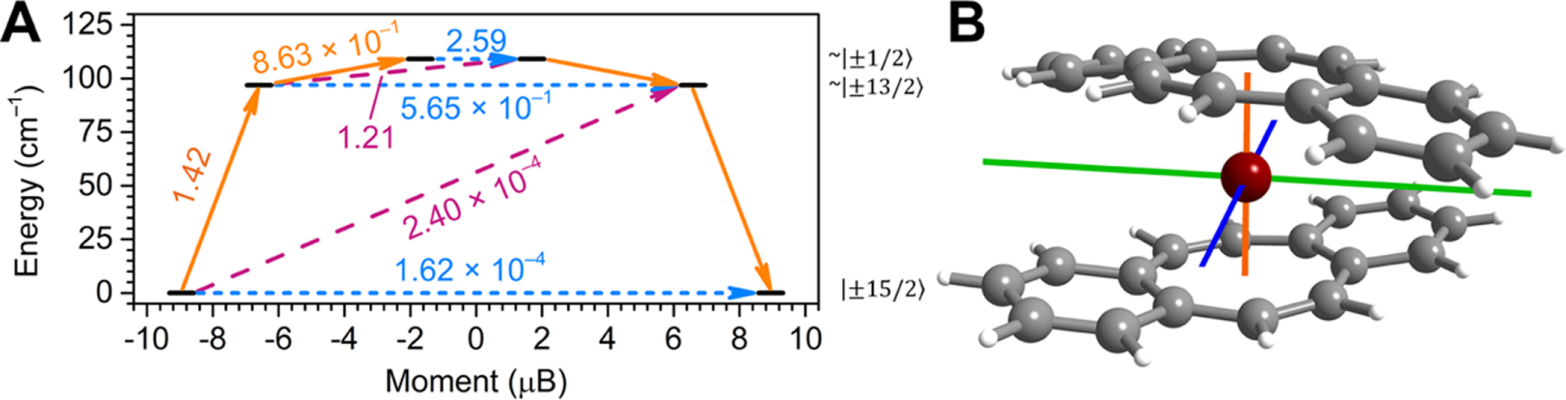
(A) Calculated blocking barrier of [K(crypt-222)][Er(dbCOT)_2_], **1**. Blue dotted lines indicate intra-KD transitions between |±*M*_J_⟩ states via quantum tunneling of the magnetization (QTM) or thermally assisted QTM (TA-QTM). Orange and purple lines represent inter-KD transitions between |*M*_J_⟩ and |±*M*_J+1_⟩ via Orbach and/or Raman processes. Values on the arrows correspond to the respective transition magnetic moment matrix elements. The full barrier comprising the eight lowest-lying KDs is depicted in Figure S38. (B) Plot of the calculated *g*-tensor components of the ground-state Kramers doublet of the [Er(dbCOT)_2_]^−^ anion in a crystal of [K(crypt-222)][Er(dbCOT)_2_], **1**. Color code: *g*_x_(blue), *g*_y_ (green), and *g*_z_ (orange) with the respective compositions 0.000 (*g*_x_), 0.000 (*g*_y_), and 17.895 (*g*_z_).

Similarly to homoleptic COT complexes, the ground-state KD (KD1) of **1** comprises a pure *M*_J_ = |±15/2⟩ composition with a strongly uniaxial *g*-tensor dominated by *g*_z_ (17.895) and negligible *g*_x_/*g*_y_ components. Consequently, the intra-KD transition magnetic moment is negligibly small, rendering the ground-state QTM improbable (Figure 8 and Table S9). Comparable to other COT derivatives, the ground-state anisotropy axis of **1** is aligned through the COT ring of the dbCOT ligands (Figure 8B). The first excited KD (KD2), consisting of an ∼0.83:0.15 mixture of |±13/2⟩ and |±1/2⟩ states, is substantially lower in energy relative to [Er(COT)_2_]^−^ (181 cm^–1^). Furthermore, the admixture of the |±1/2⟩ state prompts non-negligible transversal magnetic moments, as expressed through considerable *g*_x_ (1.333) and *g*_y_ (2.055) contributions relative to *g*_z_ (13.178). Such large transverse magnetic fields result in a significantly increased probability for the intra-KD transition, albeit slightly smaller than the probability for the KD2 → KD3 transition. Notably, the transition dipole moment connecting KD+2 and KD–3 is the largest, which indicates that the dominant relaxation pathway operative in **1** is through the second excited state. KD3 comprises a ∼0.83:0.15 mixture of |±1/2⟩ and |±13/2⟩ and the transverse *g* components are even more pronounced compared to KD2 (*g*_x_ = 3.394, *g*_y_ = 5.720, *g*_z_ = 9.778). The intra-KD transition magnetic moment is predominant for this state. Notably, the separation between the first and second excited KDs is extremely small (12.5 cm^–1^), which explains the accessibility of the KD+2 → KD–3 cross-barrier process. The experimentally determined spin-reversal barrier of 114(2) cm^–1^ for **1** is in excellent agreement with the calculated energies of the first and second excited states of 96.9 and 109.13 cm^–1^, respectively. In summary, this confirms the above-described relaxation pathway.

To further verify the conducted calculations, we simulated the χ_M_*T* data for **1** collected from 2 to 300 K at 0.1, 0.5, and 1.0 T, and the field-dependent magnetization data collected from 2 to 10 K at 0 to 7 T fields (Figures 5 and S39–S41). The simulated values coincide excellently with the experimental data.

Notably, the Er–Cnt distances are shorter in **1** relative to other homoleptic Er–COT complexes (1.832 and 1.837 Å in **1** vs 1.848–1.904 Å in Er–COT examples; see Table S4). This hints at a stronger electrostatic interaction between the lanthanide ion and the more electron-rich dbCOT^2–^ dianions, which is attributed to the electron-donating nature of the peripheral phenyl rings. Such effects are proposed to give a markedly larger splitting of the lowest-lying energy manifold.^73^ Indeed, when comparing the eight lowest-lying Kramers doublet energies of the Er^III^ ion in **1** with the energy spectrum of [K(18-crown-6)][Er(COT)_2_], a significantly larger splitting is found for **1** (687 cm^–1^) over the [Er(COT)_2_]^−^ anion (515 cm^–1^).^5^ This indeed suggests a stronger crystal field imposed by dbCOT^2–^ over COT^2–^ anions. However, the presence of two COT-type ligands in **1** generates competing electrostatic interactions between the dbCOT ligands and the Er^III^ ion, diminishing the uniaxiality of excited states. A similar reasoning was deduced by comparing ligand field effects within [Er(COT)2]^−^ and (DSP)ErCOT (where DSP = 3,4-dimethyl-2,5-bis(trimethylsilyl)phospholyl).^69^

Thus, the calculations highlight the potential of the dbCOT^2–^ ligand for the design of Er^III^-based SMMs in that the highest angular momentum *M*_J_ = |±15/2⟩ state is stabilized and a large crystal field splitting is elicited. The substantially smaller separation between the first and second excited states, along with a strong mixing between the |±13/2⟩ and |±1/2⟩ states, proposes the pursuit of heteroleptic mononuclear complexes innate to a different local symmetry around the metal ion. Specifically, coupling the [Er(dbCOT)]^+^ scaffold to a softer ligand may enable the favorable interplay of contracted Er–Cnt distances (<1.7 Å) and crystal field to unleash the full potential of the dbCOT^2–^ building block.

## Conclusions

The synthesis of a new dianionic COT derivative, [K(DME)]_2_[dbCOT]*_n_*, **2**, in the form of an alkali-metal DME adduct has been accomplished. **2** was crystallographically characterized and successfully employed in the realm of single-molecule magnetism. The stoichiometric reaction of **2** and ErCl_3_ generated [K(crypt-222)][Er(dbCOT)_2_], **1**, corresponding to the first example of a homoleptic lanthanide metallocene complex bearing dianionic dbCOT ligands. The anionic motif of **1** also serves as the first example of a homoleptic COT-based Er metallocene featuring fused aromatic rings to the central COT ligand, charge-balanced with an outer-sphere [K(crypt-222)]^+^ countercation. On the electrochemical time scale, **1** features quasi-reversible and irreversible redox processes where events at lower potentials (2.050 ± 0.108 V vs Fc^0^/Fc^1+^) suggest the generation of a trianionic erbium complex, [Er(dbCOT)_2_]^3–^, possibly attributed to the formation of a divalent erbium metallocene. Excitingly, **1** is an SMM with an energy barrier to magnetization reversal, *U*_eff_, of 114(2) cm^–1^ and a pre-exponential factor, τ_0_, of 2.1(1) × 10^–7^ s, and magnetic hysteresis loops below 4 K, which places **1** among the much smaller sets of erbium-based SMMs than constructed with oblate tripositive lanthanides. Remarkably, **1** constitutes the first erbium SMM incorporating a dbCOT ligand and, simultaneously, the most electron-rich erbium metallocene SMM bearing the yet largest employed COT derivative. Quantum chemical calculations uncovered a pure *M*_J_ = |±15/2⟩ ground state, with the calculated energy of the first and second excited states of 96.9 and 109.13 cm^–1^, respectively, closely matching the experimental barrier height. The calculations suggest that different local metal environments employing dbCOT^2–^ may slow the relaxation, further improving the overall magnetic performance. An exciting avenue forward are heteroleptic complexes that match a dbCOT^2–^ dianion with other carbon- and nitrogen-based ligands to fine-tune the crystal field toward next-generation SMMs.

## Supporting Information Available

Electrochemical, spectroscopic, magnetic, and computational data, as well as detailed crystallographic information for **1** and **2** (PDF)
