# Supplementary material for: Linear, Electron-Rich Erbium Single-Molecule Magnet with Dibenzocyclooctatetraene Ligands
Source: Inorg Chem. 2024 May 13;63(21):9888–98. doi: 10.1021/acs.inorgchem.4c00731 (PMC11134505; doi:10.1021/acs.inorgchem.4c00731)
Supplement: Supplementary file 1 — ic4c00731_si_001.pdf [file ic4c00731_si_001.pdf]

Supporting Information

for

Linear, Electron-Rich Erbium Single-Molecule Magnet with Dibenzocyclooctatetraene Ligands

Ernesto Castellanos, Florian Benner, and Selvan Demir*

Department of Chemistry, Michigan State University, 578 South Shaw Lane, East Lansing, Michigan 48824, USA

*Correspondence to: sdemir@chemistry.msu.edu (S.D.)

Inorg. Chem.

Table of Contents

1 **X-Ray Crystallography**……S5

Table S1. Crystallographic data and structural refinements of $[K(crypt\text{-}222)][Er(dbCOT)_2]\cdot 2(OC_4H_8)$, **1**•$2(OC_4H_8)$, and $[K(DME)]_2[dbCOT]_n$, **2**..S5

Fig. S1. Structure of $[K(crypt\text{-}222)][Er(dbCOT)_2]$, **1**……S6

Fig. S2. Structure of $[Er(dbCOT)_2]^{-}$, **1**……S7

Fig. S3. Space filling model of $[Er(dbCOT)_2]^{-}$, **1**……S8

Fig. S4. Unit cell of $[K(crypt\text{-}222)][Er(dbCOT)_2]$, **1**……S9

Fig. S5. Crystal packing diagram of $[K(crypt\text{-}222)][Er(dbCOT)_2]$, **1**……S10

Fig. S6. Crystal packing diagram of $[Er(dbCOT)_2]^{-}$, **1**……S11

Fig. S7. Polymeric structure of $[K(DME)]_2[dbCOT]_n$, **2**……S12

Fig. S8. Structure of $[dbCOT]^{2-}$, **2**……S13

Fig. S9. Unit cell of $[K(DME)]_2[dbCOT]^{2-}$, **2**……S14

Table S2 Metrical parameters for C–C distances in $[K(crypt\text{-}222)][Er(dbCOT)_2]$, **1**……S15

Table S3 Metrical parameters for C–C distances in $[K(crypt\text{-}222)][Er(dbCOT)_2]$, **1**……S16

2 **NMR Spectroscopy**……S17

Fig. S10. ^{1}H NMR spectrum of $[K(crypt\text{-}222)][Er(dbCOT)_2]$, **1**……S17

Fig. S11. ^{13}C NMR spectrum of $[K(crypt\text{-}222)][Er(dbCOT)_2]$, **1**……S18

Fig. S12. ^{1}H spectrum of $[K(DME)]_2[dbCOT]_n$, **2**……S19

Fig. S13. ^{1}H–^{1}H gCOSY NMR spectrum of $[K(DME)]_2[dbCOT]_n$, **2**……S20

Fig. S14. ^{13}C NMR spectrum of $[K(DME)]_2[dbCOT]_n$, **2**……S21

Fig. S15. ^{1}H–^{13}C gHSQCAD spectrum of $[K(DME)]_2[dbCOT]_n$, **2**……S22

3 **IR Spectroscopy**……S23

Fig. S16. FTIR spectrum of $[K(crypt\text{-}222)][Er(dbCOT)_2]$, **1**……S23

Fig. S17. FTIR spectrum of $[K(DME)]_2[dbCOT]_n$, **2**……S24

4 **Cyclic Voltammetry**……S25

Fig. S18. Cyclic voltammogram of [K(crypt-222)][Er(dbCOT)$_2$], **1**, measured between –2.4 and 0.1 V……………………………………………...**S25**

Fig. S19. Cyclic voltammogram of [K(crypt-222)][Er(dbCOT)$_2$], **1** measured between –1.0 and 0.3 V……………………………………………...**S26**

Fig. S20. Cyclic voltammogram of [K(crypt-222)][Er(dbCOT)$_2$], **1** measured between –1.0 and 0.3 V at variable scan rates………………………**S27**

5 Magnetic Data……..………………………………………………………...**S28**

Table S4. Single-molecule magnet behavior of homoleptic, mononuclear cyclooctatetraenyl-based Er complexes……………………………………**S28**

Table S5. Single-molecule magnet behavior of heteroleptic, mononuclear cyclooctatetraenyl-based Er complexes……………………………………**S30**

Table S6. Single-molecule magnet behavior of mononuclear cyclooctatetraenyl-based Er complexes containing heterocycles……………..**S32**

Fig. S21. Variable-temperature dc magnetic susceptibility data of [K(crypt-222)][Er(dbCOT)$_2$], **1** at 0.1 T……………………………………**S34**

Fig. S22. Variable-temperature dc magnetic susceptibility data of [K(crypt-222)][Er(dbCOT)$_2$], **1** at 0.5 T……………………………………**S35**

Fig. S23. Variable-temperature dc magnetic susceptibility data of [K(crypt-222)][Er(dbCOT)$_2$], **1** at 1.0 T……………………………………**S36**

Fig. S24. Variable-temperature dc magnetic susceptibility data of [K(crypt-222)][Er(dbCOT)$_2$], **1** at 0.1, 0.5, and 1.0 T……………………**S37**

Fig. S25. Field-dependent magnetization data of [K(crypt-222)][Er(dbCOT)$_2$], **1**………………………………………………………**S38**

Fig. S26. Reduced magnetization data of [K(crypt-222)][Er(dbCOT)$_2$], **1**……...**S39**

Fig. S27. In-phase (χ_M') and out-of-phase (χ_M'') components of the ac magnetic susceptibility for [K(crypt-222)][Er(dbCOT)$_2$], **1**, under zero applied dc field……………………………………………………………...**S40**

Fig. S28. Cole-Cole plots for [K(crypt-222)][Er(dbCOT)$_2$], **1**, under zero applied dc field………………………………………………………………**S41**

Fig. S29. Arrhenius plot of [K(crypt-222)][Er(dbCOT)$_2$], **1**, under zero applied dc field with fits to Raman and QTM processes……………………**S42**

Fig. S30. Arrhenius plot of [K(crypt-222)][Er(dbCOT)$_2$], **1**, under zero applied dc field with sum fit to Raman and QTM processes…………………**S43**

Fig. S31. Out-of-phase (χ_M'') components of the ac magnetic susceptibility for [K(crypt-222)][Er(dbCOT)$_2$], **1**, at 1.8 K under applied dc fields between 0 and 1500 Oe……………………………………………………………………..…**S44**

Fig. S32. In-phase (χ_M') and out-of-phase (χ_M'') components of the ac magnetic susceptibility for [K(crypt-222)][Er(dbCOT)$_2$], **1**, under a 1000 Oe dc field………………………………………………………………..……………**S45**

Fig. S33. Cole-Cole plots for [K(crypt-222)][Er(dbCOT)$_2$], **1**, under a 1000 Oe applied dc field………………………………………..……………**S46**

Fig. S34. Arrhenius plot for [K(crypt-222)][Er(dbCOT)$_2$], **1**, under a 1000 Oe applied dc field with Orbach and Raman fits……………………………………...**S47**

Fig. S35. Arrhenius plot of [K(crypt-222)][Er(dbCOT)$_2$], **1**, under zero applied dc field with sum fit to Orbach and Raman processes……………..…..…**S48**

Fig. S36 Arrhenius plot for [K(crypt-222)][Er(dbCOT)$_2$], **1**, under a 1000 Oe applied dc field with Orbach fit between 16 and 20 K……………………………..**S49**

Fig. S37. Variable-field magnetization data for [K(crypt-222)][Er(dbCOT)$_2$], **1**, at 1.8 K collected from –1 and 1 T………………**S50**

6 ***Ab initio* Calculations**…………………………………………………..…....**S51**

Table S7. Calculated Kramers doublet (KD) energies, associated magnetic moments, *g*-tensors, and wavefunction composition for Er(dbCOT)$_2$]$^-$, **1**……...**S51**

Table S8. Calculated crystal field parameters for Er(dbCOT)$_2$]$^-$, **1**……………...**S51**

Table S9. Calculated average transition dipole moments for Er(dbCOT)$_2$]$^-$, **1**……………………………………………………………………**S52**

Fig. S38. Calculated relaxation barrier for Er(dbCOT)$_2$]$^-$, **1**………..……………**S53**

Fig. S39. Variable-temperature dc magnetic susceptibility data of [K(crypt-222)][Er(dbCOT)$_2$], **1** at 0.5 T with *ab initio* calculated values…….......**S54**

Fig. S40. Variable-temperature dc magnetic susceptibility data of [K(crypt-222)][Er(dbCOT)$_2$], **1** at 1.0 T with *ab initio* calculated values…….......**S55**

Fig. S41. Field-dependent magnetization data of [K(crypt-222)][Er(dbCOT)$_2$], **1**, with the *ab initio* calculated values…………..……**S56**

7 **References**……….………………………………………………………...**S57**

1 X-ray Crystallography

Table S1. Crystallographic data and structural refinements of [K(crypt-222)][Er(dbCOT)$_2$]·2(OC_4H_8), **1**·2(OC_4H_8), and [K(DME)]$_2$[dbCOT]$_n$, **2**.

	**1**·2(OC_4H_8)	**2**
CCDC Accession Codes	2334063	2334064
Empirical formula	$C_{58}H_{76}KN_2O_8Er$	$C_{24}H_{32}K_2O_4$
Formula weight	1135.56	462.69
Temperature/K	100.00(10)	100.00(10)
Crystal system	Monoclinic	triclinic
Space group	*C*2/*c*	$P\bar{1}$
a/Å	10.8542(4)	9.8935(2)
b/Å	29.9272(10)	10.1172(2)
c/Å	16.3141(6)	12.0661(3)
α/°	90	87.623(2)
β/°	92.240(3)	88.304(2)
γ/°	90	75.410(2)
Volume/Å^3	5295.4(3)	1167.57(5)
Z	4	2
ρ_{calc}g/cm^3	1.424	1.316
μ/mm^{-1}	1.720	3.802
F(000)	2356.0	492.0
Crystal size/mm^3	0.323 × 0.266 × 0.098	0.307 × 0.105 × 0.069
Radiation	Mo K_α (λ = 0.71073)	Cu K_α (λ = 1.54184)
2Θ range for data collection/°	4.634 to 58.038	7.334 to 159.38
Index ranges	$-13 \le h \le 14$, $-40 \le k \le 32$, $-20 \le l \le 21$	$-11 \le h \le 12$, $-12 \le k \le 12$, $-13 \le l \le 15$
Reflections collected	26088	15870
Independent reflections	6329 [R_{int} = 0.0427, R_σ = 0.0352]	4970 [R_{int} = 0.0431, R_σ = 0.0442]
Data/restraints/parameters	6329/75/378	4970/45/341
Goodness-of-fit on F^2	1.114	1.067
Final R indexes [I>=2σ (I)]	R_1 = 0.0358, wR_2 = 0.0872	R_1 = 0.0477, wR_2 = 0.1330
Final R indexes [all data]	R_1 = 0.0439, wR_2 = 0.0903	R_1 = 0.0513, wR_2 = 0.1373
Largest diff. peak/hole / e Å^{-3}	0.85/-1.60	1.63/-0.51

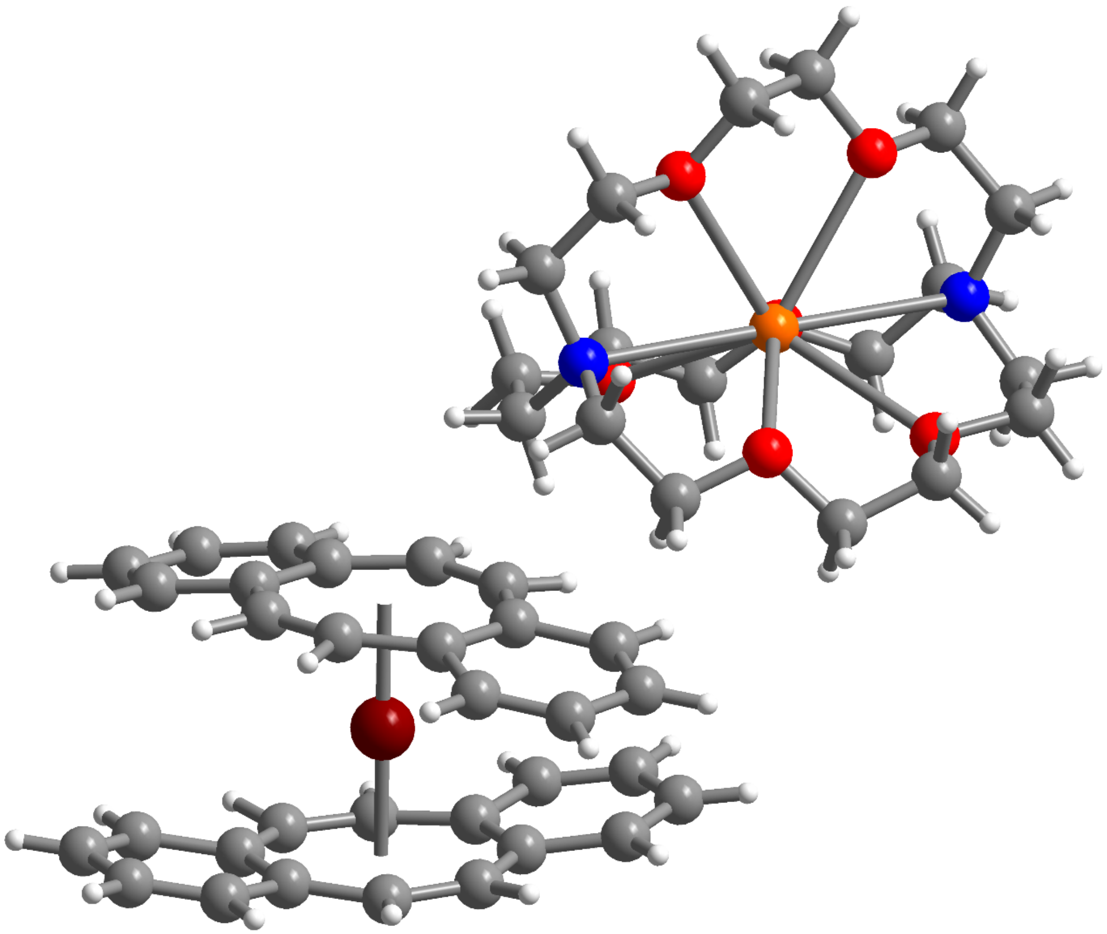

Figure S1. Structure of [K(crypt-222)][Er(dbCOT)$_2$], **1**. Maroon, orange, red, blue, gray, and white-gray spheres represent Er, K, O, N, C and H atoms, respectively. Solvent molecules (in the crystal lattice) have been omitted for clarity.

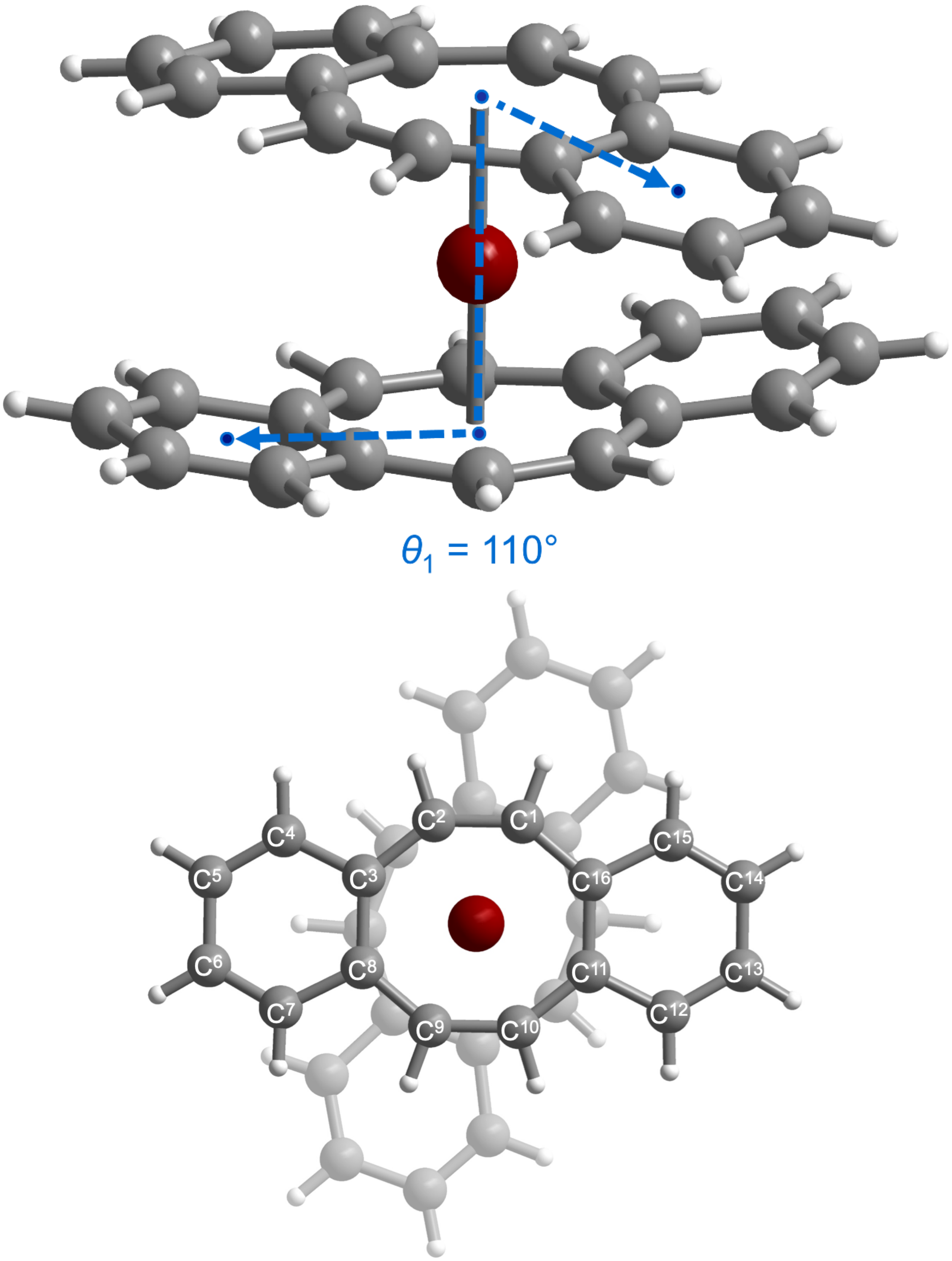

Figure S2. Structure of the $[Er(dbCOT)_2]^-$ anion in a crystal of $[K(crypt\text{-}222)][Er(dbCOT)_2]$, **1** (top). The dihedral angles (θ_1 = 110° and θ_2 = 70°) were calculated using the benzo and COT ring centroids. Arial perspective of **1** with corresponding atom labels (bottom). Maroon, gray, and white-gray spheres represent Er, C, and H atoms, respectively. One of the $(dbCOT)^{2-}$ ligands has been faded for clarity. The $[K(crypt\text{-}222)]^+$ counter cation and solvent molecules (in the crystal lattice) have been omitted for clarity.

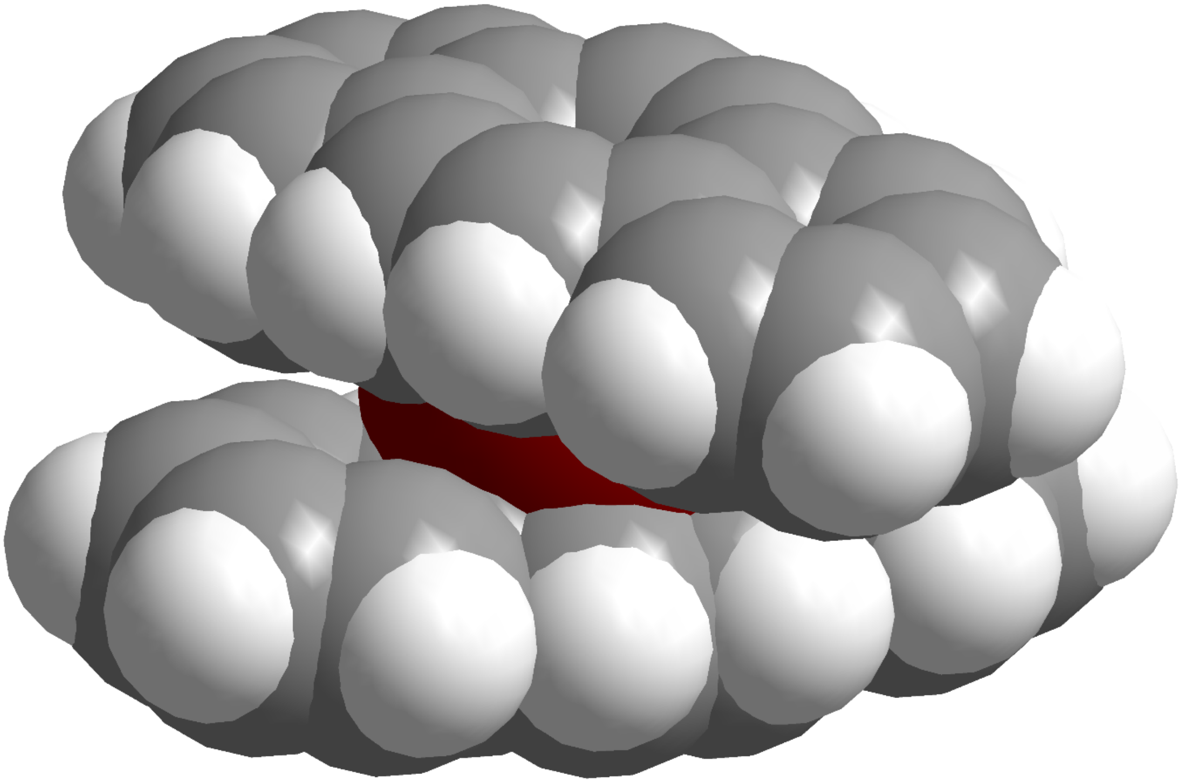

Figure S3. Space filling model of the $[Er(dbCOT)_2]^{-}$ anion in a crystal of $[K(crypt\text{-}222)][Er(dbCOT)_2]$, **1**. Maroon, gray, and white-gray spheres represent Er, C, and H atoms, respectively. The $[K(crypt\text{-}222)]^{+}$ counter cation and solvent molecules (in the crystal lattice) have been omitted for clarity.

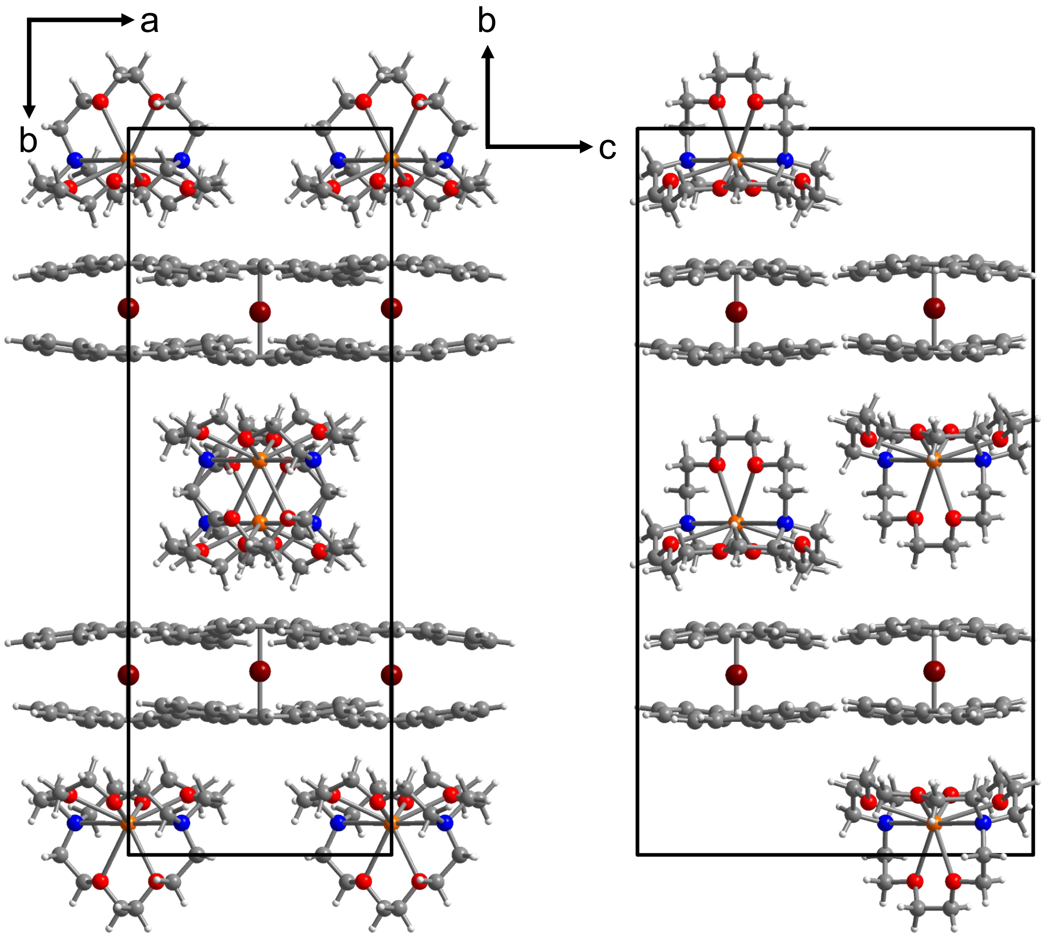

Figure S4. Unit cell of [K(crypt-222)][Er(dbCOT)$_2$], **1**. Maroon, orange, red, blue, gray, and white-gray spheres represent Er, K, O, N, C, and H atoms, respectively. Solvent molecules (in the crystal lattice) have been omitted for clarity.

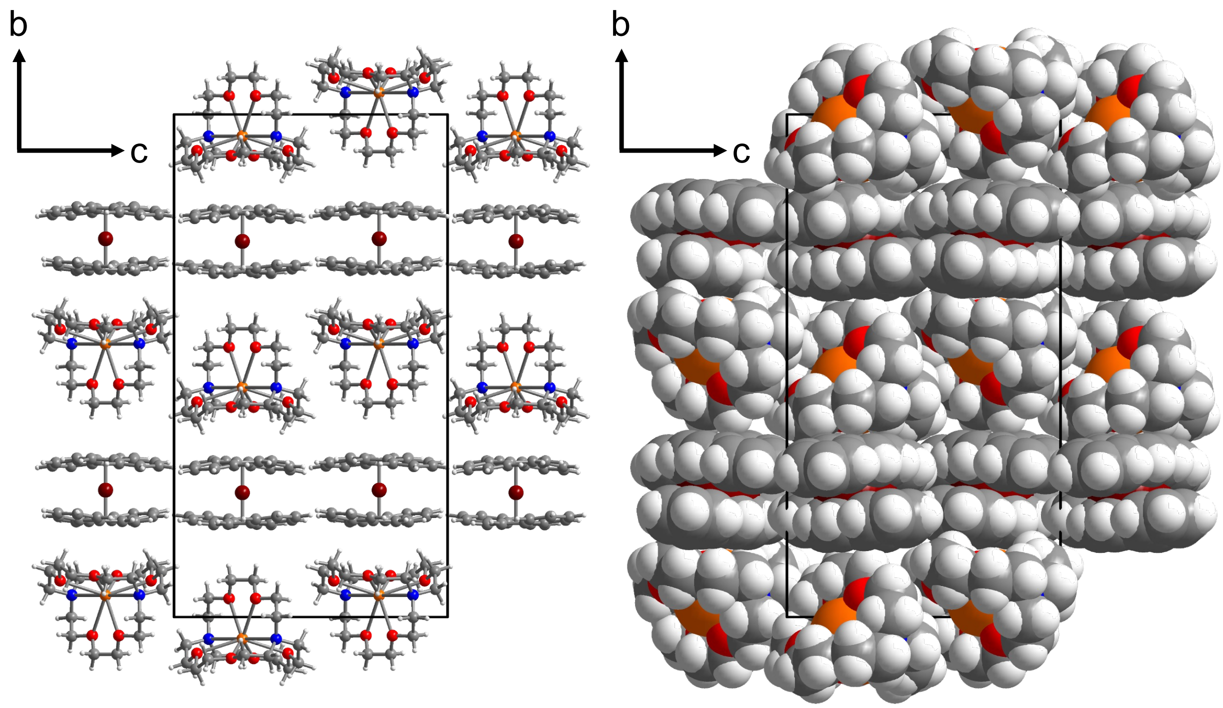

Figure S5. Crystal packing diagram of [K(crypt-222)][Er(dbCOT)$_2$], **1**, with the ball and stick (left) and space filling model (right) representations. Maroon, orange, red, blue, gray, and white-gray spheres represent Er, K, O, N, C, and H atoms, respectively. Solvent molecules (in the crystal lattice) have been omitted for clarity.

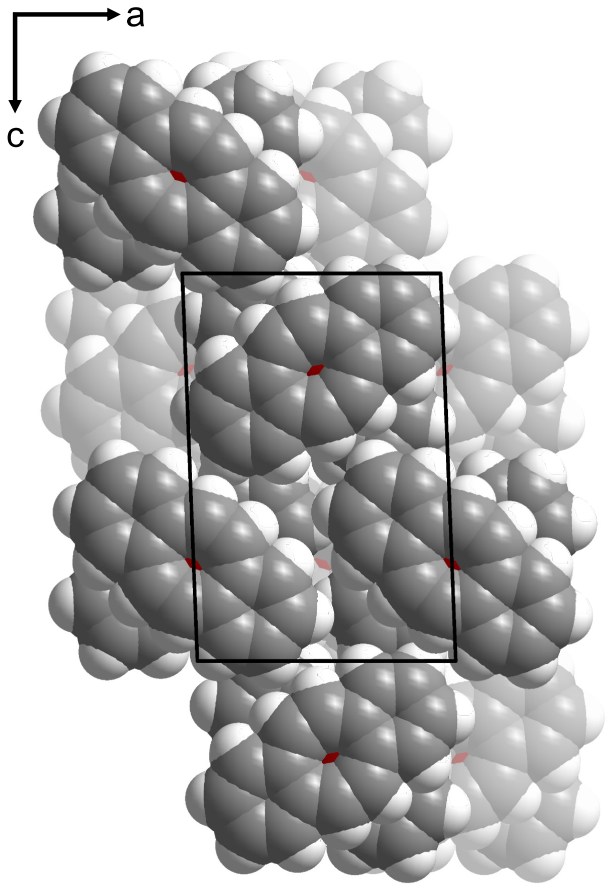

Figure S6. Crystal packing diagram of the $[Er(dbCOT)_2]^{-}$ anion in a crystal of [K(crypt-222)]$[Er(dbCOT)_2]$, **1**. Maroon, gray, and white-gray spheres represent Er, C, and H atoms, respectively. The $[K(crypt\text{-}222)]^{+}$ counter cation and solvent molecules (in the crystal lattice) have been omitted for clarity.

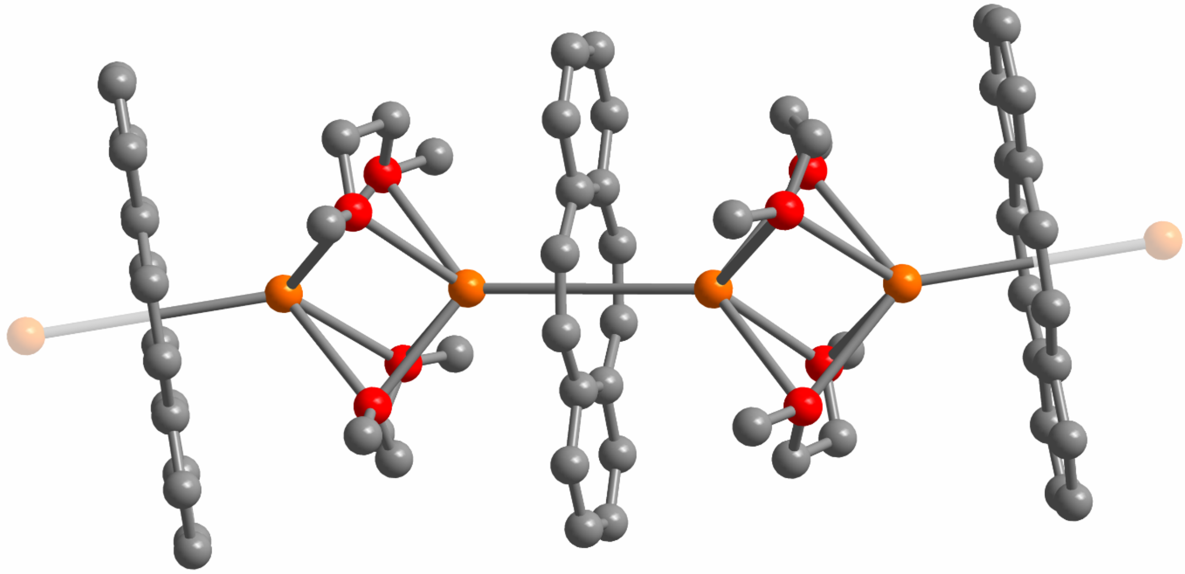

Figure S7. Polymeric structure of $[K(DME)]_2[dbCOT]_n$, **2**. Orange, red, and gray spheres represent K, O, and C, respectively.

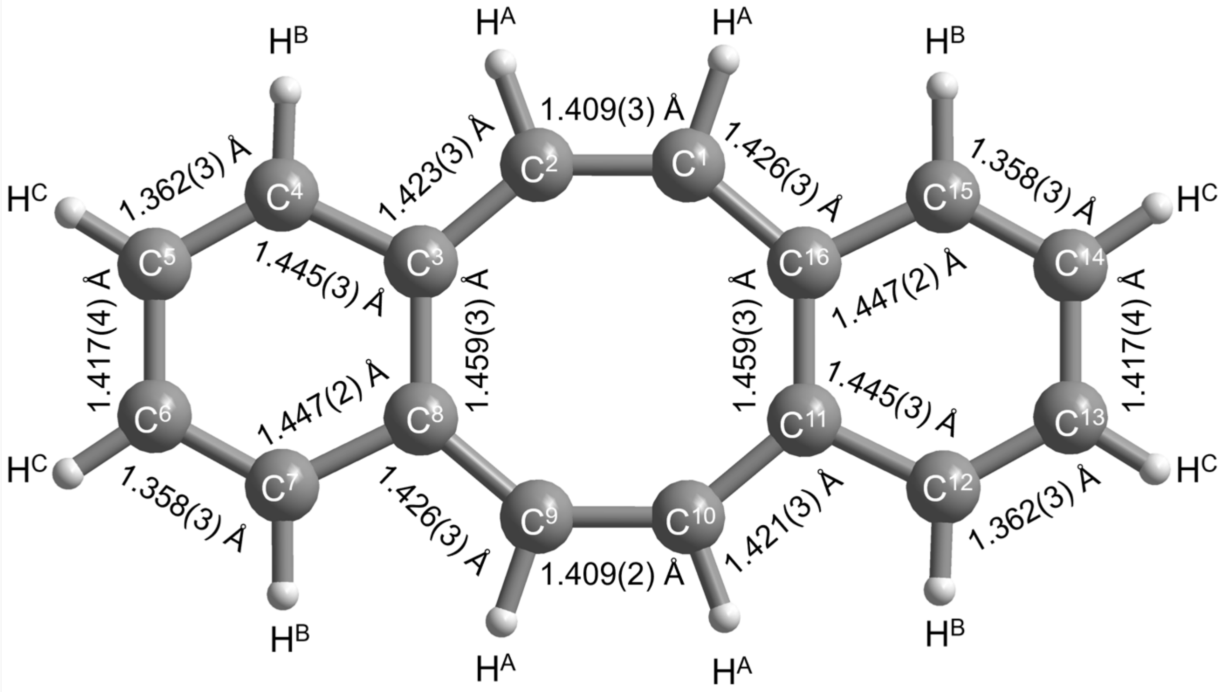

Figure S8. Structure of the $[dbCOT]^{2-}$ dianion in a crystal of $[K(DME)]_2[dbCOT]_n$, **2**. Arial perspective of **2** with corresponding atom labels. Gray and white-gray spheres represent C and H atoms, respectively. The capping $[K(DME)]_2^+$ counter cations and solvent molecules (ligated to K^+) have been omitted for clarity.

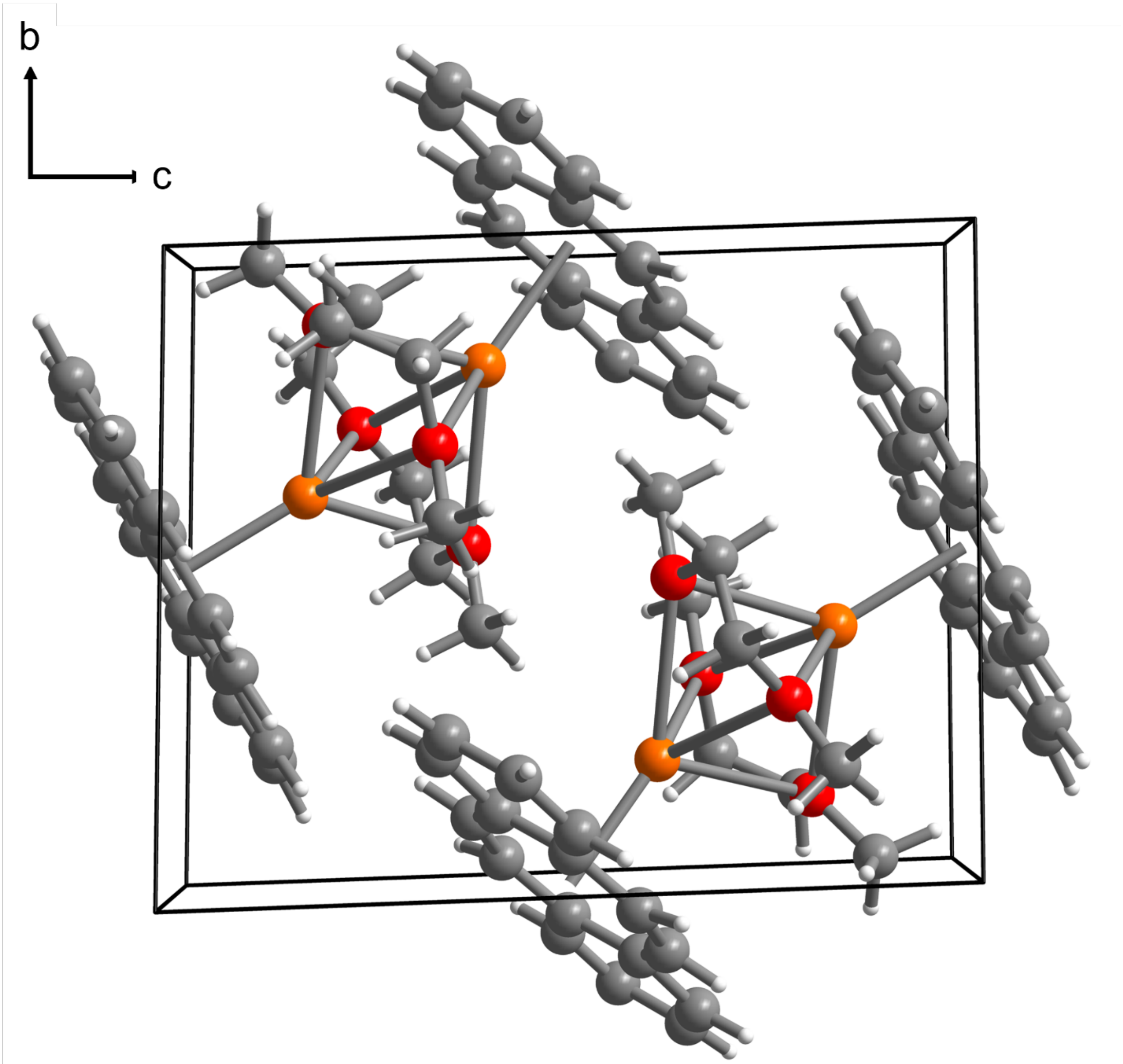

Figure S9. Unit cell of [K(DME)]$_2$[dbCOT]$_n$, **2**. Orange, red, gray, and white-gray spheres represent K, O, C, and H atoms, respectively.

Table S2. Metrical parameters for the C–C_{COT} distances of the cyclooctatetraenyl and benzo moieties in the crystal structure of [K(crypt-222)][Er(dbCOT)$_2$], **1**. Atom labels correspond to those given in Figure S2.

C_{COT}–C_{COT} Distances (Å)			
C1–C2	1.402(1)	C1'–C2'	1.411(1)
C2–C3	1.425(1)	C2'–C3'	1.418(1)
C3–C8	1.450(1)	C3'–C8'	1.441(1)
C8–C9	1.420(1)	C8'–C9'	1.434(1)
C9–C10	1.402(1)	C9'–C10'	1.411(1)
C10–C11	1.425(1)	C10'–C11'	1.418(1)
C11–C16	1.450(1)	C11'–C16'	1.441(1)
C16–C1	1.420(1)	C16'–C1'	1.434(1)
**C_{benzo}–C_{benzo} Distances (Å)**			
C3–C4	1.450(1)	C11–C12	1.450(1)
C4–C5	1.355(1)	C12–C13	1.355(1)
C5–C6	1.401(1)	C13–C14	1.401(1)
C6–C7	1.335(1)	C14–C15	1.335(1)
C7–C8	1.455(1)	C15–C16	1.455(1)
C8–C3	1.450(1)	C16–C11	1.450(1)
C3'–C4'	1.447(1)	C11'–C12'	1.447(1)
C4'–C5'	1.363(1)	C12'–C13'	1.363(1)
C5'–C6'	1.408(1)	C13'–C14'	1.408(1)
C6'–C7'	1.337(1)	C14'–C15'	1.337(1)
C7'–C8'	1.446(1)	C15'–C16'	1.446(1)
C8'–C3'	1.441(1)	C16'–C11'	1.441(1)

COT = cyclooctatetraenyl ring of the $dbCOT^{2-}$ ligands in **1**. Benzo = fused benzene rings of the $dbCOT^{2-}$ ligands in **1**.

Table S3. Metrical parameters for the Er–C_{COT} distances and angles in the crystal structure of [K(crypt-222)][Er(dbCOT)$_2$], **1**. Atom labels correspond to those given in Figure S2.

Er–C_{COT} Distances (Å)			
Er–C1	2.581(1)	Er–C1'	2.577(1)
Er–C2	2.568(1)	Er–C2'	2.587(1)
Er–C3	2.646(1)	Er–C3'	2.642(1)
Er–C8	2.639(1)	Er–C8'	2.648(1)
Er–C9	2.581(1)	Er–C9'	2.577(1)
Er–C10	2.568(1)	Er–C10'	2.587(1)
Er–C11	2.646(1)	Er–C11'	2.642(1)
Er–C16	2.639(1)	Er–C16'	2.648(1)
**Cnt Distances (Å)**			
Er–Cnt	1.837		
Er–Cnt	1.832		
**Cnt Angles (°)**			
Cnt–Er–Cnt	180.0		
θ_1	109.9		
θ_2	70.1		

COT = cyclooctatetraenyl ring of the $dbCOT^{2-}$ ligands in **1**. Cnt = COT ring centroid. The dihedral angles (θ_1 = 110° and θ_2 = 70°) were calculated using the benzo and COT ring centroids (see Figure S2).

2 NMR Spectroscopy

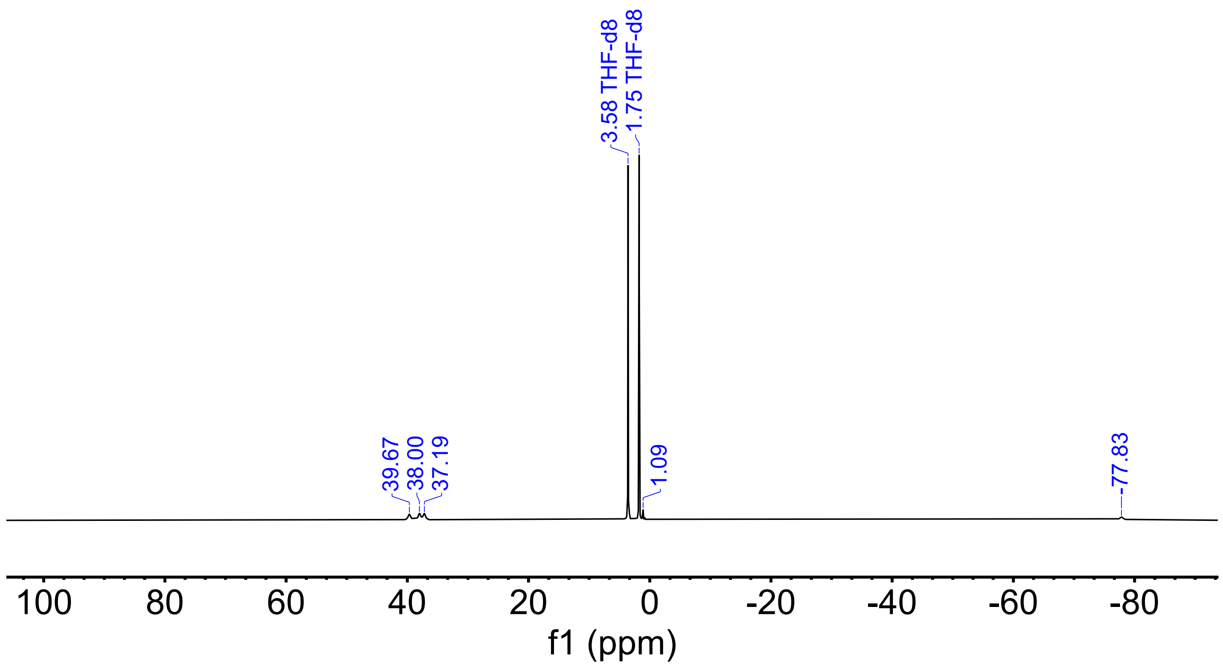

Figure S10. ^{1}H NMR spectrum of [K(crypt-222)][Er(dbCOT)$_2$], **1**, (500 MHz, THF-d_8, 25 °C): δ 39.67 (br), 38.00 (br), 37.19 (br), 1.09 (br), –77.83 (br).

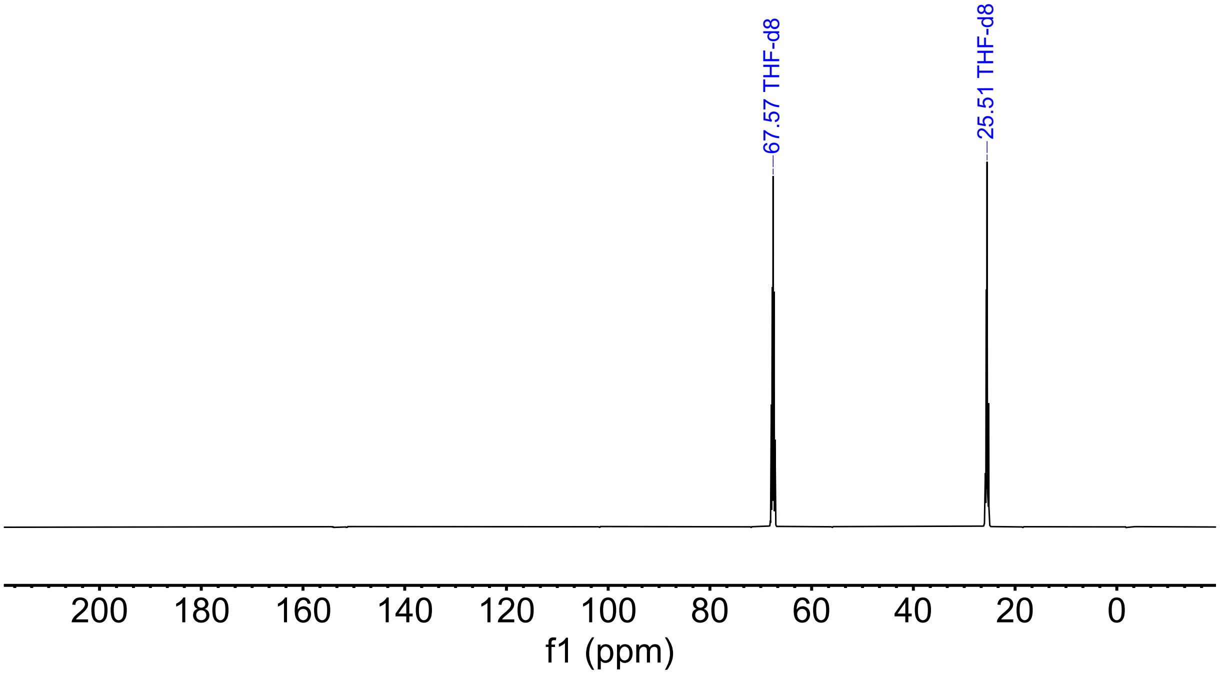

Figure S11. ^{13}C NMR spectrum of [K(crypt-222)][Er(dbCOT)$_2$], **1**, (126 MHz, THF-*d*$_8$, 25 °C). Owing to the paramagnetic nature of **1**, no signals were observed in the ^{13}C NMR spectrum.

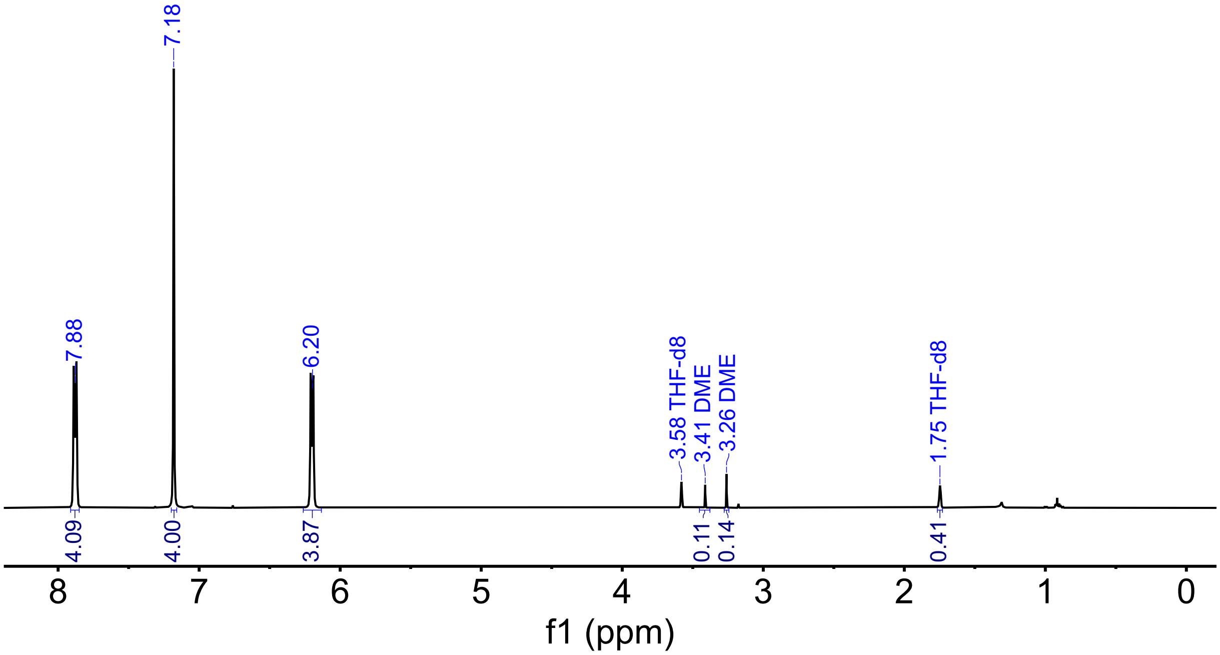

Figure S12. ^{1}H NMR spectrum of $[K(DME)]_2[dbCOT]_n$, **2**, (500 MHz, THF-d_8, 25 °C): δ 7.89-7.87 (AA'BB', 4H, benzo-H^C), 7.81 (s, 4H, COT-H^A), 6.21-6.19, (AA'BB', 4H, benzo-H^B). Atom labels correspond to those given in Figure S8.

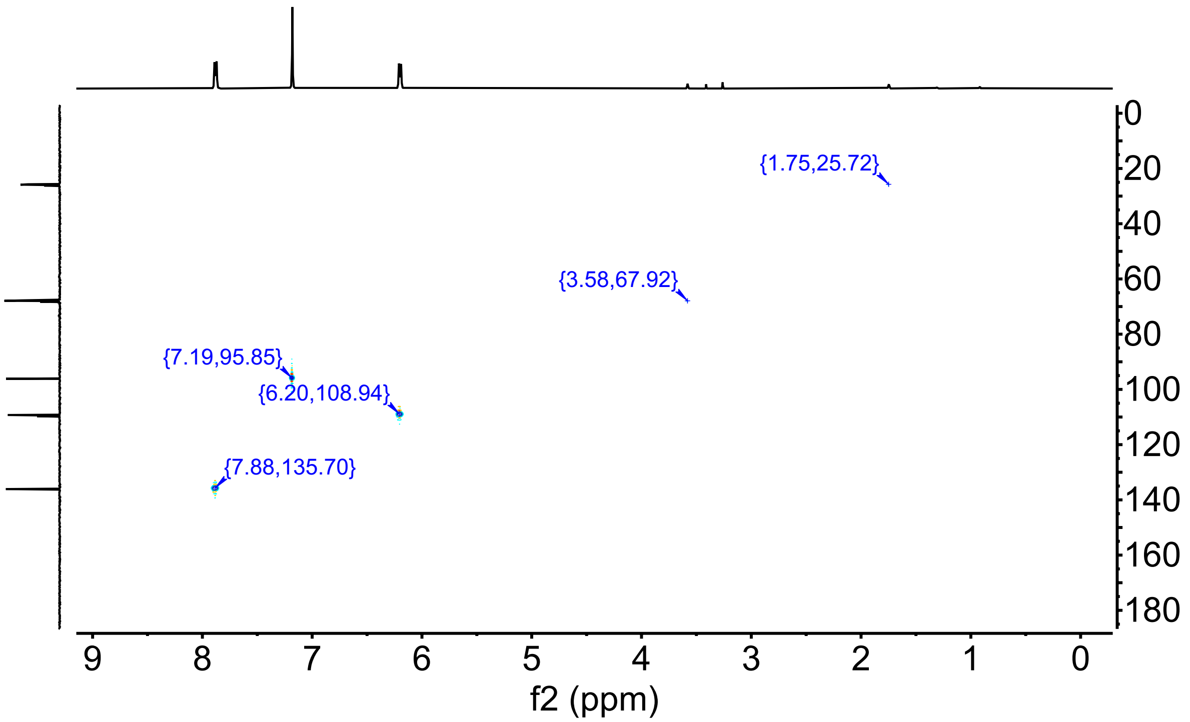

Figure S13. ^1H–^1H gCOSY spectrum of $[K(DME)]_2[dbCOT]_n$, **2**, (500 MHz, THF-d_8, 25 °C).

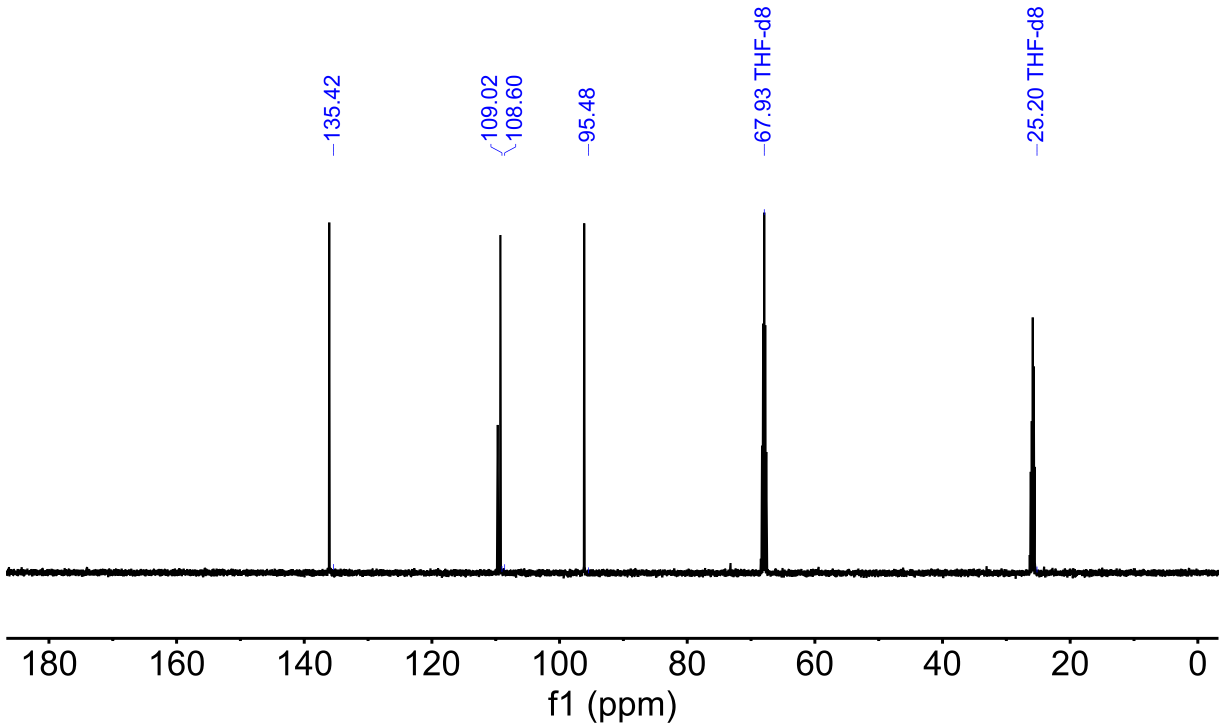

Figure S14. ^{13}C NMR spectrum of $[K(DME)]_2[dbCOT]_n$, **2**, (126 MHz, THF-d_8, 25 °C): δ 135.42 (benzo-$C^{4,7,12,15}$), 109.02 (COT-$C^{3,8,11,16}$), 108.60 (benzo-$C^{5,6,13,14}$), 95.48 (COT-$C^{1,2,9,10}$). Atom labels correspond to those given in Figure S8.

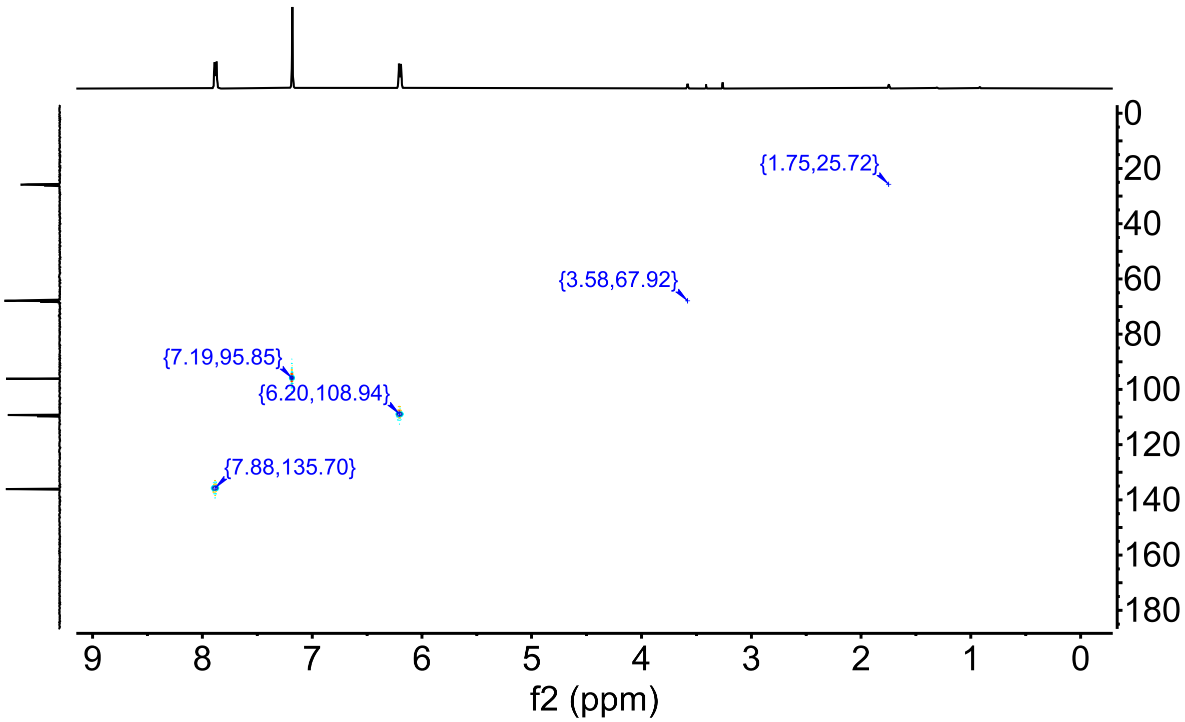

Figure S15. ^{1}H–^{13}C gHSQCAD spectrum of $[K(DME)]_2[dbCOT]_n$, **2**, (500 MHz, THF-d_8, 25 °C).

3 IR Spectroscopy

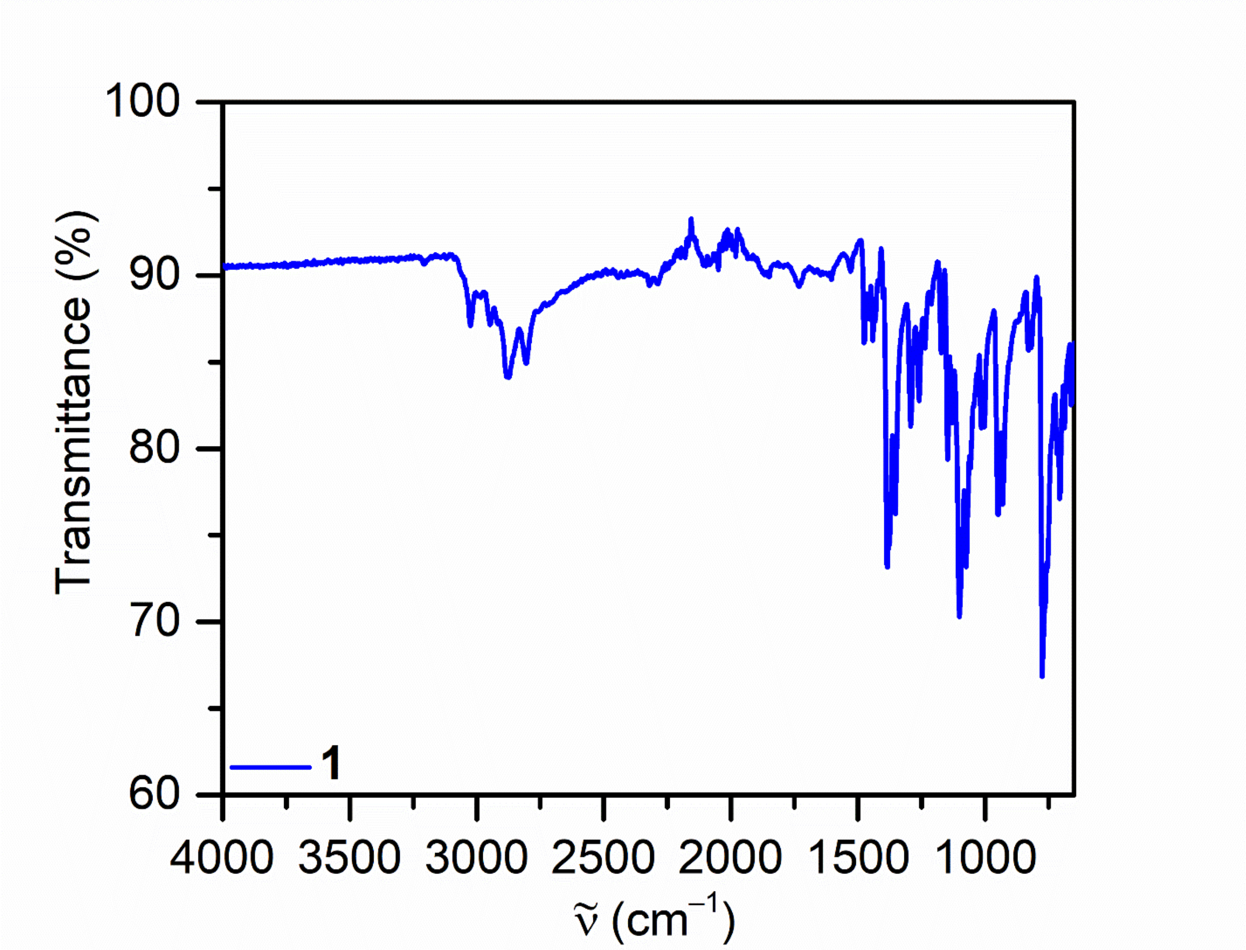

Figure S16. FTIR spectrum of [K(crypt-222)][Er(dbCOT)$_2$], **1**, measured on crushed crystalline solids in a nitrogen atmosphere.

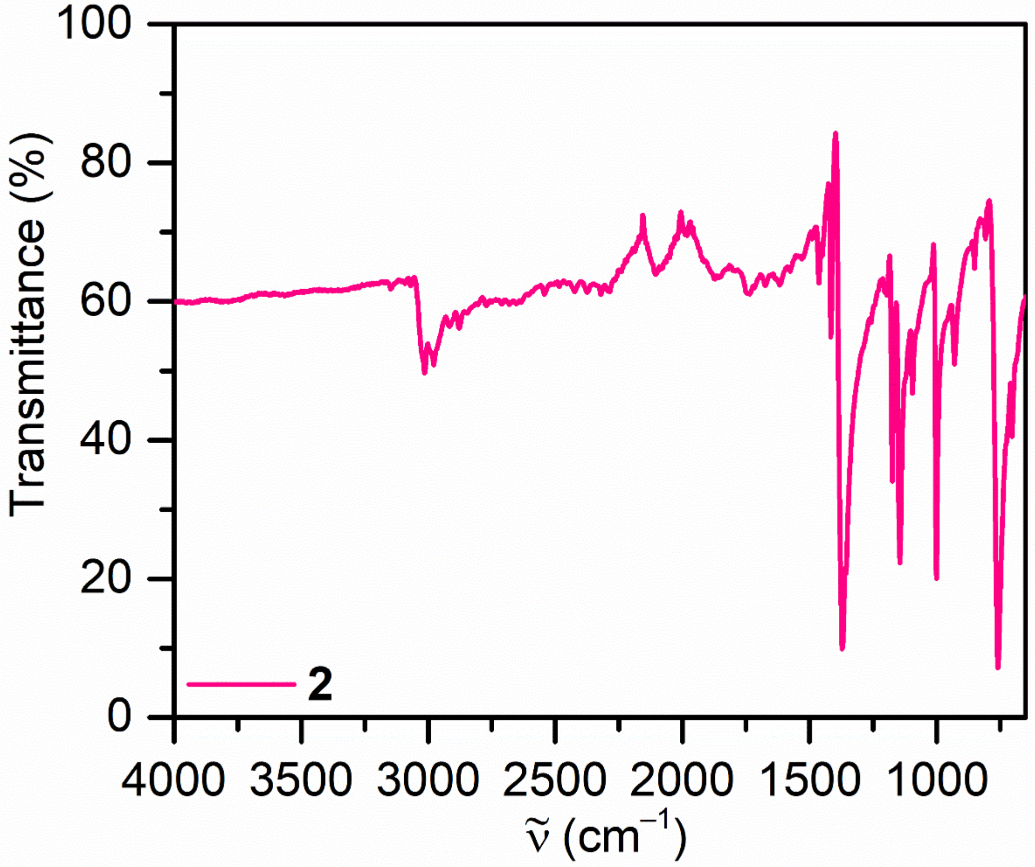

Figure S17. FTIR spectrum of $[K(DME)]_2[dbCOT]_n$, **2**, measured on crushed crystalline solids in a nitrogen atmosphere.

4 Electrochemistry

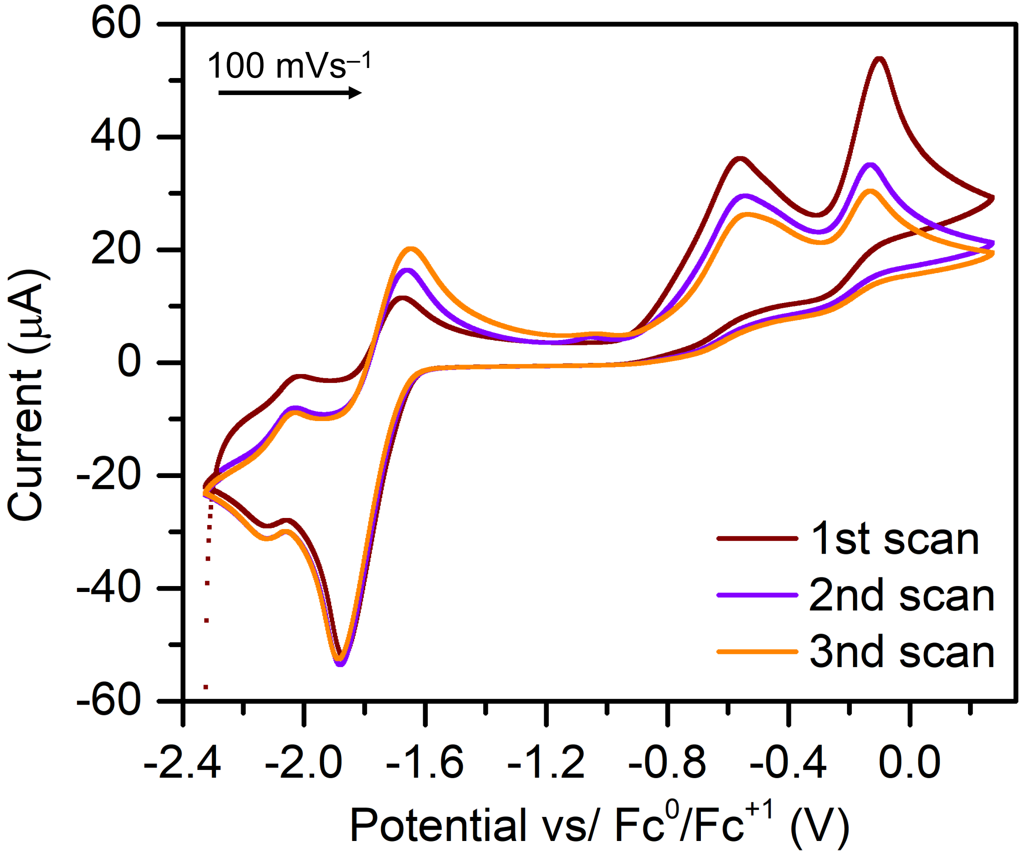

Figure S18. Cyclic voltammogram of [K(crypt-222)][Er(dbCOT)$_2$], **1** (3 mM), measured in a 250 mM [^{n}Bu$_4$N][PF$_6$] THF solution at a scan rate of 100 mVs^{-1}. Brown, purple, and orange lines represent the 1st, 2nd, and 3rd cycles, respectively. Voltammogram features four redox events: two quasi-reversible features at –2.050 ± 0.108 V, –1.741 ± 0.108 V, and two irreversible features –0.526 ± 0.108 and –0.113 ± 0.108 V.

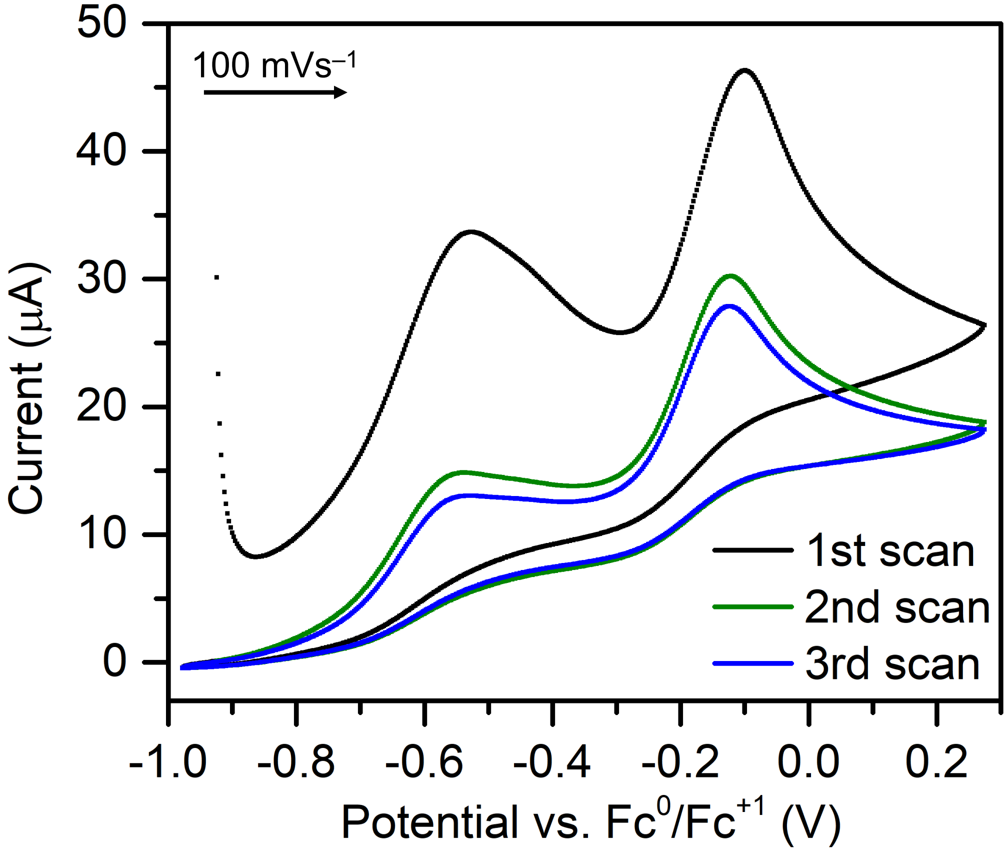

Figure S19. Cyclic voltammogram of [K(crypt-222)][Er(dbCOT)$_2$], **1** (3 mM), measured in a 250 mM [^{n}Bu$_4$N][PF$_6$] THF solution at a scan rate of 100 mVs^{-1}. Black, green, and blue lines represent the 1st, 2nd, and 3rd cycles, respectively. Magnifications of the quasi-reversible feature at –0.5261 ± 0.108 V and –0.1131 ± 0.108 V.

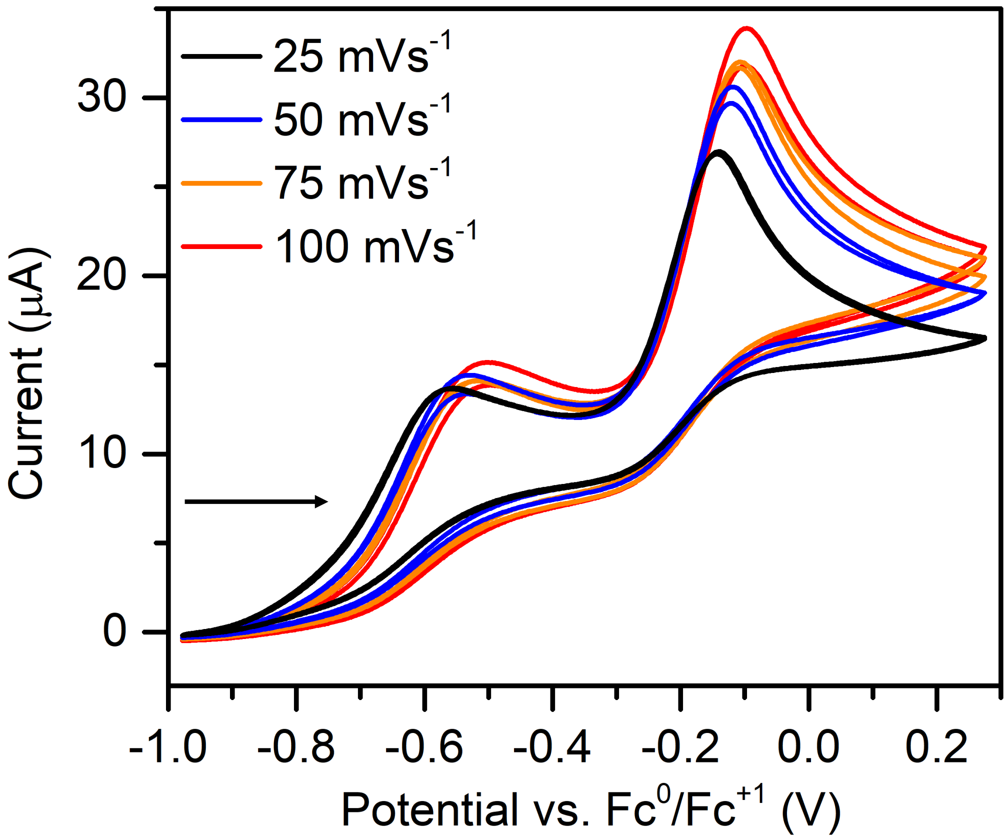

Figure S20. Cyclic voltammogram of [K(crypt-222)][Er(dbCOT)$_2$], **1** (3 mM), measured in a 250 mM [^{n}Bu$_4$N][PF$_6$] THF solution at scan rates between 25 and 100 mVs^{-1}, in 25 mVs^{-1} increments. Black, blue, orange, and red lines represent 25 mVs^{-1}, 50 mVs^{-1}, 75 mVs^{-1}, and 100 mVs^{-1}, respectively. Magnifications of the irreversible feature at –0.526 ± 0.108 V and –0.113 ± 0.108 V.

5 Magnetic Data

$$\tau^{-1} = CT^{n} + \tau_{\mathrm{QTM}}^{-1} \quad \textbf{(S1)}$$

$$\tau^{-1} = \tau_0^{-1} \exp\left(-\frac{U_{\mathrm{eff}}}{\mathrm{k}T}\right) + \tau_{\mathrm{QTM}}^{-1} \quad \textbf{(S2)}$$

$$\tau^{-1} = \tau_0^{-1} \exp\left(-\frac{U_{\mathrm{eff}}}{\mathrm{k}T}\right) + \mathrm{C}T^{n} \quad \textbf{(S3)}$$

Table S4. Single-molecule magnet behavior of homoleptic, mononuclear cyclooctatetraenyl-based Er complexes.

	**[K(18-c-6)] [Er(COT)$_2$]**			**[K(DME)$_2$] [Er(COT^{TBS2})$_2$]**	**[K(18-c-6)] [Er(hdcCOT)$_2$]**
**Dc Field (T)**	0	0	0	0	0
**U_{eff} (cm^{-1})**	147(1)	198.2(1)	142	187(1)	132(7)
**τ_0 (s)**	8.3(6) x 10^{-8}	3.7 x 10^{-9}	1.2 x 10^{-7}	4.0 x 10^{-8}	2.0 x 10^{-8}
**C (s^{-1} K^{-n})**	–	–	–	–	–
n	–	–	–	–	–
**τ_{QTM} (s)**	–	–	–	–	17(1)
**T_{hys} (K)**	10	12	12	8	10
**Sweep Rate (Oe s^{-1})**	7.8	35	60	22	200
**Er···Er (Å)**	7.240	7.250	7.240	10.513	10.543
**Er–Cnt (Å)**	1.884<br>1.848	1.85<br>1.89	1.848<br>1.882	1.881<br>1.886	1.858<br>1.919
**Cnt–Er–Cnt (°)**	177.2	178.0	175.19	176.5	174.8
**Ref**	1	2	7	3	4

	**[K(18-c-6)(THF)$_2$] [Er(COT^{TBS2})$_2$]**	**[K(crypt-222)] [Er(COT^{TBS2})$_2$]**		**[K(crypt-222)] [Er(dbCOT)$_2$]**	
**Dc Field (T)**	0	0	0	1000	1000
**U_{eff} (cm^{-1})**	119(7)	115(6)	–	114(2)	90.29
**τ_0 (s)**	2.0 x 10^{-8}	6.3 x 10^{-8}	–	2.1(1) x 10^{-7}	2.94 x 10^{-7}
**C (s^{-1} K^{-n})**	–	–	9.49(1.3) x 10^{-3}	2.5(1) x 10^{-3}	–
n	–	–	4.44(9)	4.7(2)	–
**τ_{QTM} (s)**	13(1)	65(2)	1.98(1.0) x 10^{-3}	–	–
**T_{hys} (K)**	9	11		3*	
**Sweep Rate (Oe s^{-1})**	200	200		100	
**Er···Er (Å)**	9.282	16.706		9.621	
**Er–Cnt (Å)**	1.888<br>1.889	1.903<br>1.904		1.832<br>1.837	
**Cnt–Er–Cnt (°)**	174.3	173.6		180.0	
**Ref**	4	4		*this work*	

*Denotes a closed butterfly-type hysteresis loop with no remnant magnetization (M_R) at 0 T. 18-c-6 = 18-crown-6, crypt-222 = 2.2.2-cryptand, COT = cyclooctatetraenyl, COT″ = 1,4-bis(trimethylsilyl)cyclooctatetraenyl, COT^{TBS2} = 1,4-bis(*tert*-butyl-dimethylsilyl)cyclooctatetraenyl, dbCOT = dibenzocyclooctatetraenyl, DME = dimethoxyethane, hdcCOT = hexahydrodicyclopentacyclooctatetraenyl.

Table S4. Single-molecule magnet behavior of homoleptic, mononuclear cyclooctatetraenyl-based Er complexes – continued.

	**[K(DME)$_2$] [Er(COT^{TMS})$_2$]**	**[K(DME)$_2$] [Er(COT^{TMS2})$_2$]**	**[K(18-c-6)(THF)$_2$] [Er(COT^{TMS3})$_2$]**
**Dc Field (T)**	0	0	0
U_{eff} **(cm^{-1})**	145	134	151
τ_0 **(s)**	1.11(5) x 10^{-7}	3.14(4) x 10^{-8}	2.04(2) x 10^{-9}
C **(s^{-1} K^{-n})**	2.67(5) x 10^{-14}	3.56(4) x 10^{-13}	3.55(6) x 10^{-12}
n	12.09(6)	11.61(4)	12.11(8)
τ_{QTM} **(s)**	–	–	–
T_{hys} **(K)**	12	10	9
**Sweep Rate (Oe s^{-1})**	200	200	200
**Er···Er (Å)**	7.074	9.962	12.181
**Er–Cnt (Å)**	1.832<br>1.890	1.833<br>1.899	1.912<br>1.929
**Cnt–Er–Cnt (°)**	179.8	174.8	175.5
**Ref**	6	6	6
	**[Cp*$_2$Co] [Er(COT)$_2$]**	**[K(crypt-222)] [Er(COT)$_2$]**	**[K(18-c-6)] [Er(hdcCOT)$_2$]**
**Dc Field (T)**	0	0	0
U_{eff} **(cm^{-1})**	145	133	147.(7)
τ_0 **(s)**	2.5 x 10^{-7}	4.1 x 10^{-7}	3.1 x 10^{-8}
C **(s^{-1} K^{-n})**	–	–	–
n	–	–	–
τ_{QTM} **(s)**	–		–
T_{hys} **(K)**	12	8*	6
**Sweep Rate (Oe s^{-1})**	60	60	10
**Er···Er (Å)**	9.419	6.784	10.356
**Er–Cnt (Å)**	1.859<br>1.879	1.861<br>1.861	1.838<br>1.858
**Cnt–Er–Cnt (°)**	179.9	180.0	178.1
**Ref**	7	7	5

Denotes a closed butterfly-type hysteresis loop with no remnant magnetization (M_R) at 0 T. 18-c-6 = 18-crown-6, Cp = 1,2,3,4,5-pentalmethylcyclopentadienyl, crypt-222 = 2.2.2-cryptand, COT = cyclooctatetraenyl, COT″ = 1,4-bis(trimethylsilyl)cyclooctatetraenyl, COT^{TBS2} = 1,4-bis(*tert*-butyl-dimethylsilyl)cyclooctatetraenyl, dbCOT = dibenzocyclooctatetraenyl, DME = dimethoxyethane, hdcCOT = hexahydrodicyclopentacyclooctatetraenyl.

Table S5. Single-molecule magnet behavior of heteroleptic, mononuclear cyclooctatetraenyl-based Er complexes.

	(COT)Er(CNT)		(COT)Er(Cp*)	
**Dc Field (T)**	0	2000	0	0
**U_{eff} (cm^{-1})**	251(1)	261(1)	224.9	136.9
**τ_0 (s)**	$1.3(2) \times 10^{-10}$	$8.8(3) \times 10^{-9}$	8.17×10^{-11}	3.13×10^{-9}
**C (s^{-1} K^{-n})**	–	–	–	–
n	–	–	–	–
**τ_{QTM} (s)**	0.1	–	–	–
**T_{hys} (K)**	10*		5*	
**Sweep Rate ($Oe\ s^{-1}$)**	700		9.2	
**Er···Er (Å)**	7.128(1)		7.058	
**Er–Cnt (Å)**	1.673		1.66	
**Cnt–Er–Cnt (°)**	177.5		171.2	
**Ref**	8		9	
	**[(COT)Er(THF)$_3$][CNT]**		**(COT)Er((S)-PETA)**	
**Dc Field (T)**	0	2000	1000	1000
**U_{eff} (cm^{-1})**	67.2(1)	82.6(1)	–	34.5
**τ_0 (s)**	$2.54(4) \times 10^{-9}$	$4.21(1) \times 10^{-10}$	–	1.8×10^{-7}
**C (s^{-1} K^{-n})**	1214(6)	6.4×10^{-3}	4.6×10^{-3}	–
n	0.541(3)	5.56(2)	7.15	–
**τ_{QTM} (s)**	–	–	–	–
**T_{hys} (K)**	0		0	
**Sweep Rate ($Oe\ s^{-1}$)**	–		–	
**Er···Er (Å)**	10.837		11.226	
**Er–Cnt (Å)**	1.726		1.829	
**Cnt–Er–Cnt (°)**	–		–	
**Ref**	10		11	

Denotes a closed butterfly-type hysteresis loop with no remnant magnetization (M_R) at 0 T. CNT = cyclononatetraenyl, COT = cyclooctatetraenyl, Cp = 1,2,3,4,5-pentamethylcyclopentadienyl, DMPE = 1,2-bis(dimethylphosphino)ethane, (S)-PETA = (S,S)-N,N′bis(1-phenylethyl) pivalamidinate, py = pyridine, THF = tetrahydrofuran, Tp* = tris(3,5-dimethylpyrazolyl)borate.

Table S5. Single-molecule magnet behavior of selected heteroleptic, mononuclear cyclooctatetraenyl-based Er complexes – continued.

	(COT)Er(I)(py)$_2$	(COT)Er(I)(MeCN)$_2$	(COT)Er(I)(Tp*)	[(COT)Er(THF)$_4$][BPh$_4$]
**Dc Field (T)**	0	0	0	0
**U_{eff} (cm^{-1})**	102.9(3.1)	107.1(1.3)	133.6(2.2)	126.4
**τ_0 (s)**	9.6(2.7) x 10^{-10}	6.3(8) x 10^{-10}	9.0(1.4) x 10^{-10}	1.3 x 10^{-11}
**C (s^{-1} K^{-n})**	–	–	–	8.80
n	–	–	–	3.21
**τ_{QTM} (s)**	2.02(8) x 10^{-3}	1.50(2) x 10^{-3}	1.45(14) x 10^{-2}	–
**T_{hys} (K)**	0	0	0	0
**Sweep Rate (Oe s^{-1})**	–	–	–	0
**Er···Er (Å)**	7.261	6.753	9.528	8.201
**Er–Cnt (Å)**	1.770	1.749	1.836	1.850
**Cnt–Er–Cnt (°)**	–	–	–	–
**Ref**	12	12	12	13

	(COT)Er(DMPE)I	(COT)Er(I)(THF)$_2$	(COT^{TiPS2})Er(I)(THF)$_2$
**Dc Field (T)**	0	0	0
**U_{eff} (cm^{-1})**	75.6(15.2)	95.6(9)	82(2)
**τ_0 (s)**	6.9(4.3) x 10^{-8}	9.2(1.0) x 10^{-10}	4.6(7) x 10^{-9}
**C (s^{-1} K^{-n})**	–	–	2.5 x 10^{-5}
n	–	–	7.5(3)
**τ_{QTM} (s)**	–	2.27(4) x 10^{-3}	9.9(1) x 10^{-3}
**T_{hys} (K)**	0	0	2*
**Sweep Rate (Oe s^{-1})**	10.1	–	–
**Er···Er (Å)**	7.130	9.771	9.058
**Er–Cnt (Å)**	1.760	1.771	1.790
**Cnt–Er–Cnt (°)**	–	–	–
**Ref**	14	12	15

Denotes a closed butterfly-type hysteresis loop with no remnant magnetization (M_R) at 0 T. CNT = cyclononatetraenyl, COT = cyclooctatetraenyl, COT^{TiPS2} = 1,4-bis(triisopropyl)cyclooctatetraenyl, Cp = 1,2,3,4,5-pentamethylcyclopentadienyl, DMPE = 1,2-bis(dimethylphosphino)ethane, (S)-PETA = (S,S)-N,N′bis(1-phenylethyl)pivalamidinate, py = pyridine, THF = tetrahydrofuran, Tp* = tris(3,5-dimethylpyrazolyl)borate.

Table S6. Single-molecule magnet behavior of mononuclear cyclooctatetraenyl-based Er complexes containing heterocycles.

	**(COT)Er(DSP)**	**[K(crypt-222)] [(COT)Er(Cp^{Ge})]**	**(COT)Er($C_5H_5BNEt_2$)**	
**Dc Field (T)**	0	0	0	2000
**U_{eff} (cm^{-1})**	249(2)	120(1)	174	219
**τ_0 (s)**	1.6(3) x 10^{-11}	$10^{-9.02(4)}$	9.2 x 10^{-10}	6.0 x 10^{-11}
**C (s^{-1} K^{-n})**	–	$10^{-1.6(4)}$	–	–
**n**	–	3.4(4)	–	–
**τ_{QTM} (s)**	–	$10^{-1.7(1)}$	–	–
**T_{hys} (K)**	10	10	2*	
**Sweep Rate (Oe s^{-1})**	200	20	19	
**Er···Er (Å)**	7.832	8.843	6.3	
**Er–Cnt (Å)**	1.686	1.761	1.680	
**Cnt–Er–Cnt (°)**	170.8	175.4	170.7	
**Ref**	16	17	18	
	**Li(THF)[(COT^{TIPS})Er(PbI^{TBDMS,Ph})]**		**[Li(12-c-4)$_2$](COT^{TIPS})Er(PbI^{TBDMS,Ph})**	
**Dc Field (T)**	0		0	
**U_{eff} (cm^{-1})**	100(5)		42	
**τ_0 (s)**	4.4(2.4) x 10^{-9}		2.3(2.1) x 10^{-8}	
**C (s^{-1} K^{-n})**	–		–	
**n**	–		–	
**τ_{QTM} (s)**	1.7(0.02) x 10^{-2}		2.5(0.03) x 10^{-4}	
**T_{hys} (K)**	5		4	
**Sweep Rate (Oe s^{-1})**	200		200	
**Er···Er (Å)**	13.088		10.902	
**Er–Cnt (Å)**	1.980		1.780	
**Cnt–Er–Cnt (°)**	161.3		168.3	
**Ref**	19		19	

*Denotes a closed butterfly-type hysteresis loop with no remnant magnetization (M_R) at 0 T. 12-c-4 = 12-crown-4, crypt-222 = 2.2.2-cryptand, COT = cyclooctatetraenyl; DSP = 3,4-dimethyl-2,5-bis(trimethylsilyl)phospholyl, Cp^{Ge} = 3,4-(dimethyl-2,5-bis(trimethylsilyl)germolyl, PbI^{TBDMS,Ph} = 1,4-bis(*tert*-butyl-dimethylsilyl)-2,3-diphenylplumbolyl, THF = tetrahydrofuran.

Table S6. Single-molecule magnet behavior of mononuclear cyclooctatetraenyl-based Er complexes containing heterocycles – continued.

	[Li(DME)$_3$ [(COT)Er(^{t}Bu-^{N}benzoborole)]	(COT)Er(C_5H_5BH)	(COT)Er(C_5H_5BMe)
**Dc Field (T)**	0	0	0
U_{eff} **(cm^{-1})**	109	259	300
τ_0 **(s)**	9.16 x 10^{-12}	5.3 x10^{-12}	5.5 x 10^{-12}
C **(s^{-1} K^{-n})**	5.7 x 10^{-6}	–	–
n	912	–	–
τ_{QTM} **(s)**	8.7 x10^{-3}	–	–
T_{hys} **(K)**	2*	8*	6*
**Sweep Rate (Oe s^{-1})**	–	19	19
**Er···Er (Å)**	7.993	6.1	6.8
**Er–Cnt (Å)**	1.732	1.678	1.674
**Cnt–Er–Cnt (°)**	169.3	169.4	174.5
**Ref**	20	18	18

*Denotes a closed butterfly-type hysteresis loop with no remnant magnetization (M_R) at 0 T. 12-c-4 = 12-crown-4, crypt-222 = 2.2.2-cryptand, COT = cyclooctatetraenyl; DSP = 3,4-dimethyl-2,5-bis(trimethylsilyl)phospholyl, Cp^{Ge} = 3,4-(dimethyl-2,5-bis(trimethylsilyl)germolyl, $Pbl^{TBDMS,Ph}$ = 1,4-bis(*tert*-butyl-dimethylsilyl)-2,3-diphenylplumbolyl, THF = tetrahydrofuran.

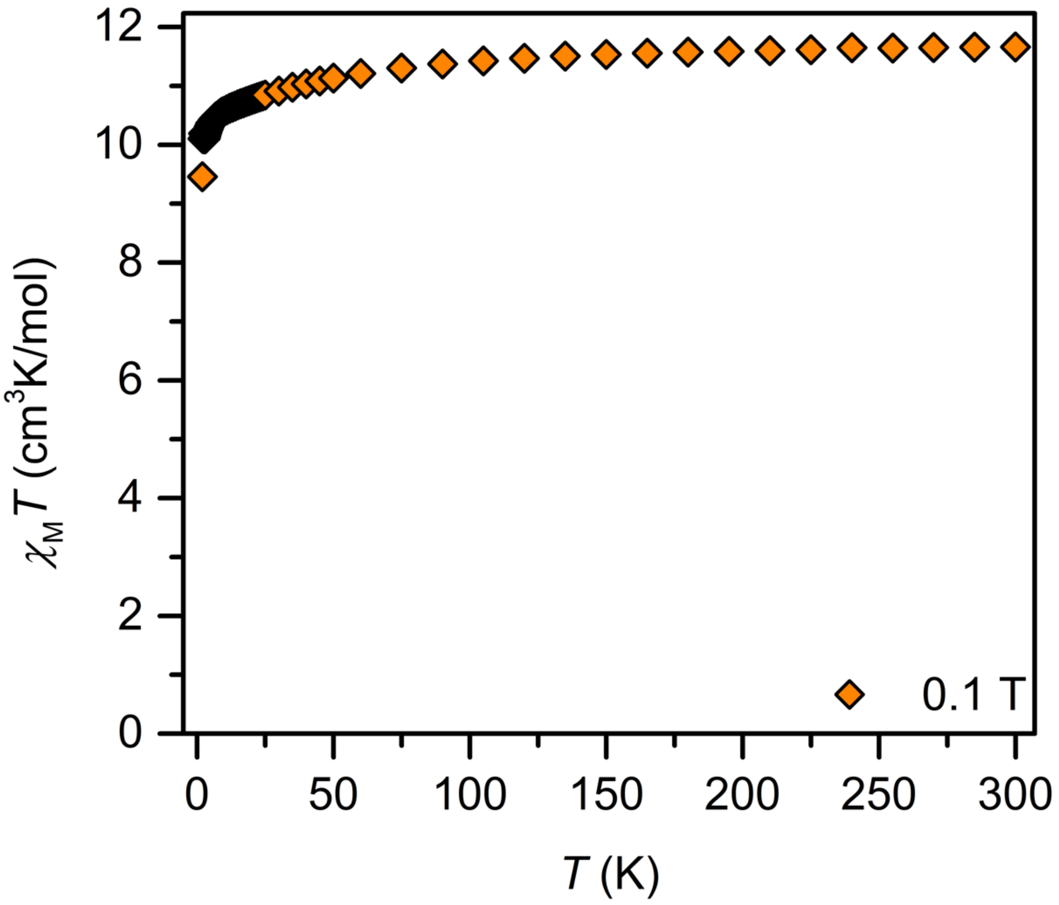

Figure S21. Variable-temperature dc magnetic susceptibility data of [K(crypt-222)][Er(dbCOT)$_2$], **1**, collected under a 0.1 T applied dc field.

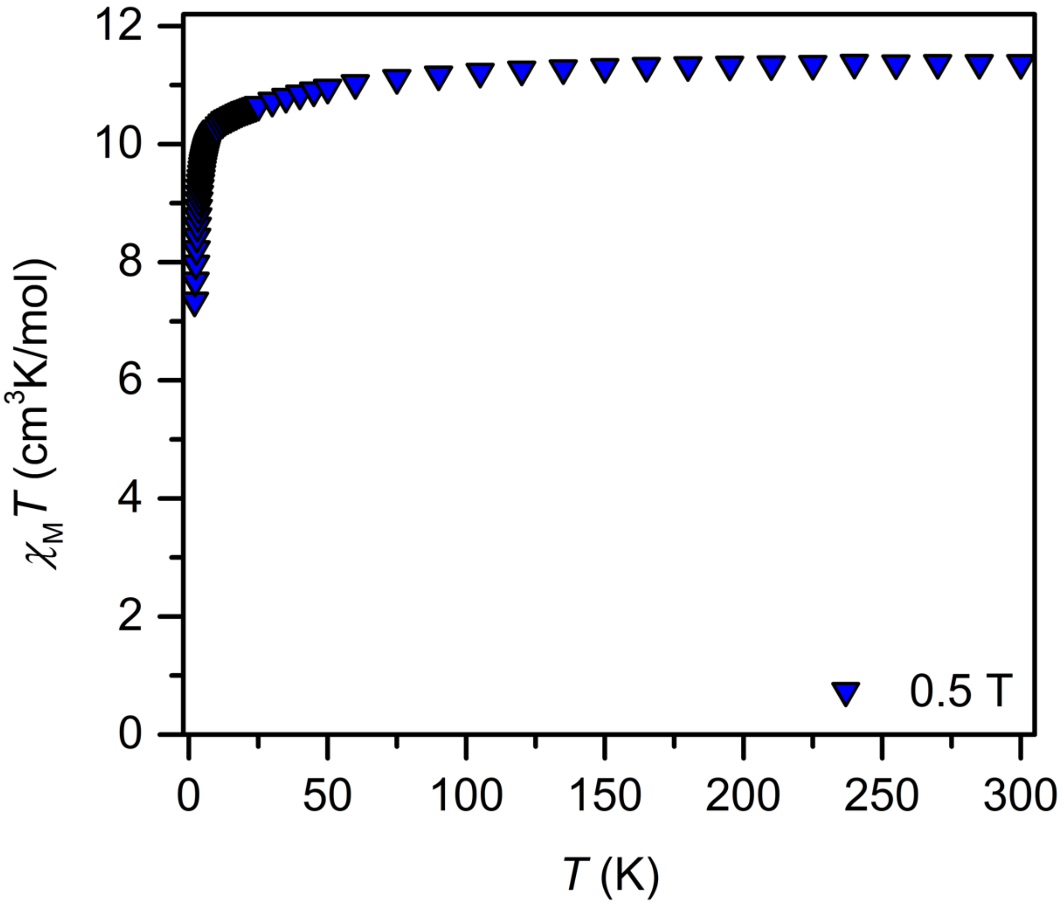

Figure S22. Variable-temperature dc magnetic susceptibility data of [K(crypt-222)][Er(dbCOT)$_2$], **1**, collected under a 0.5 T applied dc field.

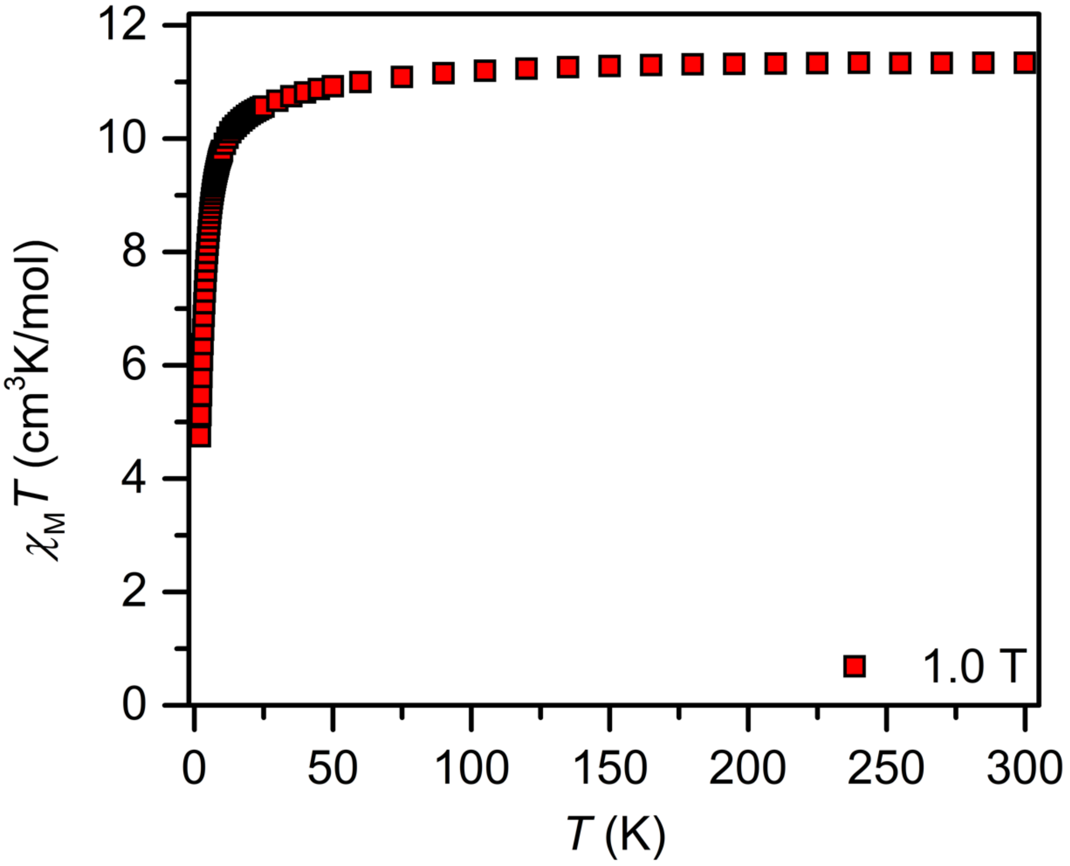

Figure S23. Variable-temperature dc magnetic susceptibility data of [K(crypt-222)][Er(dbCOT)$_2$], **1**, collected under a 1.0 T applied dc field.

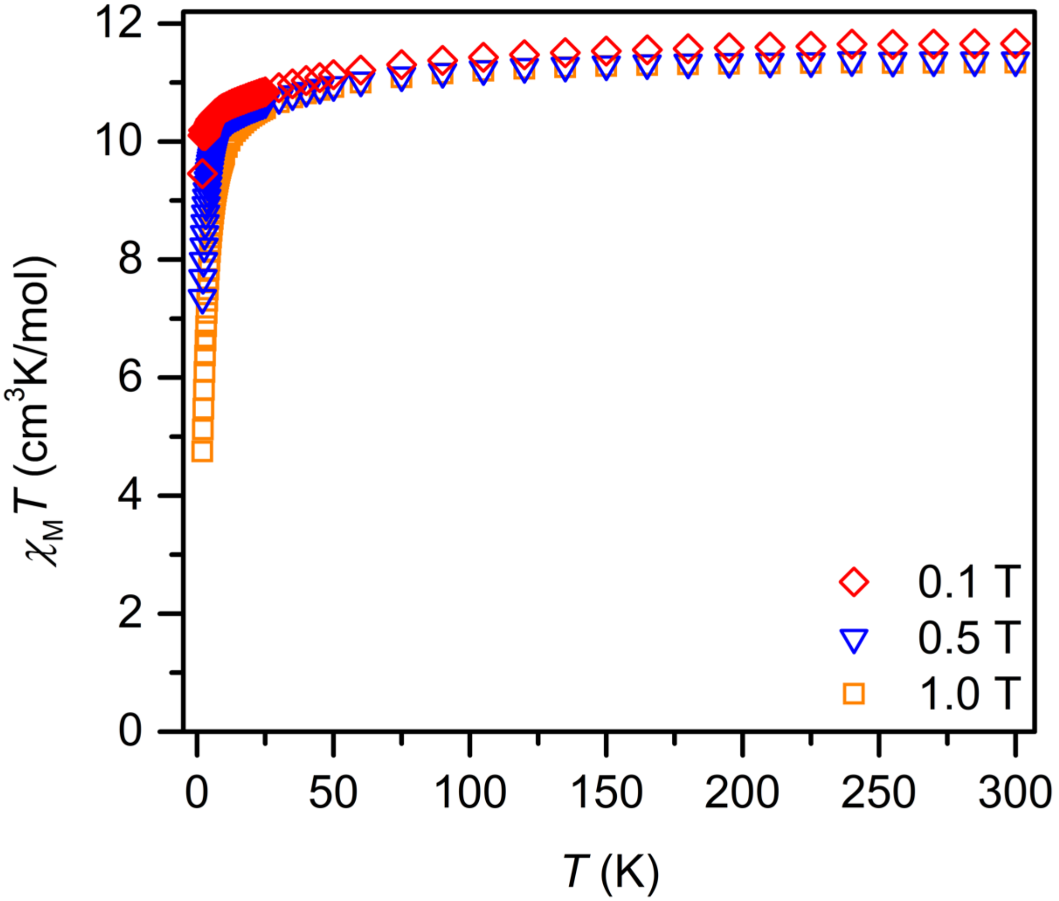

Figure S24. Variable-temperature dc magnetic susceptibility data of [K(crypt-222)][Er(dbCOT)$_2$], **1**, collected under 0.1 T (red diamonds), 0.5 T (blue triangles), and 1.0 T (orange squares) applied dc fields.

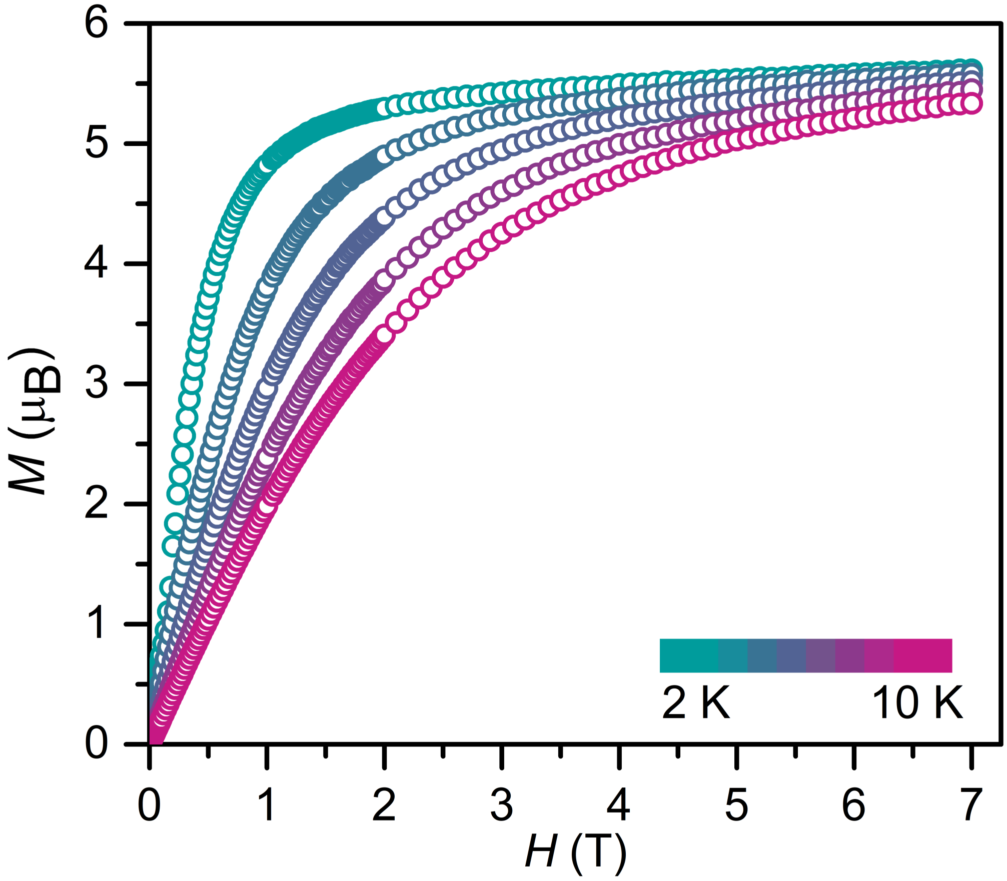

Figure S25. Field-dependent magnetization data for [K(crypt-222)][Er(dbCOT)$_2$], **1**, collected from 2 to 10 K, between 0 and 7 T.

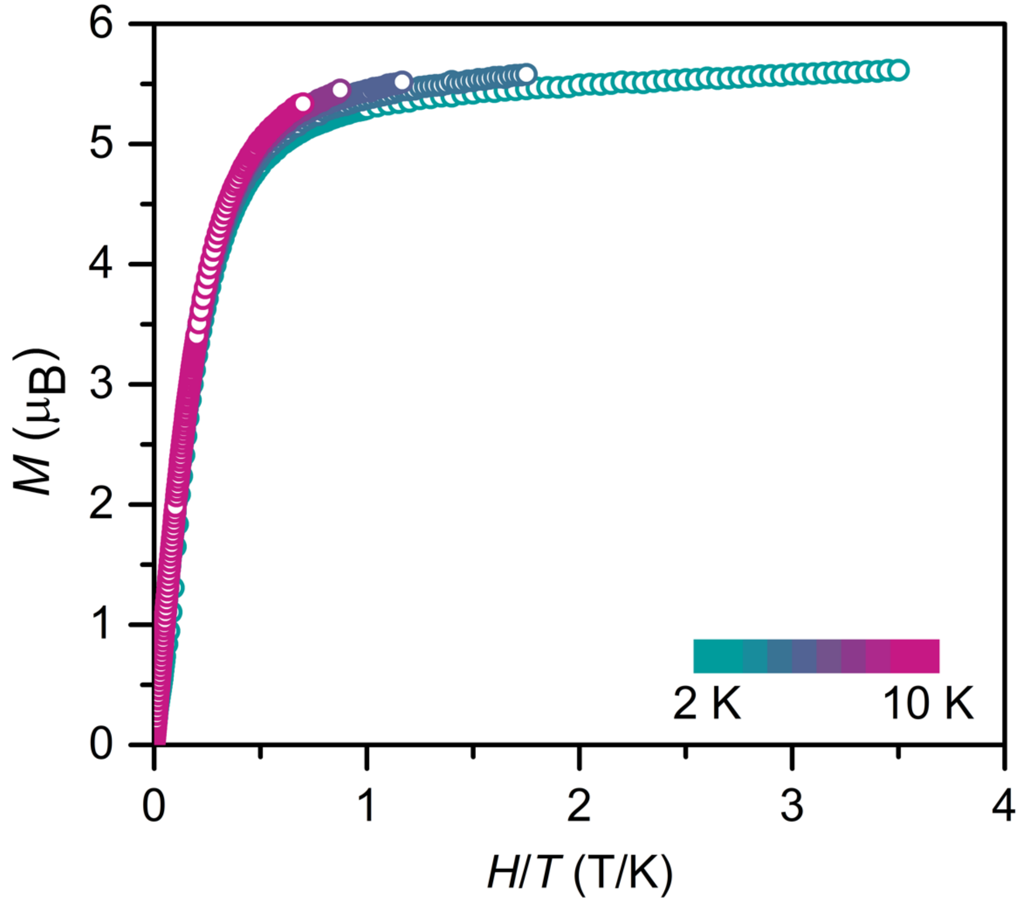

Figure S26. Reduced magnetization data for [K(crypt-222)][Er(dbCOT)$_2$], **1**, collected from 2 to 10 K, between 0 and 7 T.

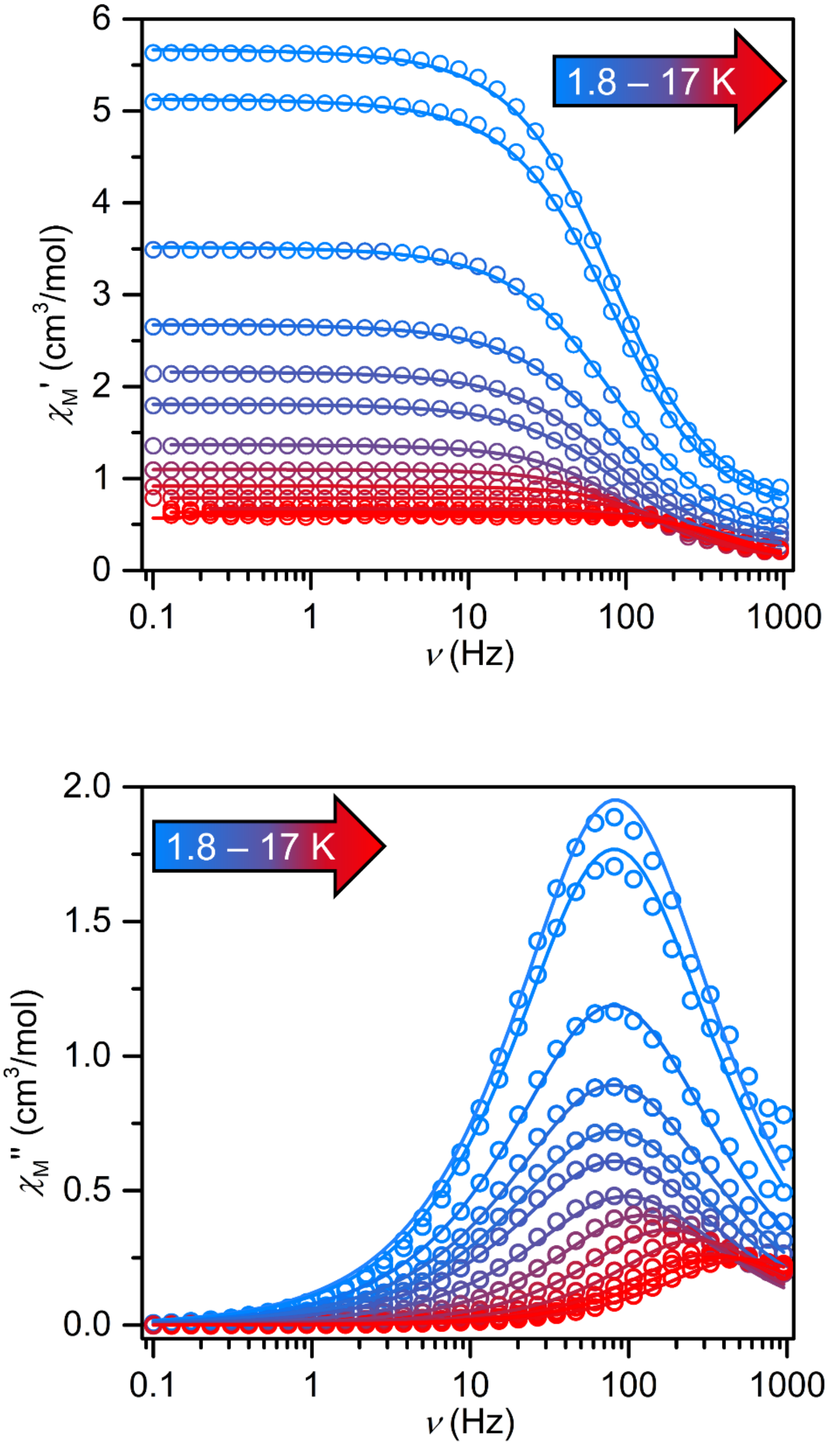

Figure S27. In-phase (χ_M') (top) and out-of-phase (χ_M'') components (bottom) of the ac magnetic susceptibility for [K(crypt-222)][Er(dbCOT)$_2$], **1**, under zero applied dc field from 1.8 K (blue circles) to 17 K (red circles). Solid lines represent fits of the data to a generalized Debye model.

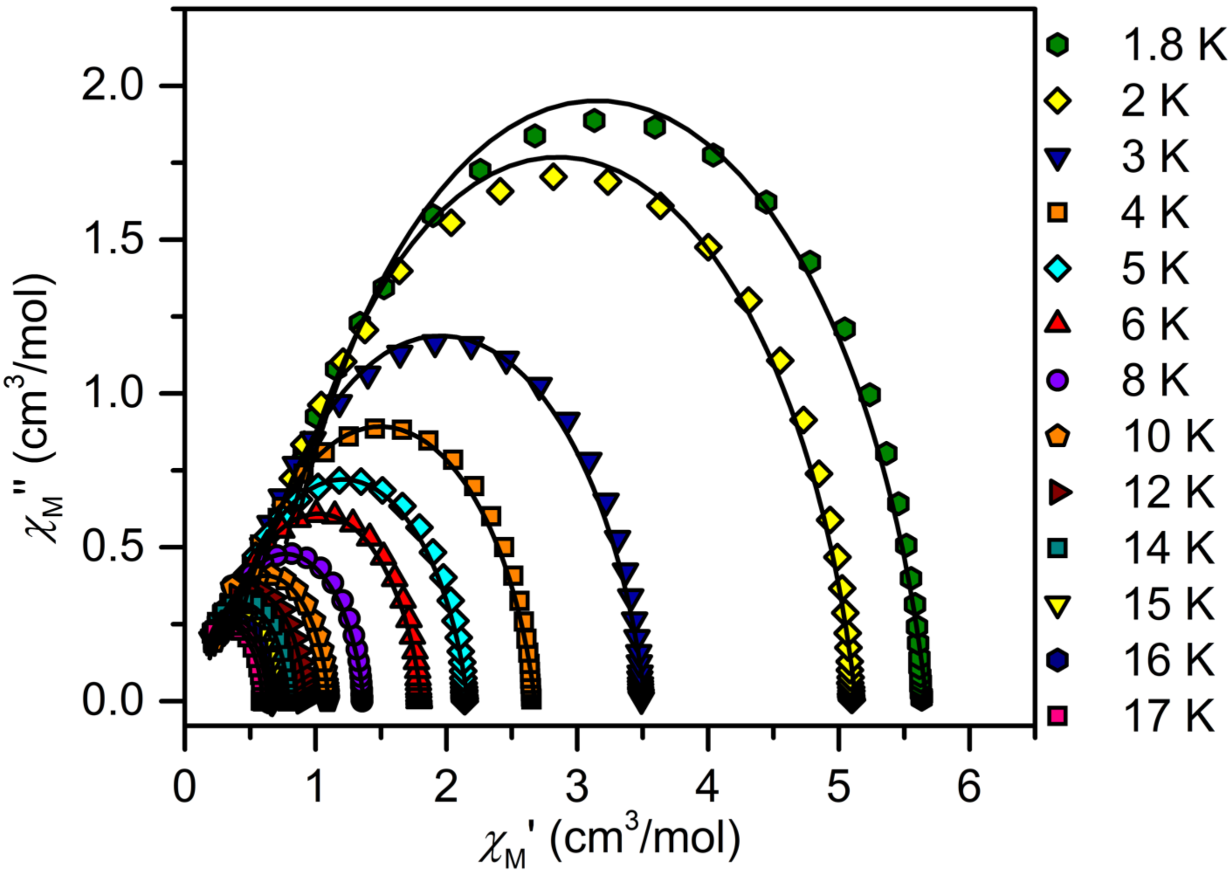

Figure S28. Cole-Cole plots for ac magnetic susceptibility of $[K(crypt\text{-}222)][Er(dbCOT)_2]$, **1**, measured under zero applied dc field from 1.8 K to 17 K. Symbols represent the experimental data points and the points representing the fits are connected by black solid lines.

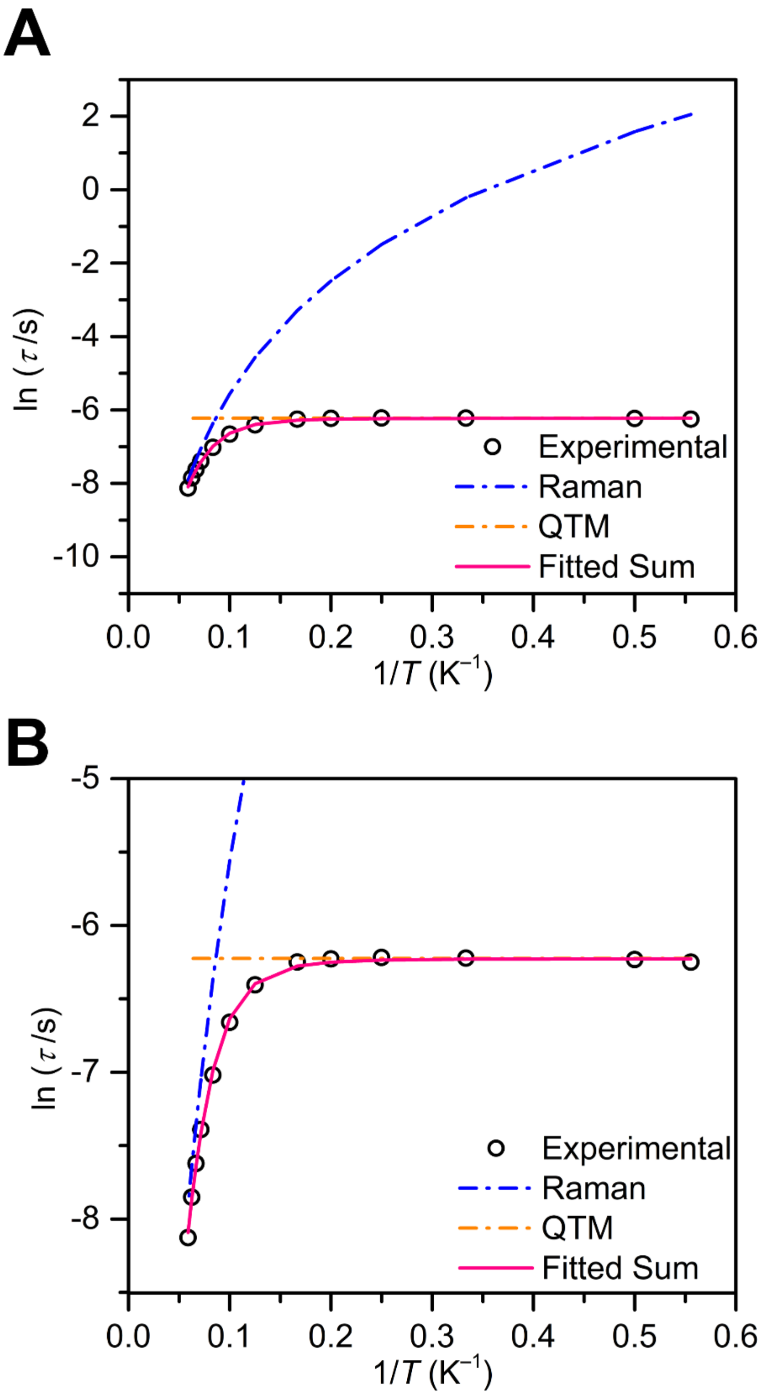

Figure S29. (**A**) Arrhenius plot of the relaxation time data, τ, vs. inverse temperature, 1/*T*, for [K(crypt-222)][Er(dbCOT)$_2$], **1**, under a zero applied dc field between 1.8 and 17 K. The blue and orange dashed lines represent fits to Raman and QTM processes, respectively, and yield the fitting parameters C = 9.49(1.3) x 10^{-3} $s^{-1}K^{-n}$, n = 4.44(9) and τ_{QTM}^{-1} = 1.98(1.0) x 10^{-3} s. The solid pink line represents the sum fit. (**B**) Magnification of the Arrhenius plot for **1** under a zero applied dc field between 1.8 and 17 K.

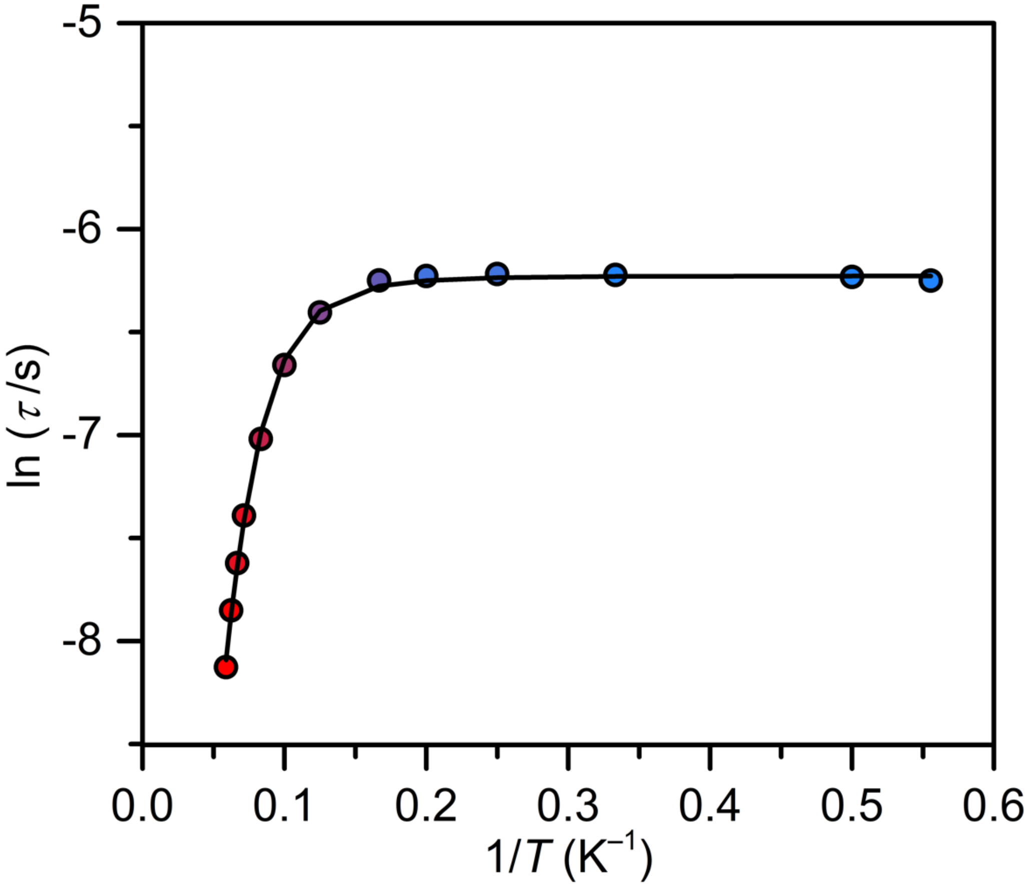

Figure S30. Arrhenius plot of the relaxation time data, τ, vs. inverse temperature, 1/*T*, for [K(crypt-222)][Er(dbCOT)$_2$], **1**, under zero applied dc field between 1.8 and 17 K. The solid black line represents fits to Raman and QTM processes.

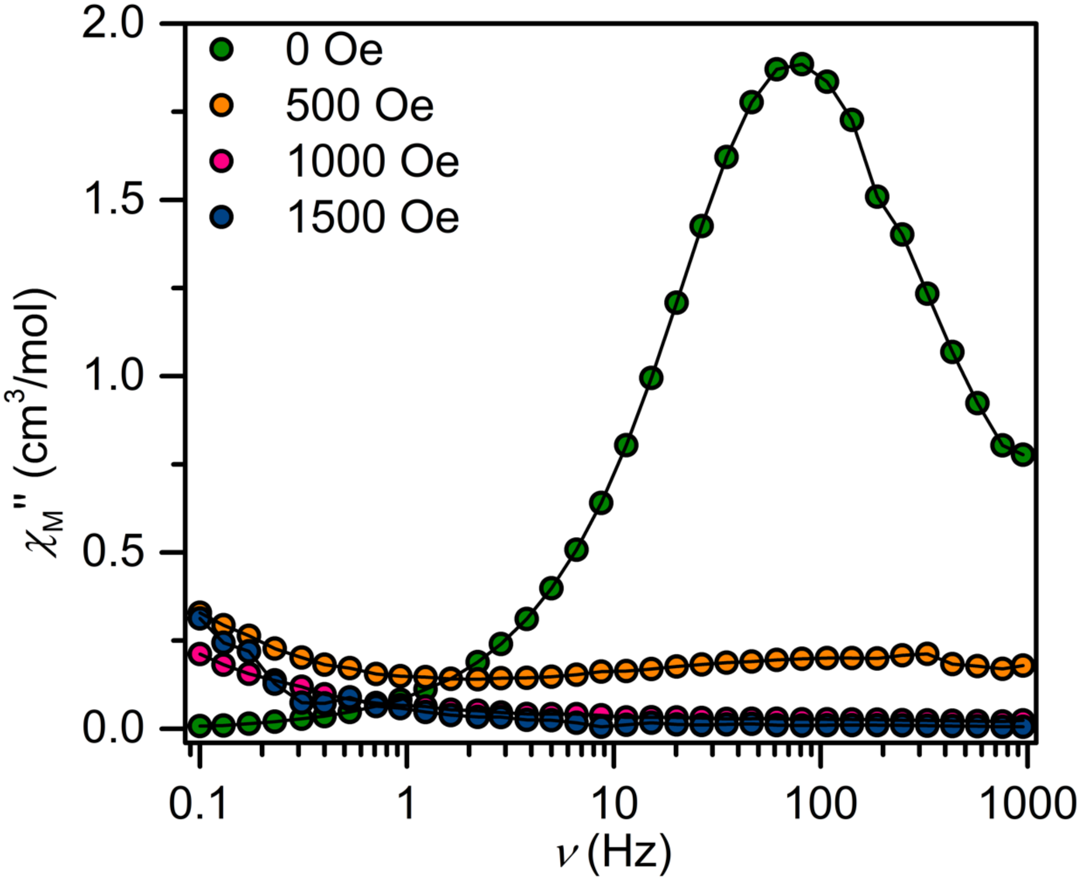

Figure S31. Out-of-phase (χ_M'') components of the ac magnetic susceptibility for [K(crypt-222)][Er(dbCOT)$_2$], **1**, at 1.8 K under dc fields from 0 to 1500 Oe in 500 Oe increments. Solid lines are guides for the eyes.

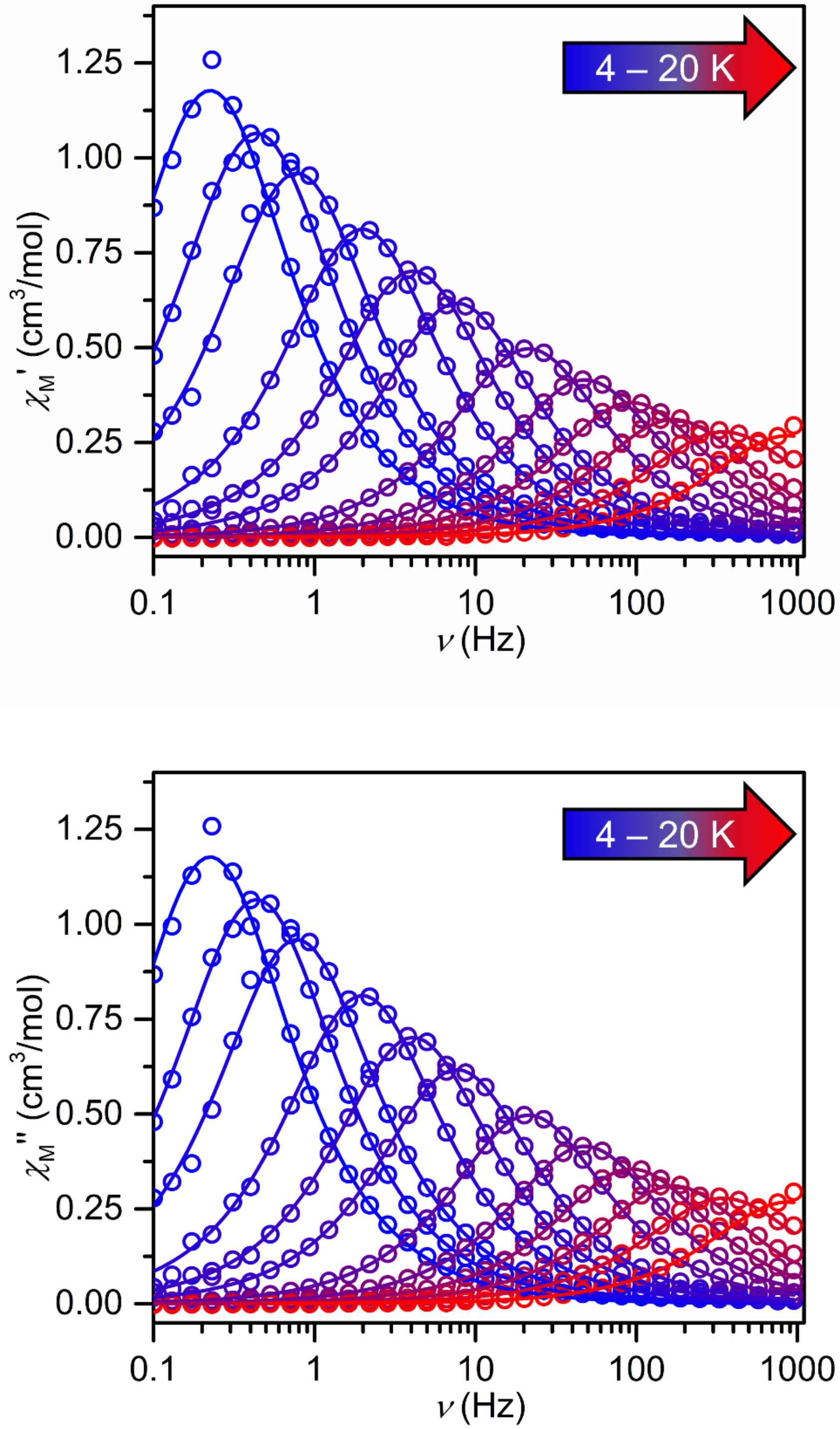

Figure S32. In-phase (χ_M′) (top) and out-of-phase (χ_M″) components (bottom) of the ac magnetic susceptibility for of the ac magnetic susceptibility for [K(crypt-222)][Er(dbCOT)$_2$], **1**, under a 1000 Oe applied dc field from 4 K (blue circles) to 20 (red circles). Solid lines represent fits of the data to a generalized Debye model.

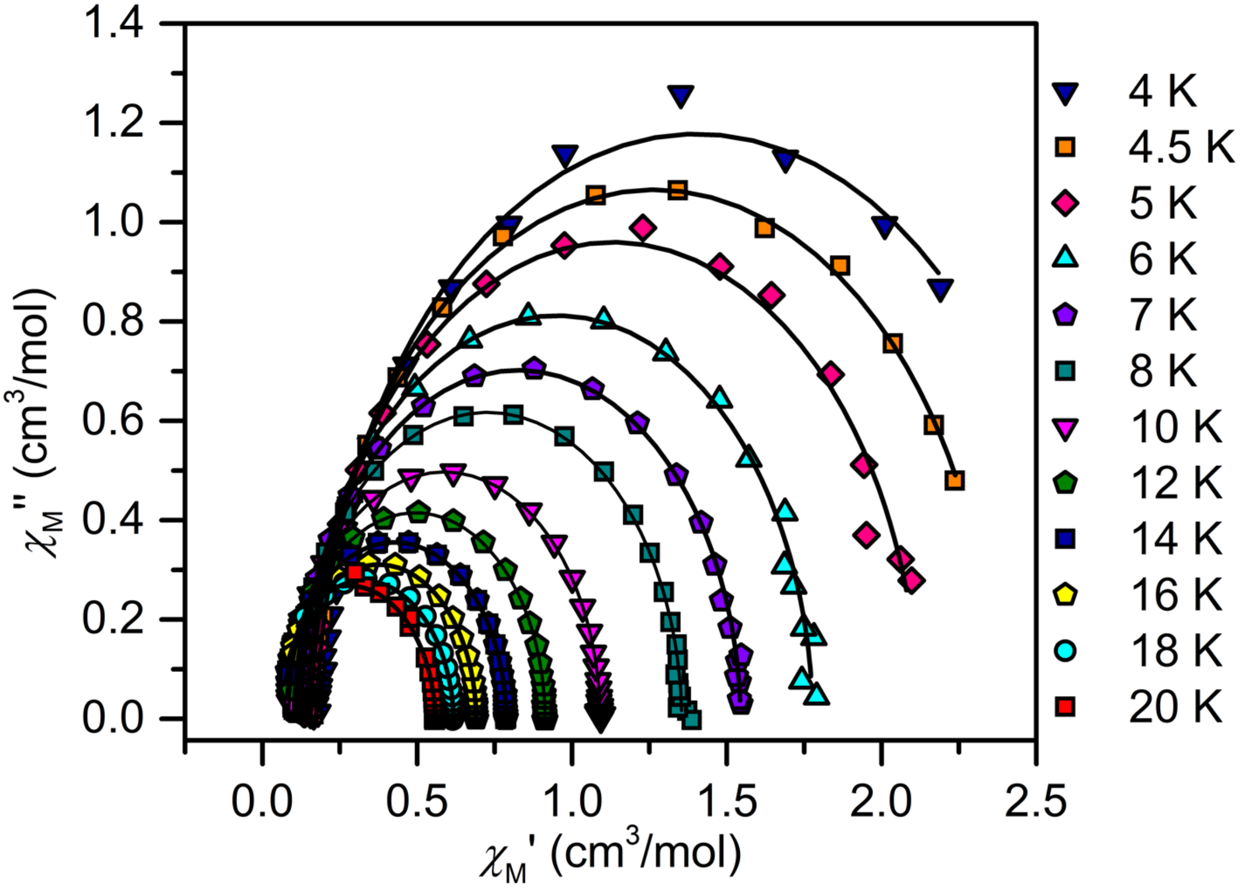

Figure S33. Cole-Cole plots for ac magnetic susceptibility of [K(crypt-222)][Er(dbCOT)$_2$], **1**, measured under a 1000 Oe applied dc field from 4 K to 20 K. Symbols represent the experimental data points and the points representing the fits are connected by black solid lines.

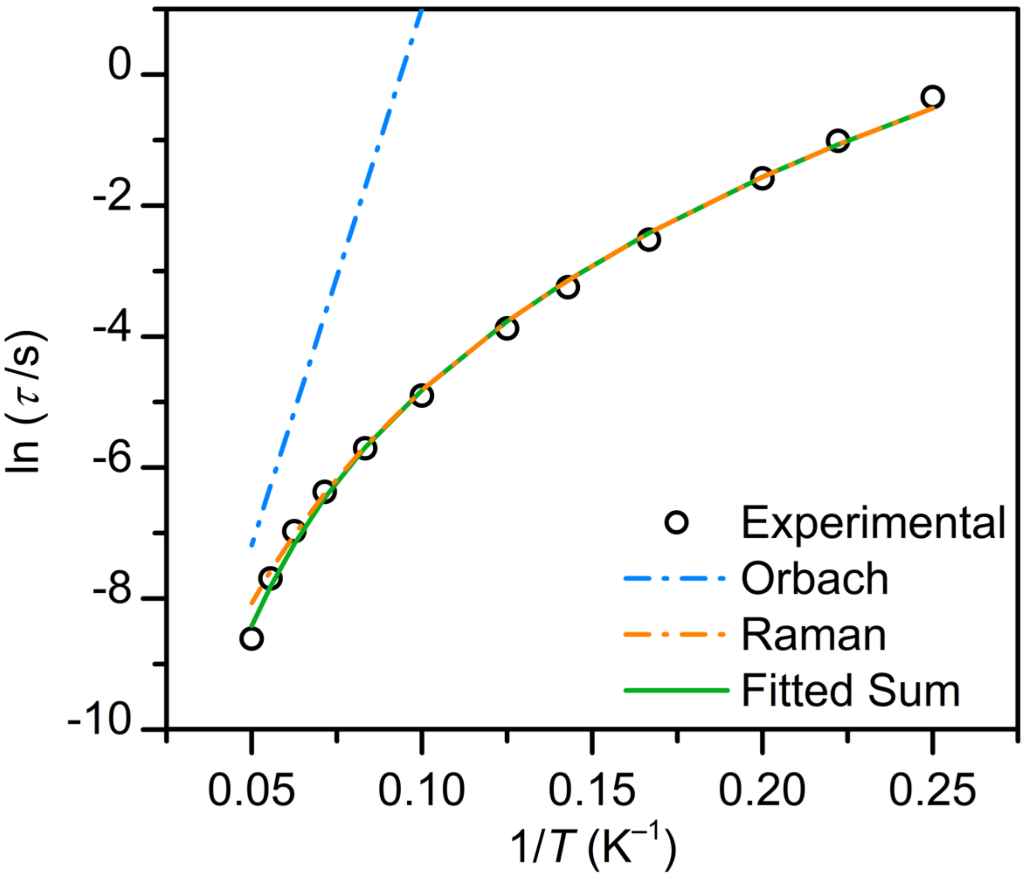

Figure S34. Arrhenius plot of the relaxation time data, τ, vs. inverse temperature, $1/T$, for $[K(crypt\text{-}222)][Er(dbCOT)_2]$, **1** (black circles), under 1000 Oe applied dc field between 4 and 20 K. The light blue and orange dashed lines represent a fit to Orbach and Raman processes, respectively, resulting in U_{eff} = 114(2) cm^{-1}, τ_0 = 2.1(1) x 10^{-7} s, C = 2.5(1) x 10^{-3}, and n = 4.7(2). The solid green line represents the fitted sum.

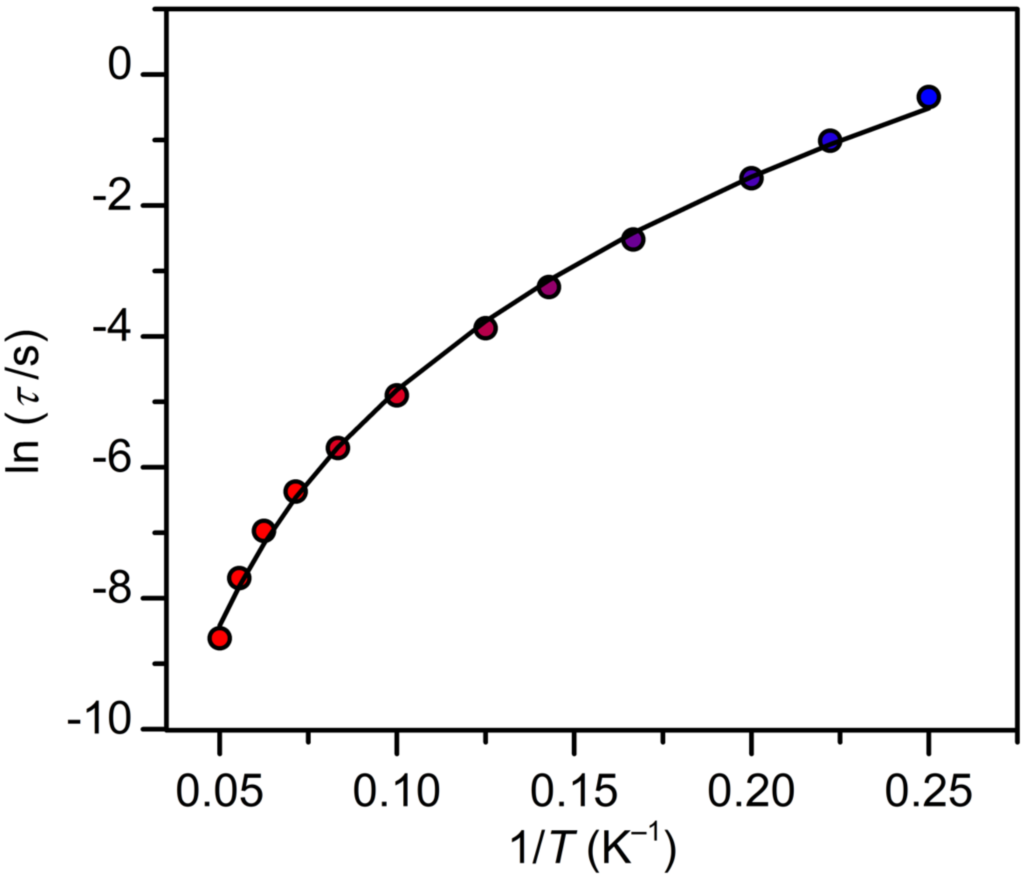

Figure S35. Arrhenius plot of the relaxation time data, τ, vs. inverse temperature, 1/*T*, for [K(crypt-222)][Er(dbCOT)$_2$], **1**, under a 1000 Oe applied dc field between 4 and 20 K. The solid black line represents fits to Orbach and Raman processes.

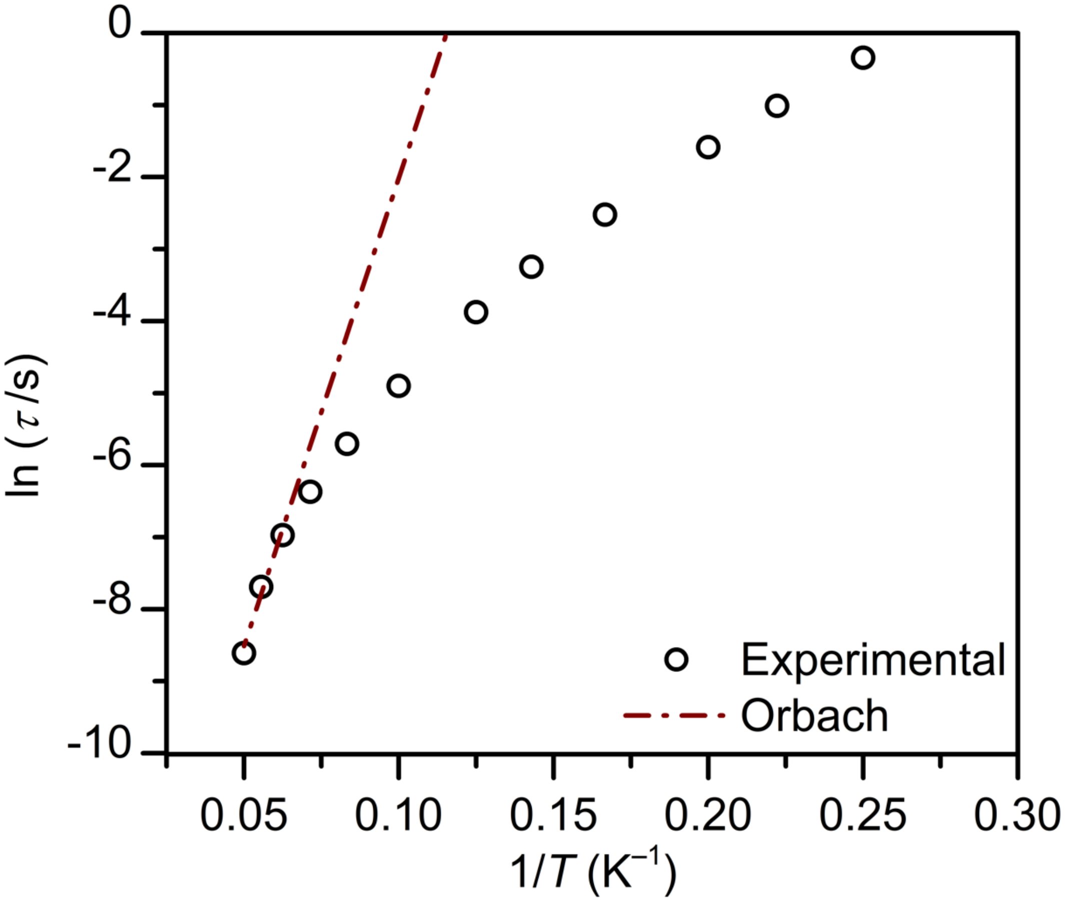

Figure S36. Arrhenius plot of the relaxation time data, τ, vs. inverse temperature, 1/*T*, for [K(crypt-222)][Er(dbCOT)$_2$], **1**, under a 1000 Oe applied dc field between 4 and 20 K. The solid purple line represents a fit to an Orbach process between 16 and 20 K, resulting in U_{eff} = 90.29 cm^{-1} and τ_0 = 2.94 x 10^{-7} s.

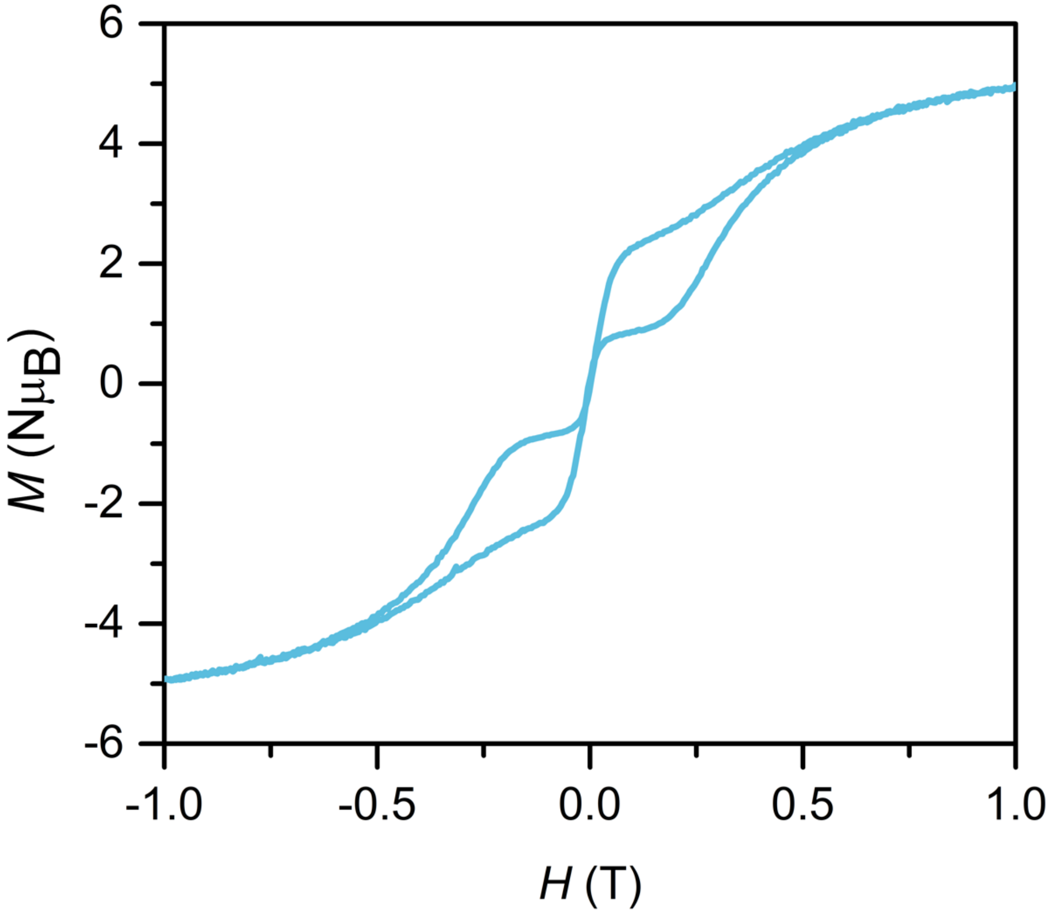

Figure S37. Variable-field magnetization data for [K(crypt-222)][Er(dbCOT)$_2$], **1**, collected at 1.8 K (pale blue) between –1 and +1 T, at an average sweep rate of 0.01 T/s. The widest region of the open butterfly-type hysteresis loop is approximately 0.2 T.

6 *Ab initio* Calculations

Table S7. Calculated Kramers doublet (KD) energies, associated magnetic moments, *g*-tensors, and wavefunction composition for the $[Er(dbCOT)_2]^-$ anion in a crystal of [K(crypt-222)]$[Er(dbCOT)_2]$, **1**. The angle θ between the ground state g_z axis and excited KDs g_z axis are given in degree (°). The wavefunction decompositions correspond to the lowest atomic multiplet *J* = 15/2 in wave functions with definite projection of the total moment to the quantization axis.

KD	*E* (cm^{-1})	*M* (μ_B)	g_x	g_y	g_z	θ (°)	Wave function composition
**1**	0.0	±8.947	0.00043	0.00054	17.89471	0.0	$\lvert\pm15/2\rangle$ (100%)
**2**	96.92	±6.589	1.33335	2.05460	13.17822	180.0	$\lvert\pm13/2\rangle$ (83%) $\lvert\pm1/2\rangle$ (15%)
**3**	109.13	±1.697	3.39376	5.72009	9.77762	90.0	$\lvert\pm13/2\rangle$ (16%) $\lvert\pm1/2\rangle$ (83%)
**4**	249.89	±1.905	2.10936	2.66964	3.80932	0.0	$\lvert\pm11/2\rangle$ (2%) $\lvert\pm3/2\rangle$ (96%) $\lvert\pm1/2\rangle$ (2%)
**5**	479.46	±3.058	0.85463	1.27219	6.11554	0.0	$\lvert\pm13/2\rangle$ (1%) $\lvert\pm5/2\rangle$ (98%)
**6**	504.55	±6.396	0.13132	0.28454	12.79128	180.0	$\lvert\pm11/2\rangle$ (97%) $\lvert\pm3/2\rangle$ (2%)
**7**	660.36	±4.177	0.67988	1.39526	8.35325	180.0	$\lvert\pm7/2\rangle$ (99%)
**8**	687.48	±5.332	0.12857	0.57287	10.66484	0.0	$\lvert\pm9/2\rangle$ (99%)

Table S8. Crystal field parameters calculated for the $[Er(dbCOT)_2]^-$ anion in a crystal of [K(crypt-222)]$[Er(dbCOT)_2]$, **1** via the SINGLE_ANISO program. The Hamiltonian employed to calculate the crystal field parameters is given by:

$$\hat{H}_{CF} = \sum \sum_{k=-q}^{+q} [B_k^q \hat{O}_k^q)]$$

where $\hat{O}_k^q$ is the extended Stevens operator, B_k^q the crystal field parameter, *k* is the rank of the irreducible tensor operator (ITO) (2,4,6), *q* is the component of the ITO (*q* = −*k*, −*k*+1,…0,1,…*k*).

k	*q*	Weight (%)	B_q^k	*k*	*q*	Weight (%)	B_q^k
	−2	<1	1.50E−02		−6	<1	3.58E−05
	−1	<1	4.41E−05		−5	<1	−3.44E−08
2	0	15.9	−1.66E+00		−4	1.6	−5.50E−05
	1	<1	−6.68E−04		−3	<1	2.26E−08
	2	3.6	4.56E−01		−2	<1	−3.06E−05
	−4	1.2	−2.87E−03		−1	<1	2.56E−08
	−3	<1	−3.95E−06	6	0	22.1	1.36E−04
	−2	<1	1.62E−04		1	<1	1.97E−07
	−1	<1	−1.43E−06		2	1.5	4.83E−05
4	0	31.8	−1.83E−02		3	<1	6.66E−07
	1	<1	−6.99E−06		4	5.9	2.02E−04
	2	<1	1.71E−03		5	<1	1.05E−06
	3	<1	3.30E−05		6	1.8	−8.46E−05
	4	4.2	1.02E−02				

Table S9. Calculated average transition dipole moments for the eight lowest lying Kramers doublets with opposing magnetization (+*I* → *I*−1), and for excited states (*I* → *I*+1), (*I* → *I*+2), (*I* → *I*+3), (*I* → *I*+4) and (*I* → *I*+5) for the $[Er(dbCOT)_2]^-$ anion in a crystal of $[K(crypt\text{-}222)][Er(dbCOT)_2]$, **1**.

**Through Barrier (+*I* → −*I*)**			**Through Excited States (+*I* → *I*+1)**			**Through Excited States (+*I* → *I*+2)**		
**KD1**	**KD2**	**Magnitude**	**KD**	**KD+1**	**Magnitude**	**KD**	**KD+2**	**Magnitude**
+1	−1	1.62E−04	+1	+2	1.42E+00	+1	+3	6.04E−01
+2	−2	5.65E−01	+1	−2	2.40E−04	+1	−3	1.32E−03
+3	−3	2.59E+00	+2	+3	8.63E−01	+2	+4	1.19E+00
+4	−4	7.97E−01	+2	−3	1.21E+00	+2	−4	4.46E−02
+5	−5	3.57E−01	+3	+4	2.78E+00	+3	+5	9.09E−02
+6	−6	7.39E−02	+3	−4	1.04E−01	+3	−5	2.62E−01
+7	−7	3.59E−01	+4	+5	2.97E+00	+4	+6	2.31E−01
+8	−8	1.25E−01	+4	−5	1.57E−02	+4	−6	8.43E−02
			+5	+6	7.08E−01	+5	+7	2.88E+00
			+5	−6	6.40E−02	+5	−7	1.47E−01
			+6	+7	5.23E−02	+6	+8	2.45E+00
			+6	−7	7.27E−02	+6	−8	5.49E−02
			+7	+8	2.74E+00			
			+7	−8	7.24E−02			

**Through Excited States (+*I* → *I*+3)**			**Through Excited States (+*I* → *I*+4)**			**Through Excited States (+*I* → *I*+5)**		
**KD**	**KD+3**	**Magnitude**	**KD**	**KD+4**	**Magnitude**	**KD**	**KD+5**	**Magnitude**
+1	+4	8.18E−03	+1	+5	7.37E-02	+1	+6	2.39E-02
+1	−4	3.09E−03	+1	−5	4.93E-03	+1	−6	2.35E-03
+2	+5	1.42E−01	+2	+6	1.85E+00	+2	+7	2.53E-01
+2	−5	1.07E−01	+2	−6	2.27E-02	+2	−7	5.88E-04
+3	+6	8.53E−01	+3	+7	9.94E-02	+3	+8	6.80E-02
+3	−6	5.63E−02	+3	−7	1.32E-02	+3	−8	8.81E-02
+4	+7	2.95E−02	+4	+8	2.69E-01			
+4	−7	1.33E−01	+4	−8	4.06E-02			
+5	+8	3.47E−02						
+5	−8	9.73E−02						

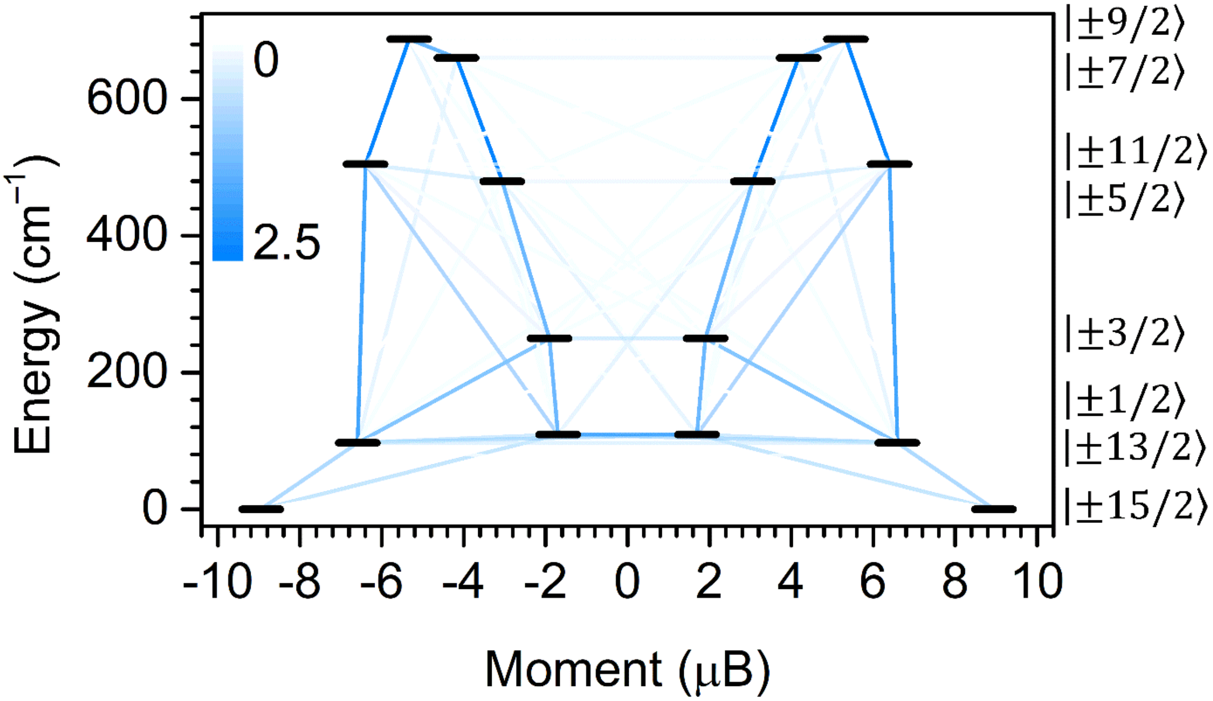

Figure S38. Calculated relaxation barrier for the $[Er(dbCOT)_2]^-$ anion in a crystal of $[K(crypt\text{-}222)][Er(dbCOT)_2]$, **1**. Arrows represent possible relaxation processes as indicated by calculated transition dipole moments, where dark blue coloration represents most probable transitions and faded blue indicates vanishing probabilities.

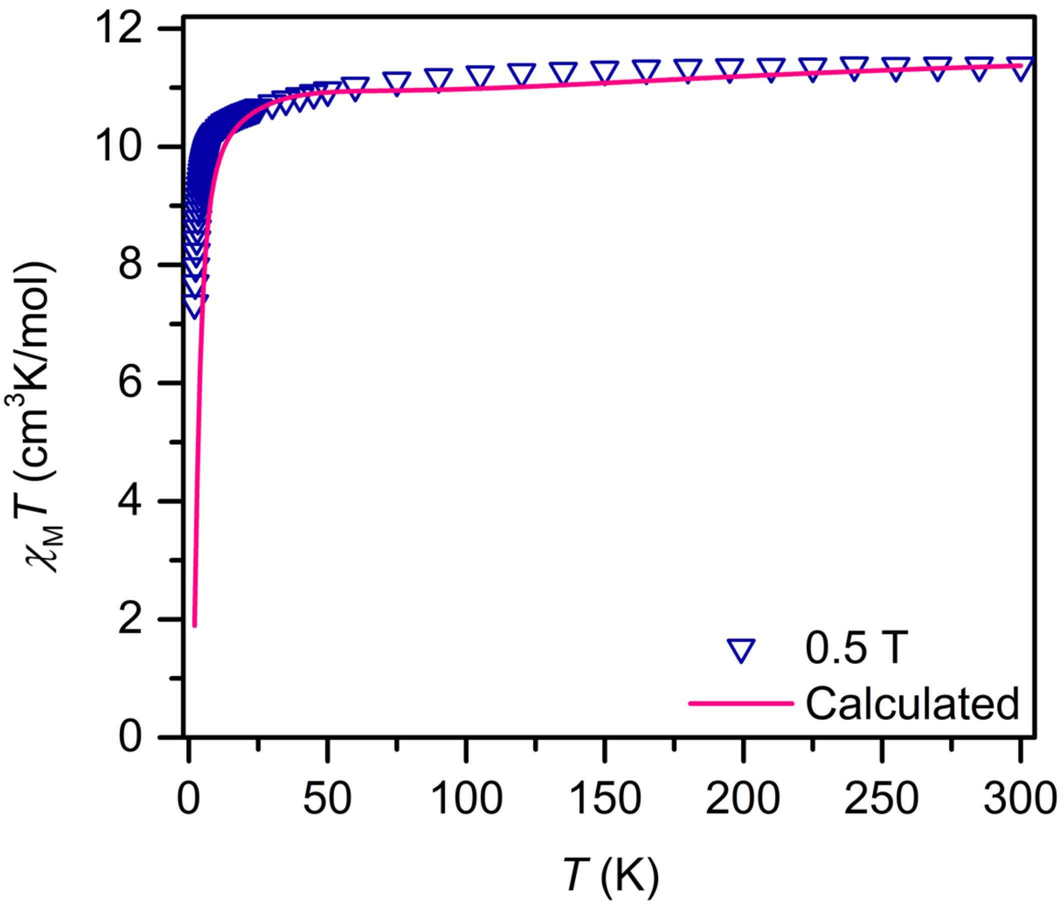

Figure S39. Variable-temperature dc magnetic susceptibility data of [K(crypt-222)][Er(dbCOT)$_2$], **1**, collected under a 0.5 T applied dc field (blue triangles). Solid pink line represents the calculated values for the [Er(dbCOT)$_2$]$^-$ anion.

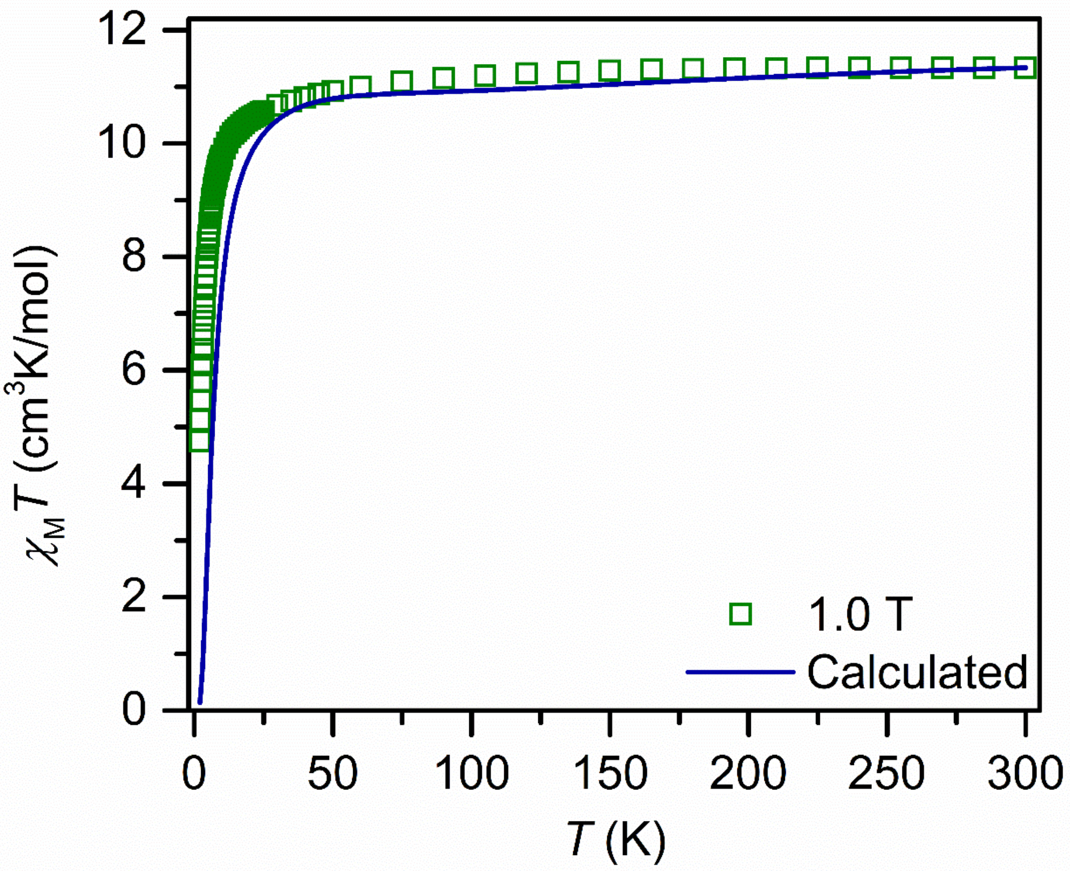

Figure S40. Variable-temperature dc magnetic susceptibility data of [K(crypt-222)][Er(dbCOT)$_2$], **1**, collected under a 1.0 T applied dc field (green squares). Solid dark blue line represents the calculated values for the [Er(dbCOT)$_2$]$^-$ anion.

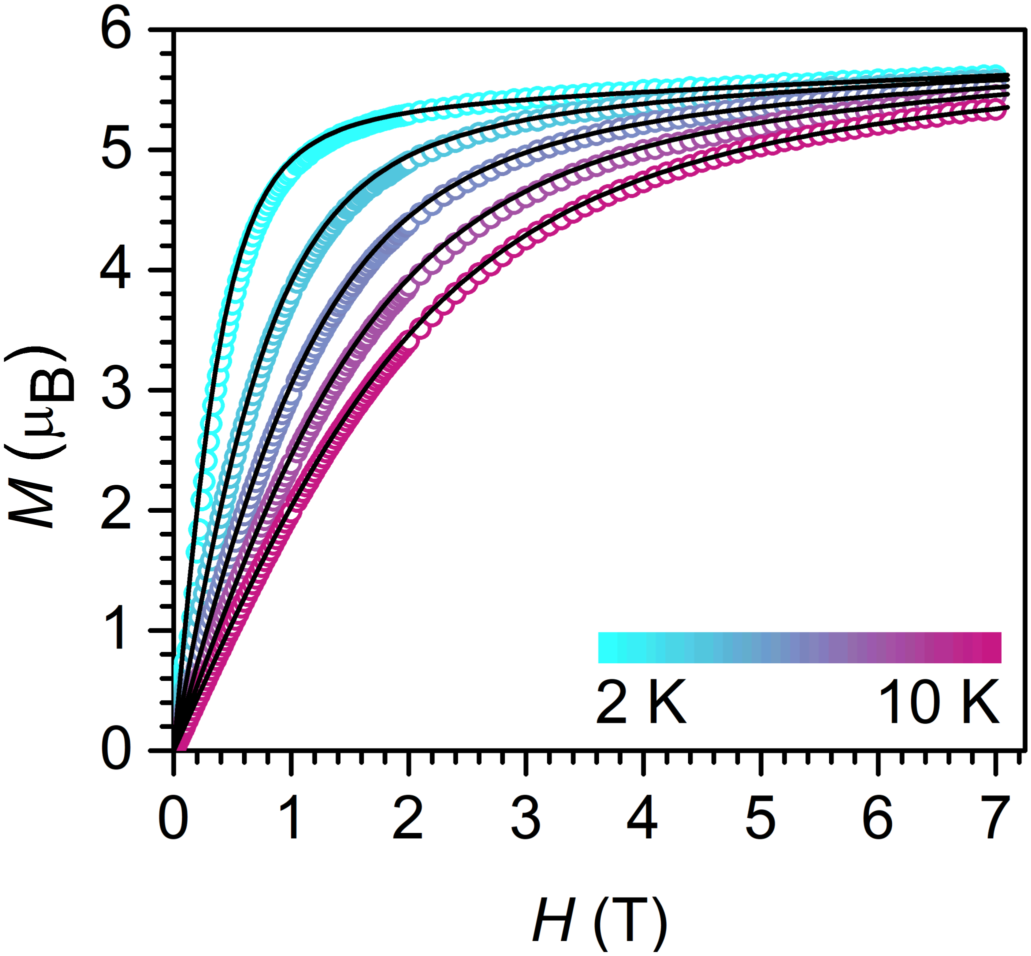

Figure S41. Field-dependent magnetization data for [K(crypt-222)][Er(dbCOT)$_2$], **1**, collected between 0 and 7 T at 2 K, 4 K, 6 K, 8K, and 10 K. Solid black lines represent the calculated values for the [Er(dbCOT)$_2$]$^-$ anion.

7 References

(1) Meihaus, K. R.; Long, J. R. Magnetic Blocking at 10 K and a Dipolar-Mediated Avalanche in Salts of the Bis(η^8-Cyclooctatetraenide) Complex $[Er(COT)^2]^-$ *J. Am. Chem. Soc.* **2013**, *135* (47), 17952–17957. https://doi.org/10.1021/ja4094814.

(2) Ungur, L.; Leroy, J. J.; Korobkov, I.; Murugesu, M.; Chibotaru, L. F. Fine-Tuning the Local Symmetry to Attain Record Blocking Temperature and Magnetic Remanence in a Single-Ion Magnet. *Angew. Chem. Int. Ed.* **2014**, *53* (17), 4413–4417. https://doi.org/10.1002/anie.201310451.

(3) Le Roy, J. J.; Korobkov, I.; Murugesu, M. A Sandwich Complex with Axial Symmetry for Harnessing the Anisotropy in a Prolate Erbium(III) Ion. *Chem. Commun.* **2014**, *50* (13), 1602–1604. https://doi.org/10.1039/C3CC48557A.

(4) Xue, T.; Ding, Y.-S.; Reta, D.; Chen, Q.-W.; Zhu, X.; Zheng, Z. Closely Related Organometallic Er(III) Single-Molecule Magnets with Sizably Different Relaxation Times of Quantum Tunneling of Magnetization. *Cryst. Growth Des.* **2023**, *23* (1), 565–573. https://doi.org/10.1021/acs.cgd.2c01177.

(5) Hilgar, J. D.; Butts, A. K.; Rinehart, J. D. A Method for Extending AC Susceptometry to Long-Timescale Magnetic Relaxation. *Phys. Chem. Chem. Phys.* **2019**, *21* (40), 22302–22307. https://doi.org/10.1039/C9CP03936H.

(6) Chen, Q.-W.; Ding, Y.; Xue, T.; Zhu, X.; Zheng, Z. A Hundredfold Enhancement of Relaxation Times among Er(III) Single-Molecule Magnets with Comparable Energy Barriers. *Inorg. Chem. Front.* **2023**, *10* (21), 6236.–6244. https://doi.org/10.1039/D3QI01361H.

(7) Orlova, A. P.; Varley, M. S.; Bernbeck, M. G.; Kirkpatrick, K. M.; Bunting, P. C.; Gembicky, M.; Rinehart, J. D. Molecular Network Approach to Anisotropic Ising Lattices: Parsing Magnetization Dynamics in Er^{3+} Systems with 0–3-Dimensional Spin Interactivity. *J. Am. Chem. Soc.* **2023**, *145* (40), 22265–22275. https://doi.org/10.1021/jacs.3c08946.

(8) Münzfeld, L.; Schoo, C.; Bestgen, S.; Moreno-Pineda, E.; Köppe, R.; Ruben, M.; Roesky, P. W. Synthesis, Structures and Magnetic Properties of $[(\eta^9\text{-}C_9H_9)Ln(\eta^8\text{-}C_8H_8)]$ Super Sandwich Complexes. *Nat. Commun.* **2019**, *10* (1), 3135–3135. https://doi.org/10.1038/s41467-019-10976-6.

(9) Wang, B.; Sun, H.; Wang, Z.; Gao, S. An Organometallic Single-Ion Magnet. *J. Am. Chem. Soc.* **2011**, *133*, (13), 4730–4733. https://doi.org/10.1021/ja200198v

(10) Münzfeld, L.; Dahlen, M.; Hauser, A.; Mahieu, N.; Kuppusamy, S. K.; Moutet, J.; Tricoire, M.; Köppe, R.; La Droitte, L.; Cador, O.; Le Guennic, B.; Nocton, G.; Moreno-Pineda, E.; Ruben, M.; Roesky, P. W. Molecular Lanthanide Switches for Magnetism and Photoluminescence. *Angew. Chem. Int. Ed.* **2023**, *62* (18), e202218107. https://doi.org/10.1002/anie.202218107.

(11) He, M.; Chen, X.; Bodenstein, T.; Nyvang, A.; Schmidt, S. F. M.; Peng, Y.; Moreno-Pineda, E.; Ruben, M.; Fink, K.; Gamer, M. T.; Powell, A. K.; Roesky, P. W. Enantiopure Benzamidinate/Cyclooctatetraene Complexes of the Rare-Earth Elements: Synthesis, Structure, and Magnetism. *Organometallics* **2018**, *37* (21), 3708–3717. https://doi.org/10.1021/acs.organomet.8b00412.

(12) Hilgar, J. D.; Bernbeck, M. G.; Flores, B. S.; Rinehart, J. D. Metal-Ligand Pair Anisotropy in a Series of Mononuclear Er-COT Complexes. *Chem. Sci.* **2018**, *9* (36), 7204–7209. https://doi.org/10.1039/C8SC01361F.
(13) Pan, F.; Sun, R.; Wang, B.-W.; Gao, S. Adaptability to Crystal Fields in a Series of COT/Monodentate Ligand-Based Dy and Er Single Ion Magnets. *Inorg. Chem.* **2023**, *62* (20), 8010–8018. https://doi.org/10.1021/acs.inorgchem.3c00922.
(14) Hilgar, J. D.; Bernbeck, M. G.; Rinehart, J. D. Million-Fold Relaxation Time Enhancement across a Series of Phosphino-Supported Erbium Single-Molecule Magnets. *J. Am. Chem. Soc.* **2019**, *141* (5), 1913–1917. https://doi.org/10.1021/jacs.8b13514.
(15) Orlova, A. P.; Hilgar, J. D.; Bernbeck, M. G.; Gembicky, M.; Rinehart, J. D. Intuitive Control of Low-Energy Magnetic Excitations via Directed Dipolar Interactions in a Series of Er(III)-Based Complexes. *J. Am. Chem. Soc.* **2022**, *144* (25), 11316–11325. https://doi.org/10.1021/jacs.2c03236.
(16) Chen, S.-M.; Xiong, J.; Zhang, Y.-Q.; Yuan, Q.; Wang, B.-W.; Gao, S. A Soft Phosphorus Atom to “Harden” an Erbium(III) Single-Ion Magnet. *Chem. Sci.* **2018**, *9* (38), 7540–7545. https://doi.org/10.1039/C8SC01626G.
(17) De, S.; Mondal, A.; Ruan, Z.-Y.; Tong, M.-L.; Layfield, R. A. Dynamic Magnetic Properties of Germole-Ligated Lanthanide Sandwich Complexes. *Chem. Eur. J.* **2023**, *29* (37), e202300567. https://doi.org/10.1002/chem.202300567.
(18) Meng, Y.-S.; Wang, C.-H.; Zhang, Y.-Q.; Leng, X.-B.; Wang, B.-W.; Chen, Y.-F.; Gao, S. (Boratabenzene)(Cyclooctatetraenyl) Lanthanide Complexes: A New Type of Organometallic Single-Ion Magnet. *Inorg. Chem. Front.* **2016**, *3* (6), 828–835. https://doi.org/10.1039/C6QI00028B.
(19) Münzfeld, L.; Sun, X.; Schlittenhardt, S.; Schoo, C.; Hauser, A.; Gillhuber, S.; Weigend, F.; Ruben, M.; Roesky, P. W. Introduction of Plumbole to F-Element Chemistry. *Chem. Sci.* **2022**, *13* (4), 945–954. https://doi.org/10.1039/D1SC03805B.
(20) Zhu, D.; Wang, M.; Guo, L.; Shi, W.; Li, J.; Cui, C. Synthesis, Structure, and Magnetic Properties of Rare-Earth Benzoborole Complexes. *Organometallics* **2021**, *40* (15), 2394–2399. https://doi.org/10.1021/acs.organomet.1c00337.
